# Host Cell Factors in HIV Replication: Meta-Analysis of Genome-Wide Studies

**DOI:** 10.1371/journal.ppat.1000437

**Published:** 2009-05-29

**Authors:** Frederic D. Bushman, Nirav Malani, Jason Fernandes, Iván D'Orso, Gerard Cagney, Tracy L. Diamond, Honglin Zhou, Daria J. Hazuda, Amy S. Espeseth, Renate König, Sourav Bandyopadhyay, Trey Ideker, Stephen P. Goff, Nevan J. Krogan, Alan D. Frankel, John A. T. Young, Sumit K. Chanda

**Affiliations:** 1 Department of Microbiology, University of Pennsylvania School of Medicine, Philadelphia, Pennsylvania, United States of America; 2 Department of Biochemistry and Biophysics, University of California, San Francisco, California, United States of America; 3 HARC Center, San Francisco, California, United States of America; 4 Department of Cellular and Molecular Pharmacology, University of California, San Francisco, California, United States of America; 5 Conway Institute, University College Dublin, Ireland; 6 Department of Antiviral Research, Merck Research Laboratories, West Point, Pennsylvania, United States of America; 7 Infectious & Inflammatory Disease Center, Burnham Institute for Medical Research, La Jolla, California, United States of America; 8 Department of Bioengineering, University of California, San Diego, La Jolla, California, United States of America; 9 Howard Hughes Medical Institute, Department of Biochemistry and Molecular Biophysics, College of Physicians and Surgeons, Columbia University, New York, New York, United States of America; 10 Infectious Disease Laboratory, The Salk Institute for Biological Studies, La Jolla, California, United States of America; The Fox Chase Cancer Center, United States of America

## Abstract

We have analyzed host cell genes linked to HIV replication that were identified in nine genome-wide studies, including three independent siRNA screens. Overlaps among the siRNA screens were very modest (<7% for any pairwise combination), and similarly, only modest overlaps were seen in pairwise comparisons with other types of genome-wide studies. Combining all genes from the genome-wide studies together with genes reported in the literature to affect HIV yields 2,410 protein-coding genes, or fully 9.5% of all human genes (though of course some of these are false positive calls). Here we report an “encyclopedia” of all overlaps between studies (available at http://www.hostpathogen.org), which yielded a more extensively corroborated set of host factors assisting HIV replication. We used these genes to calculate refined networks that specify cellular subsystems recruited by HIV to assist in replication, and present additional analysis specifying host cell genes that are attractive as potential therapeutic targets.

## Introduction

Genome-wide screening technologies offer unprecedented opportunities for discovery [1], but each method is imperfect, so that correct calls will be mixed with false positives, and authentic functions will be missed at some frequency, yielding false negatives. For example, three small interfering RNA (siRNA) screens have been reported that interrogated most of the human genes for effects on HIV infection [2]–[5], but though these screens identified many cellular factors previously implicated in HIV replication, the overlap between any pair of screens was <7%. The siRNA method has many limitations [6],[7]. For a gene to be detected as important during HIV infection, it must meet the following criteria: 1) It must be possible to achieve biologically meaningful reduction in mRNA levels with the siRNA(s) used. 2) The protein must be sufficiently unstable to allow functionally significant reduction over the time course tested. 3) The knockdown must not be toxic. 4) The function targeted cannot be provided by multiple redundant factors. In addition, genes may be called mistakenly due to experimental errors during high throughput analysis or off-target activities of the siRNAs used. Furthermore, siRNAs that do pass all of the above hurdles and affect viral infection may target factors that act only indirectly. Other screening technologies are also fraught with experimental limitations. However, genes identified independently in multiple studies should have a greater chance of being correctly called. Here, we report a meta-analysis of nine genome-wide screens for cellular factors associated with HIV replication.

## Genome-Wide Surveys Used in the Meta-Analysis

We analyzed human gene products identified as important for HIV infection in the nine screens presented in Table 1. Three screens (lists 1–3) used transfection of siRNAs to knock down >20,000 human genes, then assessed the efficiency of HIV infection. The König et al. study [3] (list 1) used 293T cells as targets and only examined the steps of uncoating through viral gene expression. This study used a relatively large number of siRNAs per gene and included extensive mapping of the effects of knockdown to steps in the HIV replication cycle. The Brass et al. [2] (list 2) and Zhou et al. [4] (list 3) studies used HeLa cells as targets, and had the advantage of examining all the steps of HIV replication, though with less redundant siRNA coverage. List 4 contains genes near human polymorphisms identified by Fellay et al. as associated with different HIV viral loads in patients [8]. List 5 is composed of genes encoding proteins found in HIV particles that budded out of monocyte-derived macrophages [9]. Lists 6–9 contain raw screening data on binding interactions between HIV proteins and cellular proteins identified using a pull-down mass spectrometry approach (lists 6–8, targeting Nef, Tat, and Rev) or yeast two-hybrid analysis (list 9, targeting IN) [10]. List 10 summarizes interactions between HIV and cellular proteins from the published literature [11]—the depth and quality of the literature is quite variable among these proposed cellular factors, but for this analysis all calls were treated equally. Lists 11 and 12 contain siRNA screen data for two additional viruses (influenza virus studied in fly cells [12], and West Nile Virus studied in human cells [13]), allowing comparison with HIV. Report S1 presents more detailed descriptions of the 12 data sets along with extensive overlap analysis (also available at http://www.hostpathogen.org).

**Table 1:** Gene Sets Analyzed.

List	Name	Number of Genes	Description	Reference
1	siRNA HIV König	293	siRNA screen for host factors promoting HIV replication	[3]
2	siRNA HIV Brass	283	siRNA screen for host factors promoting HIV replication	[2]
3	siRNA HIV Zhou	303	siRNA screen for host factors promoting HIV replication	[4]
4	SNP HIV Fellay	63	GWA study of HIV set point in infected individuals	[7]
5	Particle Associated HIV	248	Proteins in HIV particles identified by mass spec	[8]
6	HARC Nef	6	Cellular proteins that interact with HIV Nef protein (mass spec)	This work
7	HARC Tat	69	Cellular proteins that interact with HIV Tat protein (mass spec)	This work
8	HARC Rev	56	Cellular proteins that interact with HIV Rev protein (mass spec)	This work
9	BIND INT HIV	23	Cellular proteins that interact with HIV IN protein (yeast two hybrid)	[9]
10	NCBI Interactions	1434	Published interactions between an HIV protein and a cellular protien	[10]
11	siRNA Flu Fly	98	Human homologs of fly gene products important for influenza virus infection	[11]
12	siRNA WNV	305	Gene products important for West Nile virus infection	[12]

## Overview of Genes Proposed to Be Associated with HIV Infection

A total of 1,254 genes were called as important during HIV infection in at least one genome-wide survey (lists 1–9 above), representing about 5% of all human protein-coding genes (using the RefSeq total number of 25,157). One measure of the accuracy of the genome-wide methods is assessing the overlap with genes previously identified in published peer-reviewed studies of HIV (list 10). Comparing the genes called in the HIV interaction database (list 10, 1,434 genes) to those identified in the nine genome-wide surveys (lists 1–9) yielded an overlap of only 257 genes. The union of all genes called in the genome-wide studies and the National Center for Biotechnology Information (NCBI) interaction database (lists 1–10) contains a remarkable 2,393 human protein-coding genes associated with HIV infection, or 9.5% of all human genes.

**Figure 1:**
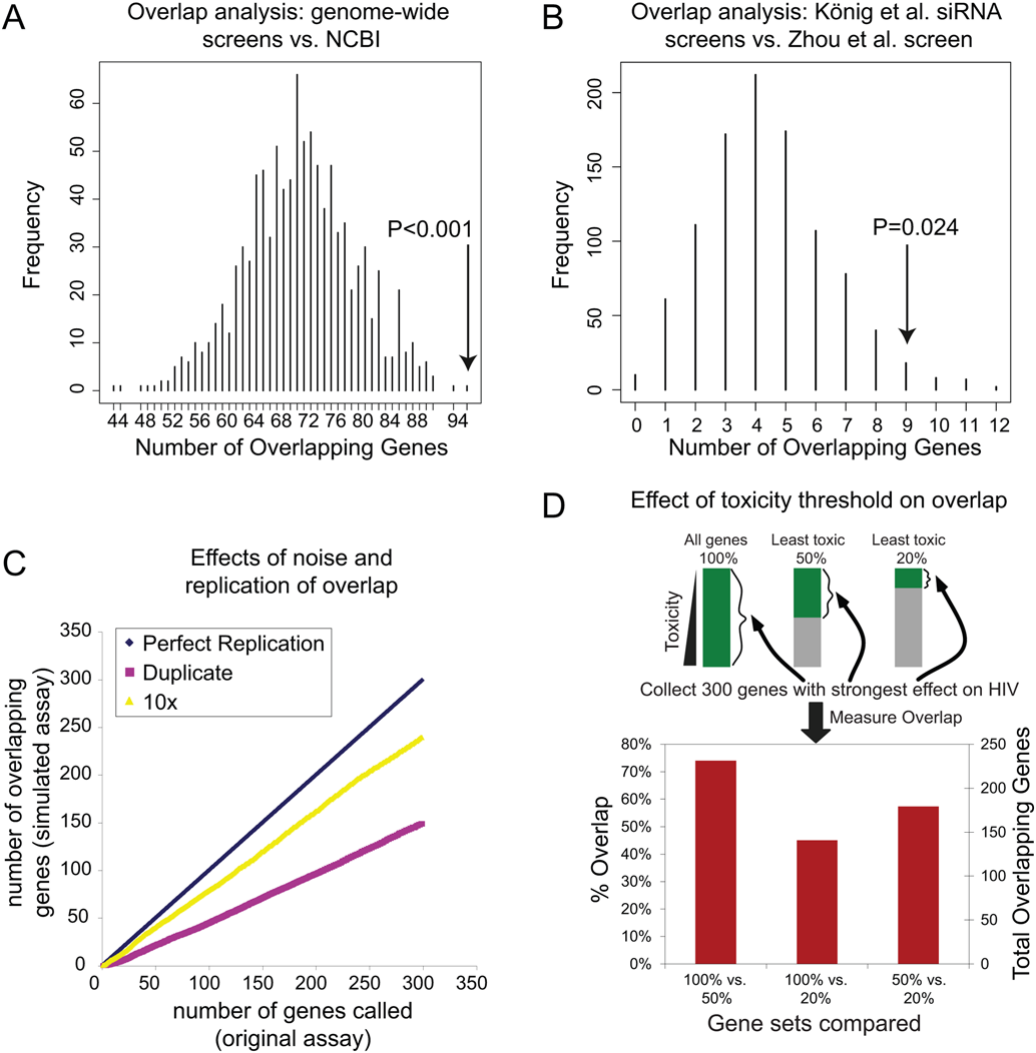
Overlap Analysis to Generate an Empirical *p*-Value. In each panel the downward arrow indicates the number of genes overlapping between the experimental data sets, and the bars show the frequencies of overlaps in comparisons to random distributions. (A) Simulation of overlap between all of the genes called in genome-wide screens (lists 1–9) and the NCBI database of factors reported to be involved in HIV replication (list 10). One thousand pairs of gene sets were drawn randomly from the set of all human genes, 1,254 to simulate the set of all genes from genome-side screens and 1,434 to simulate NCBI genes, and the overlap in each pair plotted. The experimental overlap was 257 genes. (B) Simulation of overlapping genes between the König et al. and Zhou et al. siRNA screens. The experimental overlap was nine genes. The *p*-value calculated using the hypergeometric distribution was slightly lower (*p* = 0.014). (C) Simulation of expected overlap between screens given the measured error between replicates. The standard deviation of infectivity measurements were calculated from the König et al. siRNA screens, and then simulated datasets were generated containing the measured error. For simulations, either two replicates (pink) or ten replicates (yellow) were generated and the overlap quantified. The *y*-axis: number of top-scoring genes considered in overlap analysis; *x*-axis: actual number of overlapping genes seen comparing simulated data sets. (D) Choices for toxicity threshold strongly influence the recovery of genes affecting HIV infection. The genes tested in the König et al. siRNA screen were ranked according to toxicity of knockdown, then sets containing 100% of genes, the least toxic 50%, or the least toxic 20% were extracted (top). From each of these, the 300 genes that when knocked down showed the strongest reduction in HIV infection were then selected, and the overlap between gene sets calculated (bottom).

Were the genes identified in the genome-wide screens (lists 1–9) even enriched at all for previously identified HIV interacting genes (list 10)? The significance of this overlap was assessed by comparison to a random distribution. For the NCBI list of HIV-interacting factors (list 10), 1,434 randomly selected genes were drawn 1,000 times with replacement from the background of all human genes, simulating the NCBI list, and 1,254 random genes were drawn 1,000 times from all human genes as well, simulating the genome-wide list. The overlaps of the 1,000 repetitions of the random draws were quantified and plotted (Figure 1A). The modal number of overlapping genes was 71, and no simulations showed an overlap of 257 or more genes, yielding a highly significant *p*-value (<0.001). Thus the overlap, though modest, is highly significant. Genes identified in lists 1–10 were analyzed in all pairwise combinations to identify genes in common between each pair (detailed data are in Report S1, pp. 5–70) and the significance tested by simulation or calculated as in [14]. An example is presented in Figure 1B, and the numbers of overlapping genes and their significance for all pairwise combinations of gene lists is shown in Table 2.

**Table 2:** Statistical Analysis of Genes in Common between All Pairs of Genome-Wide Studies.

Table Name (Size)	siRNA HIV König (293)	siRNA HIV Brass (283)	siRNA HIV Zhou (303)	siRNA HIV Fellay (63)	Particle Associated HIV (248)	HARC Nef (6)	HARC Tat (69)	HARC Rev (56)	BIND INT HIV (23)	NCBI Interactions (1,434)	siRNA Flu Fly (98)
siRNA HIV König (293)											
siRNA HIV Brass (283)	<0.001 (13)										
siRNA HIV Zhou (303)	0.024 (9)	<0.001 (18)									
siRNA HIV Fellay (63)	1 (0)	0.511 (1)	0.541 (1)								
Particle Associated HIV (248)	0.154 (5)	0.035 (6)	0.07 (6)	0.108 (2)							
HARC Nef (6)	1 (0)	1 (0)	1 (0)	1 (0)	<0.001 (2)						
HARC Tat (69)	1 (0)	0.004 (4)	0.052 (3)	1 (0)	0.027 (3)	1 (0)					
HARC Rev (56)	0.125 (2)	0.44 (1)	0.469 (1)	1 (0)	<0.001 (10)	1 (0)	1 (0)				
BIND INT HIV (23)	<0.001 (3)	1 (0)	0.232 (1)	1 (0)	0.191 (1)	1 (0)	1 (0)	0.07 (1)			
NCBI Interactions (1,434)	<0.001 (53)	<0.001 (39)	<0.001 (40)	0.234 (5)	<0.001 (94)	1 (0)	<0.001 (21)	<0.001 (21)	0.009 (5)		
siRNA Flu Fly (98)	<0.001 (13)	0.125 (3)	0.738 (1)	1 (0)	<0.001 (9)	1 (0)	1 (0)	0.002 (3)	1 (0)	<0.001 (20)	
siRNA WNV (305)	0.02 (8)	0.004 (9)	0.693 (3)	0.14 (2)	0.013 (8)	1 (0)	0.061 (3)	0.481 (1)	1 (0)	0.006 (29)	0.337 (2)

Each entry in the table shows the *p*-values (determined by comparison to random simulation) and the number of overlapping genes in parenthesis. Set names are as in Table 1.

## Analysis of Overlap among the Three siRNA Screens for Genes Affecting HIV Replication

The three siRNA screens (lists 1–3) together called 842 genes as diminishing HIV replication when knocked down, or 3.3% of all human protein-coding genes (Report S1, pp. 98–120). A total of 34 genes were called in at least two siRNA screens (Table 3). Three genes were called in all three screens (MED6, MED7, and RELA). The pairwise overlaps were statistically significant (*p*<0.024 for all pairs of screens), but the percentages of shared genes were quite modest, ranging from 3% to 6%. The Brass et al. and Zhou et al. screens (lists 2 and 3) both surveyed the entire HIV life cycle and studied infection in HeLa cells, and these two share the greatest overlap (6%). The three siRNA screens identified the NCBI genes as 13.3%–18.3% of the total, indicating highly significant enrichment (*p*<0.001), as reported previously.

**Table 3:** Genes Called in at Least Two siRNA Screens.

Symbol	Frequency (siRNA)	siRNA HIV König	siRNA HIV Brass	siRNA HIV Zhou	SNP HIV Fellay	Particle Associated HIV	HARC Nef	HARC Tat	HARC Rev	BIND INT HIV	NCBI Interactions	siRNA Flu Fly	siRNA WNV	Druggable?	Exp in CD4	Description
ADRBK1	2	*		*										yes	yes	Adrenergic, beta, receptor kinase 1
AKT1	2		*	*							*			yes	yes	v-akt murine thymoma viral oncogene homolog 1
CAV2	2		*	*											yes	Caveolin 2
CCNT1	2		*	*				*			*					Cyclin T1
CD4	2		*	*							*				yes	CD4 molecule
DDX3X	2		*	*				*	*		*				yes	DEAD (Asp-Glu-Ala-Asp) box polypeptide 3, X-linked
DMXL1	2	*	*												yes	Dmx-like 1
IDH1	2	*	*												yes	Isocitrate dehydrogenase 1 (NADP+), soluble
JAK1	2		*	*							*			yes	yes	Janus kinase 1 (a protein tyrosine kinase)
MAP4	2	*	*												yes	Microtubule-associated protein 4
MRE11A	2	*		*											yes	MRE11 meiotic recombination 11 homolog A (S. cerevisiae)
RANBP2	2	*	*											yes	yes	RAN binding protein 2
TCEB3	2		*	*							*				yes	Transcription elongation factor B (110 kDa, elongin A)
CXCR4	2		*	*							*			yes	yes	Chemokine (C-X-C motif) receptor 4
CHST1	2	*		*							*				yes	Carbohydrate (keratan sulfate Gal-6) sulfotransferase 1
CTDP1	2	*	*								*				yes	CTD (carboxy-terminal domain, RNA polymerase II
MED14	2	*	*												yes	Mediator complex subunit 14
RAB28	2		*	*											yes	RAB28, member RAS oncogene family
NUP153	2	*	*								*	*		yes	yes	Nucleoporin 153 kDa
TNPO3	2	*	*												yes	Transportin 3
MED4	2		*	*											yes	Mediator complex subunit 4
ANAPC2	2	*		*											yes	Anaphase promoting complex subunit 2
MID1IP1	2	*	*										*		yes	MID1 interacting protein 1 (gastrulation specific G12 homolog)
WNK1	2		*	*											yes	WNK lysine deficient protein kinase 1
RNF26	2		*	*												Ring finger protein 26
MED28	2		*	*											yes	Mediator complex subunit 28
TRIM55	2	*	*													Tripartite motif-containing 55
ANKRD30A	2		*	*										yes		Ankyrin repeat domain 30A
MED19	2	*		*											yes	Mediator complex subunit 19
HMCN2	2	*		*												Hemicentin 2
RGPD8	2		*	*											yes	RANBP2-like and GRIP domain containing 8
RELA	3	*	*	*							*			yes	yes	v-rel reticuloendotheliosis viral oncogene homolog A
MED7	3	*	*	*											yes	Mediator complex subunit 7
MED6	3	*	*	*											yes	Mediator complex subunit 6

The asterisks indicate positive calls. Set names are as in Table 1. “Druggable” is as determined in Hopkins and Groom and Orth et al. [56],[57].

We then asked whether further enrichment relative to the NCBI HIV interaction database was achieved by examining human genes identified in at least two siRNA two screens. Of the 34 genes on two or more lists, 11 were previously reported in the HIV interaction database (NUP153, CCNT1, CTDP1, CHST1, CD4, CXCR4, TCEB3, JAK1, AKT1, DDX3X, and RELA), or 30% of the total, substantially higher than the 13%–18% identified in each single list alone. From this we infer that the newly identified genes called in two or more siRNA screens (Table 3) are more likely to be authentic new cellular cofactors for HIV infection. Twenty-nine out of the 34 genes were found to be expressed in cells or tissues expressing CD4 and coreceptor by transcriptional profiling analysis, and so competent for HIV entry. Of the remaining five, CCNT1 (cyclinT1) is known to be expressed in T cells and represents a false negative call in the expression data used. A comprehensive table of all genes identified in pairwise combinations of lists 1–12 is provided at the end of Report S1 together with the expression analysis (pp. 72–98).

Why did the three different siRNA screens yield such different gene lists? One possible explanation could be that the expression of host cell factors differed between the HeLa and 293T cells studied. However, analysis of transcriptional profiling data showed that >93% of the genes called as important for HIV infection in any one of the three studies were expressed in both cell types.

However, variation due to 1) experimental noise, 2) timing of sampling, and 3) different filtering criteria likely do explain some of the differences. Two replicates were available for analysis from the König et al. screen, allowing estimation of the variance. From this, the expected overlap for of the top 300 genes in replicate screens could be simulated. A test of two replicates or ten replicates per screen (Figure 1C) yielded 150 or 240 overlapping genes, illustrating how the high variance reduced the overlap, but replication improves it.

A second source of variation was differences between time points analyzed, which varied among the published siRNA screens. Although data were not available for multiple time points for the HIV screens, data were available for a screen of influenza virus infection at three time points (S. Chanda, unpublished data). Analysis demonstrated that variation between time points was of the same magnitude as variation within time points and partially independent.

A third source of variation is likely to be differences in the filtering thresholds used. We investigated the effects of different choices for the toxicity filter by reanalyzing the data of the König et al. screen using three different toxicity thresholds. In the first, no filter was applied (100% of genes were accepted for further analysis), in the second, only genes in the 50% least toxic group were considered, in the third, only the 20% least toxic genes were considered. For each set, the 300 genes with the strongest reduction in HIV infection after knockdown were extracted and the overlap among sets compared (Figure 1D). Fewer than 150 genes out of 300 overlapped between the 100% and 20% sets, and the maximum between any pair was 222 genes, indicating that the final gene set called is very sensitive to the toxicity threshold chosen.

Thus variations between replicates, between time points, and in filtering thresholds all likely contributed to the differences between siRNA screens. Further differences also likely arose from use of different siRNA libraries, cell types, and viral strains [5].

**Figure 2:**
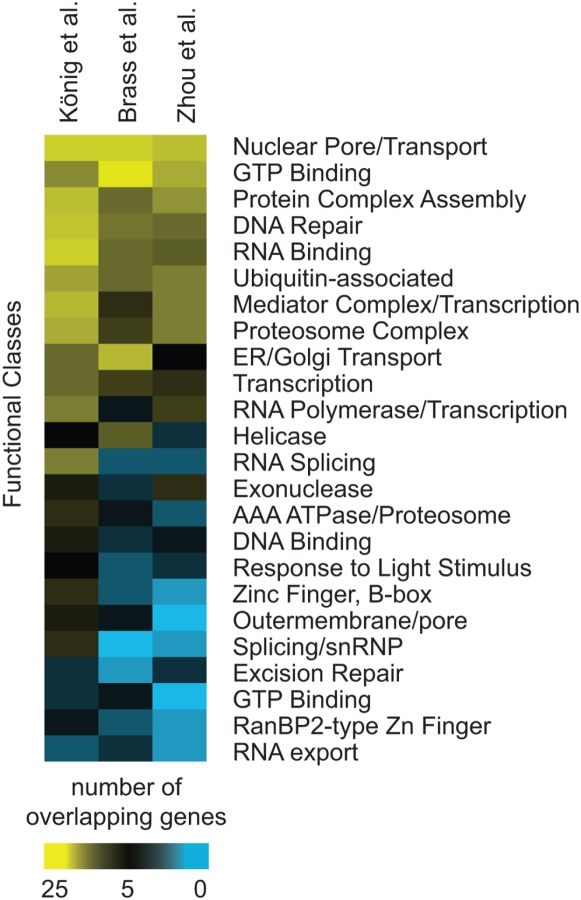
Gene Ontology Analysis of GO Groups Enriched among Genes Called in Two or More siRNA Screens. The color code indicates the number of genes in each functional group from each screen derived using DAVID Functional Annotation Clustering. Annotations for each function group were based on the assessment of GO categories that comprised each group, which can be found in Table S1.

We next asked whether the three siRNA screens yielded host factors participating in similar cellular processes. We extracted overrepresented functional clusters for each screen (lists 1–3) using The Database for Annotation, Visualization, and Integrated Discovery (DAVID) tool [15]. Overrepresented clusters were then filtered for significance (*p*<0.06 based on a geometric mean of all terms in a group), redundancy, biological relevance, and specificity. This yielded 24 functional groups, most containing contributions from factors identified in two or more screens (Figure 2; genes are cataloged in Table S1). For example, all three screens were enriched in factors involved in “Nuclear Pore/Transport” (21–24 genes each), which likely facilitate the trafficking of HIV complexes between cellular compartments, including the nuclear import of the HIV pre-integration complex, export of viral RNAs, or possibly synthesis of other required factors. Other functional classes or complexes identified included “DNA Repair”, “Ubiquitin-associated”, “Mediator Complex/Transcription”, “RNA Binding”, “GTP Binding”, and “Helicase”. Thus, a functional analysis of the three screens revealed greater overlap in gene ontology (GO) categories than was seen for individual genes.

## Recovery of Well-Documented Host Genes Affecting HIV in Genome-Wide Studies

A variety of well-documented cellular cofactors for infection were identified in two out of three screens, including i) the binding and entry factors CD4 and CXCR4; ii) the NFkappaB subunit RELA; iii) the activating kinases AKT1 and JAK1; iv) the Vpr and Vif cofactor TCEB3/elonginB; and v) the Tat cofactor CCNT1/cyclinT1 [16] (which was also in the mass spectrometry study of Tat-associated proteins). The Rev cofactor DDX3X [17], an RNA helicase, was also identified in two out of three screens and in addition was found by mass spectrometry to bind to both Tat and Rev. The DNA repair factor MRE11 was also identified, which was previously implicated in HIV DNA circularization [18], though effects on HIV infection efficiency have not been reported previously.

A variety of further well-established factors were identified in one siRNA screen only. The well-studied viral budding factor TSG101 [19] was called in the Zhou et al. siRNA screen (list 3), and also identified as associated with HIV particles after release. The Rev cofactor XPO1/CRM1 [20] was called in the Zhou et al. siRNA screen but not the others.

Also instructive is analyzing the known HIV cofactors that were not identified. HLA-B57 and HLA-C have well-documented effects on viral set point and HIV disease progression [8],[21], but these were not detected in the siRNA screens, probably because the HLA proteins affect the immune response to HIV and not replication at the cellular level. The integration cofactor PSIP1/LEDGF/p75 was not identified, probably because only very complete knockdowns diminish HIV replication [22]–[30]. Several genes known to encode products important for HIV replication were identified in the initial screen of König et al., which yielded 4,019 candidates, but were not further validated in the filtered data set of 293 proteins. These included Sp1, a transcription factor known to bind the HIV LTR; the HIV Gag binding protein cyclophilin A (PPIA) [31]; and several integrin proteins, believed to assist in virus binding to cells (ITGB1, ITGB2, ITGB3). The ESCRT proteins are known to be important in HIV budding [32], but only VPS24 was identified. Another member of this complex, VPS53, was called in the Brass et al. study, and the initial unfiltered König et al. screen, but not in other studies. The RNA lariat debranching enzyme DBR1 was used as a positive control in the König et al. study, and is well known to affect reverse transcription [33], but DBR1 was not identified in any of the other studies. Thus, the recovery of already implicated host factors was generally good in the overlap analysis, providing confidence about the authenticity of the newly called genes. However, some well-documented factors were missed, indicating that other factors important for HIV replication were probably missed by the analysis.

## Network Analysis of the HIV–Host Interactome

To identify the cellular subsystems recruited by HIV in more detail, we assembled a host–pathogen protein interaction network based on the gene products in lists 1–10 (Table 1). The network interaction map took advantage of binary protein binding relationships cataloged in curated literature-based protein–protein interaction databases (i.e., Bind, HPRD, MINT, Reactome, etc.). HIV–host interactions were predicted based on evidence compiled in the NCBI HIV interaction database. The resulting HIV–host network was comprised of 1,657 cellular proteins that formed interactions with other host cell factors or HIV-encoded proteins (*p*<10^−5^). Two hundred and ninety of these host proteins (“nodes”) were supported by experimental evidence from two or more datasets, reflecting a 35% enrichment of proteins that are called by multiple datasets in this analysis. We performed a further analysis to identify unusually dense network neighborhoods within this interactome map using a graph theoretic clustering algorithm (MCODE) [34]. This revealed 11 putative molecular clusters, ten of which could be associated with distinct biochemical or cellular functions.

### Proteasome

**Figure 3:**
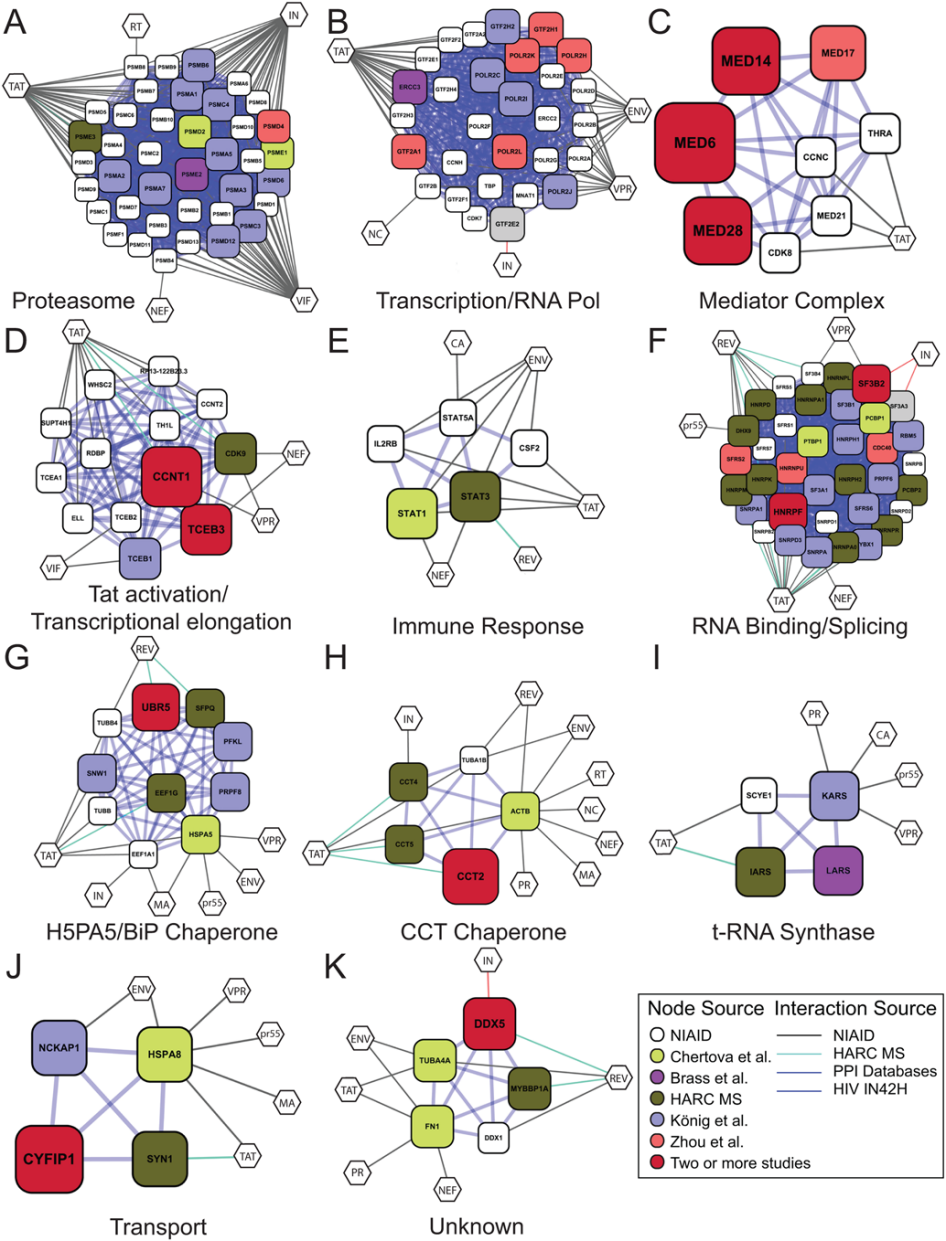
Gene Clusters, Generated Using PPI and MCODE Analysis, Derived from the Full Set of Genes Implicated in HIV Infection (Lists 1–10). The size of each node is proportional to the number of screens in which the host cell gene was called. Gene identifiers are in Table S1. Diamonds indicate genes from the NCBI HIV interactions database. Color code: red = König et al., green = Brass et al., blue = Zhou et al., cyan = Fellay et al., magenta = Frankel interaction screens (unpublished data), yellow = HIV particle associated, and grey = Studamire and Goff integrase interactions. For genes that were called in multiple screens (larger symbols), a color was chosen arbitrarily from among the screens positive for that gene. Default parameters were used, specifically— Degree Cutoff: 2. Node Score Cutoff: 0.0. Haircut: true. Fluff: false. K-Core: 2. Maximum Depth From Seed: 100. (A) Proteasome; (B) Transcription/RNA Pol; (C) Mediator Complex; (D) Tat activation/Transcriptional elongation; (E) Immune response; (F) RNA Binding/Splicing; (G) H5PA5/BiP Chaperone; (H) CCT Chaperone; (I) t-RNA Synthase; (J) Transport; (K) Unknown.

A densely connected network of proteasome subunits was identified by the MCODE analysis (Figure 3A). The proteasome was prominent in the published siRNA screening data, and implicated in probable early steps of viral infection. In previous literature, the proteasome was shown to act negatively on HIV infection by destroying replication intermediates [35],[36]. The siRNA data indicate that the proteasome may also facilitate HIV infection. The mechanism is unclear and could be indirect–for example, reducing proteasome activity may alter cellular ubiquitin levels, and so affect HIV replication by altering the free ubiquitin pool.

### Transcription/RNA Polymerase

Genes for subunits of RNA polymerase II and associated factors were identified in several different screens, yielding a densely connected network (Figure 3B). In some of the siRNA screens, the knockdown of Pol II subunits was mapped to the step of Tat transactivation. Because so many subunits were identified, the simplest interpretation is that reduced dosage of the full complex is responsible for the deficit in HIV replication.

### Mediator Complex

Multiple subunits of the mediator complex were identified in two or more siRNA screens (Table 3 and Figure 3C). The mediator complex links transcriptional activator proteins to the RNA polymerase II basal transcription apparatus, thereby allowing transcriptional activation [37],[38]. The observation that so many subunits were identified suggests that activity of the complex as a whole is the target of siRNA modulation. Viral replication cycle mapping by Zhou et al. indicated that some of the mediator proteins were needed to support Tat-activated transcription, though studies in König et al. suggest a possible further role in reverse transcription. The data can be accommodated in a model where changes in dosage in the mediator complex are not toxic to cells, but where Tat-activated transcription is extremely sensitive to mediator dosage. Previously, mediator was shown to be important for Sp1-driven transcription, and Sp1 is required for transcription from the HIV LTR, suggesting possible involvement of Sp1 as well.

### Tat Activation/Transcriptional Elongation

A dense network was formed containing the Tat cofactor cyclin T1 (CCNT1) [16], which was identified in two out of three siRNA screens and by mass spectrometry (Figure 3D). Together with its binding partner CDK9, which was identified as a Tat binding protein, cyclinT1 forms positive transcription elongation factor b (P-TEFb). The MCODE analysis links the P-TEFb complex and the elongin complex involved in transcriptional elongation. Another factor, the RNA Pol II carboxyl-terminal domain (CTD) phosphatase CTDP1, was also identified and was also previously associated with Tat activation. In addition, two STAT proteins, also involved in transcription and NFkappaB signaling and implicated in lentiviral infection [39], were identified (Figure 3E).

### RNA Binding/Splicing

A large cluster of RNA binding and splicing proteins was identified in the MCODE analysis. Eleven of the cellular genes encode protein components of hnRNP complexes (HNR factors) that form on pre-mRNA and direct splicing and other activities. HNRNPU contains both an RNA binding domain and a DNA binding domain that mediates attachment to the nuclear scaffold, potentially linking sites of mRNA synthesis to specific sub-nuclear locations. Six further genes (SF3 factors) encode components of the splicing factor 3 a/b complex, which is involved in activating the U2 snRNP and promoting splicing. Three SNR proteins and two SF proteins were also identified and are implicated in RNA splicing and RNP formation. Several of these proteins were implicated in the literature to modulate Tat or Rev function (e.g., [40]–[42]), and seven direct binding interactions to these viral proteins were identified in the mass spectrometry data reported here.

Several observations also suggest possible connections of RNAP/splicing factors to the viral DNA integration step. Two components of the splicing factor SF3 bound integrase in the yeast two-hybrid data (list 9) [10]. The splicing protein SNW1/SKIIP1 was found by König et al. be selectively important at the integration step [3]. The integrase-interacting protein PSIP1/LEDGF/p75 appears to tether integrase to active transcription units [23],[29],[43],[44], and an alternatively spliced variant of this protein (p52) is involved in RNA metabolism [45]. Though indirect, these observations suggest a model in which splicing factors may help recruit integrase to active transcription units, which are favored for integration [46]–[48].

Another possible role of splicing factors is in maintaining the proper balance between spliced and incompletely spliced HIV RNAs. HIV replication requires multiply spliced messages (encoding Tat, Rev, and Nef), singly spliced messages (encoding Vif, Vpr, Env/Vpu, and a second form of Tat), and unspliced messages (encoding Gag and Gag-Pol). The unspliced RNA also serves as the genomic RNA. Alterations in dosage of splicing factors by siRNA knockdown may well diminish HIV replication by altering the ratios of the different HIV mRNA forms.

### The BiP/GRP78/HSPA5 Chaperone

The BiP/GRP78/HSPA5 protein chaperone was identified in the network analysis (Figure 3G). BiP/GRP78/HSPA5 is a member of the HSP70 family that is involved in the folding and assembly of proteins in the endoplasmic reticulum. BiP has been implicated in interacting with newly synthesized HIV gp160 SU/TM precursor [49], and HSP70 family members have been proposed to interact with Gag, Tat, Vpr, and MA. The MCODE analysis connected BiP/GRP78/HSPA5 to a collection of nuclear proteins involved in splicing (PRPF8, SFPQ, and SNW1), nuclear matrix architecture (MATR3), and ubiquitylation (UBR5). Determining how these cellular proteins modulate the interactions of BiP/GRP78/HSPA5 with HIV proteins offers a potential route to better understanding protein folding and sorting during HIV replication.

### The CCT Chaperone

The MCODE analysis identified subunits of the chaperone containing TCP1 (CCT) complex (Figure 3H). Subunits were identified in siRNA screens, in HIV particles after budding, and also as Tat binding proteins. This complex consists of two identical stacked rings of eight subunits. Unfolded proteins are thought to pass through the central cavity, and become folded in an ATP-dependent manner. The CCT chaperone has not previously been associated with HIV replication, and represents a new candidate for involvement in Tat activation and HIV budding.

### Additional Densely Connected Clusters

Several further functions were identified, including proteins involved in t-RNA synthase function, transport, and one of unknown function (Figure 3 I–3K). The t-RNA synthase and transport complexes contained members that associated with Tat according to the mass spectrometry study, and the unknown complex contained a member binding to Rev, suggesting specific links to HIV replication.

### Other Newly Identified Functions

Several sets of proteins were identified that were not called as densely connected networks but appear to be functionally related. The nuclear pore and associated factors were clustered in the initial MCODE network but were not sufficiently densely to emerge as a densely connected network. Proteins involved in nuclear import identified in two out of three siRNA screens included products of NUP153, RANBP2, TNPO3, and RGPD8. NUP153 and TNPO3 have been associated with the trafficking of HIV proteins previously [2], [3], [50]–[52]. RANBP2 is a giant gene encoding a product that accumulates at nuclear pores and binds to RAN, which is a small GTP-binding protein of the RAS superfamily. RANBP2 also contains FG repeats, a cyclophilin-related nucleoporin, and a domain that binds UBC9, the E2 for SUMO1 transfer. RGPD8 is named for “RANBP2-like and GRIP domain containing 8”. It too accumulates at the nuclear pore and is believed to assist in RNA and protein transport. The actions of NUP153 and TNPO3 have been mapped to nuclear import of the HIV preintegration complex in [2],[3],[51], and NUP153 has also been proposed to be involved in export of HIV Rev [53].

Three genes were identified that affect the microtubule system. MAP4 is a microtubule-associated protein that has not previously been studied in detail. MID1IP1 is a regulator of microtubule polymerization. CAV2 (caveolin 2) is involved in the formation of plasma membrane invaginations involved in a variety of cellular functions including signal transduction, cell growth, and apoptosis. Caveoli have also been implicated as interacting with the microtubule network [54]. Previous studies have suggested that HIV particles may traffic along microtubules to reach the nucleus [55]. Thus MAP4, MID1IP1, and possibly CAV2 are candidates for cofactors in this process. Other proteins were also called in two siRNA screens (ANAPC2, DMXL1, HMCN2, and IDH1) but are of unknown function (Table 3).

## Identifying New Drug Targets

One of the main reasons for carrying out the screens for host factors is the hope of identifying new targets for HIV therapeutics. Several studies have indentified potentially “druggable” human proteins by cataloging families of InterPro domains where one member is the target of one or more small molecule inhibitors with drug-like properties. All members of the family are then proposed as potential drug targets [56],[57] (John Hogenesch, data available at http://www1.qiagen.com/Products/GeneSilencing/LibrarySiRna/SiRnaSets/HumanDruggableGenomesiRNASetV30.aspx?ShowInfo=1). In Report S1 (pp. 72–120), we annotate our overlap study for “druggable” targets by these criteria. Focusing on an updated version of the list from Hopkins and Groom [56], we found that eight of the 34 genes common to the two siRNA screens were called as potential drug targets (Table 3, column labeled “druggable”). Two of these eight are in fact known to be the targets of small molecules with activity against HIV. CXCR4 is the target of AMD3100 and related molecules [58],[59], and AKT1 is the target of miltefosine [60]. The fact that two out of eight genes called as druggable are known antiviral drug targets (at least in tissue culture) suggests that this analysis is yielding viable new targets. Of the additional genes encoding candidate drug targets, inhibitors have been reported for the kinases ADRBK1/GRK2 and JAK1. These can be tested for activity against HIV in cell culture. Annotation of the larger collection of genes that were found on two or more lists (lists 1–9) yielded a further 56 genes encoding potentially druggable cellular factors.

## Summary

Analysis of genes called as important for HIV replication in multiple genome-wide screens yielded a list rich in well-known factors and also intriguing new candidates. Many important factors were surely missed by this approach, but at least some of the most promising new genes can be distilled from among the 9.5% of all human protein-coding genes now proposed to affect HIV infection. Many of the new genes can be linked into clusters, specifying cellular subsystems associated with HIV replication. Promising drug targets could be discerned among the best-documented new factors.

## Methods

Overlap analysis and comparisons to random distributions were carried out using R [61]. The *p*-values for overlaps between lists were generated by comparison to results of random simulation and by calculation based on the hypergeometric distribution as in [14]. No correction was applied for multiple comparisons. Networks were generated using MCODE analysis on the binary interaction file and plotted in cytoscape. The global interaction network was judged to be statistically significant by comparison to random simulation (*p*<10^−5^). Subnetworks were selected that 1) contained at least two proteins from different studies and 2) showed high connectivity. The protein–protein interaction data for Nef, Tat, and Rev from the HARC Center were derived using previously described methods (LC MS-MS followed by database matching) [62]. A more thorough analysis of these (early stage) data will be reported elsewhere.

Updated versions of Report S1 documenting the overlap among genome-wide screens can be found at http://www.hostpathogen.org.

## Supporting Information

Report S1 Gene List Comparison Report
(1.88 MB PDF)

Table S1 Gene IDs from Functional Clusters in Figure 3
(0.09 MB XLS)

## References

[1] Rines DR, Tu B, Miraglia L, Welch GL, Zhang J (2006). High-content screening of functional genomic libraries.. Methods Enzymol.

[2] Brass AL, Dykxhoorn DM, Benita Y, Yan N, Engelman A (2008). Identification of host proteins required for HIV infection through a functional genomic screen.. Science.

[3] Konig R, Zhou Y, Elleder D, Diamond TL, Bonamy GM (2008). Global analysis of host-pathogen interactions that regulate early-stage HIV-1 replication.. Cell.

[4] Zhou H, Xu M, Huang Q, Gates AT, Zhang XD (2008). Genome-scale RNAi screen for host factors required for HIV replication.. Cell Host Microbe.

[5] Goff SP (2008). Knockdown screens to knockout HIV-1.. Cell.

[6] Hutvagner G, Zamore PD (2002). RNAi: Nature abhors a double-strand.. Curr Opin Genet Dev.

[7] Konig R, Chiang CY, Tu BP, Yan SF, DeJesus PD (2007). A probability-based approach for the analysis of large-scale RNAi screens.. Nat Methods.

[8] Fellay J, Shianna KV, Ge D, Colombo S, Ledergerber B (2007). A whole-genome association study of major determinants for host control of HIV-1.. Science.

[9] Chertova E, Chertov O, Coren LV, Roser JD, Trubey CM (2006). Proteomic and biochemical analysis of purified human immunodeficiency virus type 1 produced from infected monocyte-derived macrophages.. J Virol.

[10] Studamire B, Goff SP (2008). Host proteins interacting with the moloney murine leukemia virus integrase: Multiple transcriptional regulators and chromatin binding factors.. Retrovirology.

[11] Fu W, Sanders-Beer BE, Katz KS, Maglott DR, Pruitt KD (2009). Human immunodeficiency virus type 1, human protein interaction database at NCBI.. Nucleic Acids Res.

[12] Hao L, Sakurai A, Watanabe T, Sorensen E, Nidom CA (2008). Drosophila RNAi screen identifies host genes important for influenza virus replication.. Nature.

[13] Krishnan MN, Ng A, Sukumaran B, Gilfoy FD, Uchil PD (2008). RNA interference screen for human genes associated with west nile virus infection.. Nature.

[14] Fury W, Batliwalla F, Gregersen PK, Li W (2006). Overlapping probabilities of top ranking gene lists, hypergeometric distribution, and stringency of gene selection criterion.. Conf Proc IEEE Eng Med Biol Soc.

[15] Dennis G, Sherman BT, Hosack DA, Yang J, Gao W (2003). Database for annotation, visualization, and integrated discovery.. Genome Bio.

[16] Wei P, Garber ME, Fang SM, Fischer WH, Jones KA (1998). A novel CDK9-associated C-type cyclin interacts directly with HIV-1 tat and mediates its high-affinity, loop-specific binding to TAR RNA.. Cell.

[17] Yedavalli VS, Neuveut C, Chi YH, Kleiman L, Jeang KT (2004). Requirement of DDX3 DEAD box RNA helicase for HIV-1 rev-RRE export function.. Cell.

[18] Kilzer JM, Stracker TH, Beitzel B, Meek K, Weitzman MD (2003). Roles of host cell factors in circularization of retroviral DNA.. Virology.

[19] Garrus JE, von Schwedler UK, Pornillos OW, Morham SG, Zavitz KH (2001). Tsg101 and the vacuolar protein sorting pathway are essential for hiv-1 budding.. Cell.

[20] Neville M, Stutz F, Lee L, Davis LI, Rosbash M (1997). The importin-beta family member Crm1p bridges the interaction between rev and the nuclear pore complex during nuclear export.. Curr Biol.

[21] Matthews PC, Prendergast A, Leslie A, Crawford H, Payne R (2008). Central role of reverting mutations in HLA associations with human immunodeficiency virus set point.. J Virol.

[22] Llano M, Saenz DT, Meehan A, Wongthida P, Peretz M (2006). An essential role for LEDGF/p75 in HIV integration.. Science.

[23] Marshall H, Ronen K, Berry C, Llano M, Sutherland H (2007). Role of PSIP1/LEDGF/p75 in lentiviral infectivity and integration targeting.. PLoS ONE.

[24] Ciuffi A, Diamond T, Hwang Y, Marshall H, Bushman FD (2006). Fusions of LEDGF/p75 to lambda repressor promote HIV DNA integration near lambda operators in vitro.. Hum Gene Ther.

[25] Llano M, Vanegas M, Fregoso O, Saenz D, Chung S (2004). LEDGF/p75 determines cellular trafficking of diverse lentiviral but not murine oncoretroviral integrase proteins and is a component of functional lentiviral preintegration complexes.. J Virol.

[26] Cherepanov P (2007). LEDGF/p75 interacts with divergent lentiviral integrases and modulates their enzymatic activity in vitro.. Nucleic Acids Res.

[27] Cherepanov P, Maertens G, Proost P, Devreese B, Van Beeumen J (2003). HIV-1 integrase forms stable tetramers and associates with LEDGF/p75 protein in human cells.. J Biol Chem.

[28] Maertens G, Cherepanov P, Pluymers W, Busschots K, De Clercq E (2003). LEDGF/p75 is essential for nuclear and chromosomal targeting of HIV-1 integrase in human cells.. J Biol Chem.

[29] Ciuffi A, Llano M, Poeschla E, Hoffmann C, Leipzig J (2005). A role for LEDGF/p75 in targeting HIV DNA integration.. Nat Med.

[30] Turlure F, Devroe E, Silver PA, Engelman A (2004). Human cell proteins and human immunodeficiency virus DNA integration.. Front Biosci.

[31] Gamble TR, Vajdos FF, Yoo S, Worthylake DK, Houseweart M (1996). Crystal structure of human cyclophillin A bound to the amino-terminal domain of HIV-1 capsid.. Cell.

[32] von Schwedler UK, Stuchell M, Muller B, Ward DM, Chung HY (2003). The protein network of HIV budding.. Cell.

[33] Ye Y, De Leon J, Yokoyama N, Naidu Y, Camerini D (2005). DBR1 siRNA inhibition of HIV-1 replication.. Retrovirology.

[34] Bader GD, Hogue CW (2003). An automated method for finding molecular complexes in large protein interaction networks.. BMC Bioinformatics.

[35] Schwartz O, V M, Friguet B, Arenzana-Seisdedos F, Heard J (1998). Antiviral activity of the proteasome on incoming human immunodeficiency virus type 1.. J Virol.

[36] Butler SL, Johnson EP, Bushman FD (2002). HIV cDNA metabolism studied by fluorescence-monitored PCR: Notable stability of two-LTR circles.. J Virol.

[37] Kuras L, Borggrefe T, Kornberg RD (2003). Association of the mediator complex with enhancers of active genes.. Proc Natl Acad Sci U S A.

[38] Kornberg RD (2007). The molecular basis of eukaryotic transcription.. Proc Natl Acad Sci U S A.

[39] Mohan M, Aye PP, Borda JT, Alvarez XJ, Lackner AA (2007). Gastrointestinal disease in SIV-infected rhesus macaques is characterized by proinflammatory dysregulation of the IL-6-JAK-STAT3 pathway.. Am J Pathol.

[40] Pongoski J, Asai K, Cochrane A (2002). Positive and negative modulation of human immunodeficiency virus type 1 rev function by cis and trans regulators of viral RNA splicing.. J Virol.

[41] Roy BB, Hu J, Guo X, Russell RS, Guo F (2006). Association of RNA helicase a with human immunodeficiency virus type 1 particles.. J Biol Chem.

[42] Li J, Tang H, Mullen TM, Westberg C, Reddy TR (1999). A role for RNA helicase A in post-transcriptional regulation of HIV type 1.. Proc Natl Acad Sci U S A.

[43] Shun MC, Raghavendra NK, Vandegraaff N, Daigle JE, Hughes S (2007). LEDGF/p75 functions downstream from preintegration complex formation to effect gene-specific HIV-1 integration.. Genes Dev.

[44] Ciuffi A, Diamond TL, Hwang Y, Marshall HM, Bushman FD (2006). Modulating target site selection during human immunodeficiency virus DNA integration in vitro with an engineered tethering factor.. Hum Gene Ther.

[45] Ge H, Si Y, Roeder RG (1998). Isolation of cDNAs encoding novel transcription coactivators p52 and p75 reveals an alternate regulatory mechanism of transcriptional activation.. EMBO J.

[46] Schroder AR, Shinn P, Chen H, Berry C, Ecker JR (2002). HIV-1 integration in the human genome favors active genes and local hotspots.. Cell.

[47] Mitchell RS, Beitzel BF, Schroder AR, Shinn P, Chen H (2004). Retroviral DNA integration: ASLV, HIV, and MLV show distinct target site preferences.. PLoS Biol.

[48] Wu X, Li Y, Crise B, Burgess SM (2003). Transcription start regions in the human genome are favored targets for MLV integration.. Science.

[49] Earl PL, Moss B, Doms RW (1991). Folding, interaction with GRP78-BiP, assembly, and transport of the human immunodeficiency virus type 1 envelope protein.. J Virol.

[50] Luban J (2008). HIV-1 infection: Going nuclear with TNPO3/Transportin-SR2 and integrase.. Curr Biol.

[51] Christ F, Thys W, De Rijck J, Gijsbers R, Albanese A (2008). Transportin-SR2 imports HIV into the nucleus.. Curr Biol.

[52] Varadarajan P, Mahalingam S, Liu P, Ng SB, Gandotra S (2005). The functionally conserved nucleoporins Nup124p from fission yeast and the human Nup153 mediate nuclear import and activity of the Tf1 retrotransposon and HIV-1 vpr.. Mol Biol Cell.

[53] Zolotukhin AS, Felber BK (1999). Nucleoporins nup98 and nup214 participate in nuclear export of human immunodeficiency virus type 1 rev.. J Virol.

[54] Ito J, Kheirollah A, Nagayasu Y, Lu R, Kato K (2006). Apolipoprotein A-I increases association of cytosolic cholesterol and caveolin-1 with microtubule cytoskeletons in rat astrocytes.. J Neurochem.

[55] McDonald D, Vodicka MA, Lucero G, Svitkina TM, Borisy GG (2002). Visualization of the intracellular behavior of HIV in living cells.. J Cell Biol.

[56] Hopkins AL, Groom CR (2002). The druggable genome.. Nat Rev Drug Discov.

[57] Orth AP, Batalov S, Perrone M, Chanda SK (2004). The promise of genomics to identify novel therapeutic targets.. Expert Opin Ther Targets.

[58] Schols D, Este JA, Henson G, De Clercq E (1997). Bicyclams, a class of potent anti-HIV agents, are targeted at the HIV coreceptor fusin/CXCR-4.. Antiviral Res.

[59] Harrison JE, Lynch JB, Sierra LJ, Blackburn LA, Ray N (2008). Baseline resistance of primary HIV-1 strains to the CXCR4 inhibitor AMD3100.. J Virol.

[60] Chugh P, Bradel-Tretheway B, Monteiro-Filho CM, Planelles V, Maggirwar SB (2008). Akt inhibitors as an HIV-1 infected macrophage-specific anti-viral therapy.. Retrovirology.

[61] R Development Core Team (2006). R: A language and environment for statistical computing.. R Foundation for Statistical Computing.

[62] Krogan NJ, Cagney G, Yu H, Zhong G, Guo X (2006). Global landscape of protein complexes in the yeast saccharomyces cerevisiae.. Nature.

