# Supplementary material for: Host Cell Factors in HIV Replication: Meta-Analysis of Genome-Wide Studies
Source: PLoS Pathog. 2009 May 29;5(5):e1000437. doi: 10.1371/journal.ppat.1000437 (PMC2682202; doi:10.1371/journal.ppat.1000437)
Supplement: Report S1 — Gene List Comparison Report (1.88 MB PDF) [file ppat.1000437.s001.pdf]

Gene List Comparison Report

April 27, 2009

Contents

1 Introduction: How to use this report **2**

1.1 Gene lists studied in this report. 3

2 Analysis of pairwise overlap between genome-wide studies **5**

2.1 siRNA HIV König(293) vs. siRNA HIV Brass(283) 5
2.2 siRNA HIV König(293) vs. siRNA HIV Zhou(303) 6
2.3 siRNA HIV König(293) vs. SNP HIV Fellay(63) 7
2.4 siRNA HIV König(293) vs. Particle Associated HIV(248) 8
2.5 siRNA HIV König(293) vs. HARC Nef(6) 9
2.6 siRNA HIV König(293) vs. HARC Tat(69) 10
2.7 siRNA HIV König(293) vs. HARC Rev(56) 11
2.8 siRNA HIV König(293) vs. BIND HIV IN(23) 12
2.9 siRNA HIV König(293) vs. NCBI Interactions(1434) 13
2.10 siRNA HIV König(293) vs. siRNA Flu Fly(98) 14
2.11 siRNA HIV König(293) vs. siRNA WNV(305) 15
2.12 siRNA HIV Brass(283) vs. siRNA HIV Zhou(303) 16
2.13 siRNA HIV Brass(283) vs. SNP HIV Fellay(63) 17
2.14 siRNA HIV Brass(283) vs. Particle Associated HIV(248) 18
2.15 siRNA HIV Brass(283) vs. HARC Nef(6) 19
2.16 siRNA HIV Brass(283) vs. HARC Tat(69) 20
2.17 siRNA HIV Brass(283) vs. HARC Rev(56) 21
2.18 siRNA HIV Brass(283) vs. BIND HIV IN(23) 22
2.19 siRNA HIV Brass(283) vs. NCBI Interactions(1434) 23
2.20 siRNA HIV Brass(283) vs. siRNA Flu Fly(98) 24
2.21 siRNA HIV Brass(283) vs. siRNA WNV(305) 25
2.22 siRNA HIV Zhou(303) vs. SNP HIV Fellay(63) 26
2.23 siRNA HIV Zhou(303) vs. Particle Associated HIV(248) 27
2.24 siRNA HIV Zhou(303) vs. HARC Nef(6) 28
2.25 siRNA HIV Zhou(303) vs. HARC Tat(69) 29
2.26 siRNA HIV Zhou(303) vs. HARC Rev(56) 30
2.27 siRNA HIV Zhou(303) vs. BIND HIV IN(23) 31
2.28 siRNA HIV Zhou(303) vs. NCBI Interactions(1434) 32
2.29 siRNA HIV Zhou(303) vs. siRNA Flu Fly(98) 33
2.30 siRNA HIV Zhou(303) vs. siRNA WNV(305) 34
2.31 SNP HIV Fellay(63) vs. Particle Associated HIV(248) 35
2.32 SNP HIV Fellay(63) vs. HARC Nef(6) 36
2.33 SNP HIV Fellay(63) vs. HARC Tat(69) 37
2.34 SNP HIV Fellay(63) vs. HARC Rev(56) 38
2.35 SNP HIV Fellay(63) vs. BIND HIV IN(23) 39
2.36 SNP HIV Fellay(63) vs. NCBI Interactions(1434) 40
2.37 SNP HIV Fellay(63) vs. siRNA Flu Fly(98) 41

2.38 SNP HIV Fellay(63) vs. siRNA WNV(305) . . . 42
2.39 Particle Associated HIV(248) vs. HARC Nef(6) . . . 43
2.40 Particle Associated HIV(248) vs. HARC Tat(69) . . . 44
2.41 Particle Associated HIV(248) vs. HARC Rev(56) . . . 45
2.42 Particle Associated HIV(248) vs. BIND HIV IN(23) . . . 46
2.43 Particle Associated HIV(248) vs. NCBI Interactions(1434) . . . 47
2.44 Particle Associated HIV(248) vs. siRNA Flu Fly(98) . . . 48
2.45 Particle Associated HIV(248) vs. siRNA WNV(305) . . . 49
2.46 HARC Nef(6) vs. HARC Tat(69) . . . 50
2.47 HARC Nef(6) vs. HARC Rev(56) . . . 51
2.48 HARC Nef(6) vs. BIND HIV IN(23) . . . 52
2.49 HARC Nef(6) vs. NCBI Interactions(1434) . . . 53
2.50 HARC Nef(6) vs. siRNA Flu Fly(98) . . . 54
2.51 HARC Nef(6) vs. siRNA WNV(305) . . . 55
2.52 HARC Tat(69) vs. HARC Rev(56) . . . 56
2.53 HARC Tat(69) vs. BIND HIV IN(23) . . . 57
2.54 HARC Tat(69) vs. NCBI Interactions(1434) . . . 58
2.55 HARC Tat(69) vs. siRNA Flu Fly(98) . . . 59
2.56 HARC Tat(69) vs. siRNA WNV(305) . . . 60
2.57 HARC Rev(56) vs. BIND HIV IN(23) . . . 61
2.58 HARC Rev(56) vs. NCBI Interactions(1434) . . . 62
2.59 HARC Rev(56) vs. siRNA Flu Fly(98) . . . 63
2.60 HARC Rev(56) vs. siRNA WNV(305) . . . 64
2.61 BIND HIV IN(23) vs. NCBI Interactions(1434) . . . 65
2.62 BIND HIV IN(23) vs. siRNA Flu Fly(98) . . . 66
2.63 BIND HIV IN(23) vs. siRNA WNV(305) . . . 67
2.64 NCBI Interactions(1434) vs. siRNA Flu Fly(98) . . . 68
2.65 NCBI Interactions(1434) vs. siRNA WNV(305) . . . 69
2.66 siRNA Flu Fly(98) vs. siRNA WNV(305) . . . 70

3 Table of genes called in two or more studies 72

4 Table of union of gene from siRNA screens, annotated for "Druggability" and expression in CD4-positive cells 98

5 Table of genes that appear in two or more siRNA screens 120

6 Analysis of all Genome-wide screens versus the NCBI interactions list 122

7 Table summarizing all pairwise overlaps between screens 124

8 References 127

---

1 Introduction: How to use this report

This report analyzes the overlap among gene sets identified as important for replication of HIV or other viruses. The report consists of five main sections. The first (p. 3-5) describes the gene lists used. The second (p. 5-91) shows the significance of the overlap between each pair of lists, and the genes common to both. The third section (p. 72-98) presents a table of genes called in two or more screens, summarizing the set of screens in which each was called. The fourth section (p. 98-120) presents annotation for all genes called in the siRNA screens, and their "druggability". The fifth section (p.121-122) shows only the genes called in two or more siRNA screens. The last three pages present some additional summary statistics on overlap. If a reader is interested in a particular gene, simply searching on the gene name calls up the relevant analysis.

The lists were obtained from the original authors or their publications and housed in a MySQL database. Gene calls were updated to use GeneID names for comparison. The background lists of all genes sampled in each study were similarly compiled. Overlaps between lists were determined, and their significance assessed using random simulations (1000 random draws from each background list) or calculation of hypergeometric p-values as in Fury et al. (PMID: 17947148).

Table 1: Lists used in the report.

List	Name	Number of genes	Description	Reference
1	siRNA HIV König	293	siRNA screen for host factor promoting HIV replication	PMID: 18854154
2	siRNA HIV Brass	283	siRNA screen for host factor promoting HIV replication	PMID: 18187620
3	siRNA HIV Zhou	303	siRNA hits from the HIV host factor screen from Merck	PMID: 18976975
4	SNP HIV Fellay	63	GWA for HIV set point in infected individuals	PMID: 17641165
5	Particle Associated HIV	248	proteins in HIV particles identified by mass spec	PMID: 16940516
6	HARC Nef	6	Gene products that interact with HIV Nef protein (mass spec)	this work
7	HARC Tat	69	Gene products that interact with HIV Tat protein (mass spec)	this work
8	HARC Rev	56	Gene products that interact with HIV Rev protein (mass spec)	this work
9	BIND HIV IN	23	Integrase interacting proteins from a yeast two hybrid screen	PMID: 18554410
10	NCBI Interactions	1434	Published interactions between an HIV protein and a cellular protien	PMID: 18927109
11	siRNA Flu Fly	98	Human homologs of fly gene products important for influenza virus infection	PMID: 18615016
12	siRNA WNV	305	Gene products important for West Nile virus infection	PMID: 18690214

1.1 Gene lists studied in this report.

1. **siRNA screen for human genes affecting HIV infection from König et al. (1)**. König et al investigated the requirements for 20,000 human genes during HIV infection, using six siRNAs to knock down each gene in human 293T cells. The cells were then infected with an HIV vector encoding luciferase, allowing infection to be monitored by quantifying luciferase activity. For experimental convenience, the VSV glycoprotein, and not the HIV envelope protein, was used for infection, so that genes involved in HIV entry were not queried in this experiment (though genes involved in VSV-G entry were analyzed). Similarly, genes important in the late steps of HIV replication following viral gene expression were not assayed. A counter screen was carried out to monitor toxicity, and genes that were notably toxic were excluded. A total of 4019 genes were identified as positives that knocked down HIV infection and showed at most modest toxicity. Human genes were further prioritized based on expression in cells hosting HIV replication and participation in gene ontology (GO) or protein networks associated with HIV replication. The additional datasets that were interrogated included: the yeast

2 hybrid protein-protein interaction database (Hynet); the NCBI HIV-1 protein interaction database; and microarray data to correlate expression with the CD4 receptor and either the CXCR4 or CCR5 coreceptors. These genes were then tested further, demanding confirmation by at least two independent siRNAs, yielding a filtered list of 293 genes. Quantitative PCR analysis of viral DNA was used to map the function of these genes to specific stages of the HIV replication cycle (2, 3). It is important to note that since this study focused on confirmation of genes enriched in one or more of these criteria, they are more likely to be appear in subsequent analyses of expression, functional over-representation, or protein interactions that are used here.

2. **siRNA screen from Brass et al. (4)**. List 2 contains 283 genes identified from an siRNA screen reported by Brass et al. About 20,000 human genes were silenced using siRNA and the effects on HIV infection quantified. This study required evidence from only a single siRNA for a positive call, so this aspect of the data is more tentative than the König et al. study, but the Brass et al. study had several advantages as well. Brass et al. used HeLa cells engineered to encode human CD4 and CXCR4, so that infection could be carried out with viruses containing the native HIV envelope protein, thus querying the possible role of additional genes during binding and entry. Brass et al. also arranged their study to query the late steps of HIV replication by transferring supernatants from siRNA-transfected cells to new cells and measuring subsequent infection.

3. **A genome-wide siRNA screen reported by Zhou et al. (5)**. In this study, Zhou et al. infected HeLa cells with HIV and scored infection after 48 hours and 96 hours. The experiment was arranged so that the earlier time point queried predominantly early replication steps, while the later time point queried all replication steps. Factors important only at the later time point can thus be inferred to act at a late stage in the HIV replication cycle. All initial hits were filtered by tests with additional siRNAs, by removal of notably toxic siRNAs, and removal of genes expressed only at very low levels in T-cells. A total of 303 host factors were identified. A specific additional screen was carried out to identify siRNAs affecting Tat-mediated transcription. This study also queried the activities of microRNAs and identified nine that influence HIV replication.

4. **Genes near single nucleotide polymorphisms associated with altered rates of HIV disease progression (6)**. Fellay et al. carried out a genome-wide association study of genetic markers associated with viral set point at steady state (following the acute phase of infection). The steady-state level of virus in blood is predictive of the rate of disease progression. Fellay et al. found three genes that achieved statistical significance after correction for multiple comparisons, and a further 63 that were significant before but not after the correction. These genes are candidates for affecting HIV replication in infected individuals rather than cultured cells, so only a partial overlap is expected with the genes in Lists 1-3. For example, genes important for immune responses against HIV would be detected by the Fellay et al. study but not the siRNA screens.

5. **Genes encoding proteins identified in HIV particles using mass-spectrometry (7)**. In this study, Chertova et al. infected monocyte-derived macrophages with HIV, harvested particles shed into the culture medium, then carried out liquid chromatography-linked tandem mass spectrometry to identify host cell proteins incorporated into the HIV particles. This yielded 248 proteins. Some of these proteins may be functionally significant in the late stages of HIV replication, since cellular proteins bound to viral assembly intermediates are occasionally carried into particles during budding (e. g. TSG101 and AIP1/Alix)(8, 9). Other host proteins may be recruited in order to facilitate entry into new cells (e. g. ICAM) (10, 11). Host cell proteins are also likely to be recruited into particles simply due to their proximity to sites of budding. HIV is proposed to bud from lipid rafts, membrane regions containing distinctive collections of membrane proteins, and some of the are raft proteins are enriched in particles (12-14).

6. **Mass spec analysis of human proteins binding to Nef**, which yielded 19 bound proteins.

7. **Mass spec analysis of human proteins binding to Tat**, which yielded 108 bound proteins.

8. **Mass spec analysis of human proteins binding to Rev**, which yielded 108 bound proteins. For some of the analysis, the mass spectrometry data was dereplicated over lists 6 - 8.

9. **Two-hybrid analysis of integrase binding proteins**. Studamire and Goff reported systematic studies of integrase binding proteins using the yeast two hybrid system, yielding 23 that bind selectively to HIV integrase (15). The study was carried out using mouse proteins as "bait" in the interaction study, so the mouse genes were converted to their human orthologs for the comparison reported here.

10. **Genes proposed in the literature to interact with HIV, from the NCBI database**. The list contains 1434 genes proposed in published literature to interact with HIV proteins or affect HIV replication in some fashion. The type of interaction is also listed, aiding in interpreting the mechanism of action of the gene product. The depth of the literature for each gene call is quite variable–some genes are well established to be central to HIV biology by many papers from multiple laboratories, while other proposed genes are only weakly supported and may not be correctly called. Nevertheless, comparison of this gene list to others indicates how well each method has succeeded in recovering human genes reported to be linked to HIV biology.

11. **Genes implicated in influenza virus replication in insect cells. Hao et al**. used RNAi in Drosophila melanogaster cells to identify host cell genes important for influenza virus infection (16). Use of insect cells has the advantage that long siRNAs can be used to obtain stronger levels of mRNA knockdown, though a disadvantage is that inferences about viral growth in humans are less direct and require additional experiments with human cells. Fly genes identified as being important in this screen were used to identify 98 human homologs.

12. **Genes implicated in West Nile virus replication in human cells. Krishnan et al**. used siRNA in human HeLa cells to identify human genes influencing replication of West Nile virus (a flavivirus) (17). They identified a total of 305 genes. These were also tested against a second flavivirus (Dengue), yielding a core set of human genes important for flavivirus replication.

---

2 Analysis of pairwise overlap between genome-wide studies

2.1 siRNA HIV König(293) vs. siRNA HIV Brass(283)

Total number of Genes overlapping: 13
Overlapping Genes: NUP153, MED7, MED14, CTDP1, TRIM55, RELA, RANBP2, MID1IP1, MAP4, IDH1, TNPO3, DMXL1, MED6,
Backgrounds Used:

```
  Name                        Size
1 "Y_ChandasiRNABackground"  "19023"
2 "Y_moch_ElledgeBackground" "20515"
```

Hypergeometric p-value: <0.001
Simulation p-value:
Number of counts that had equal to or greater overlap than (13) in 1000 permutations: 0 => p-value: <0.001

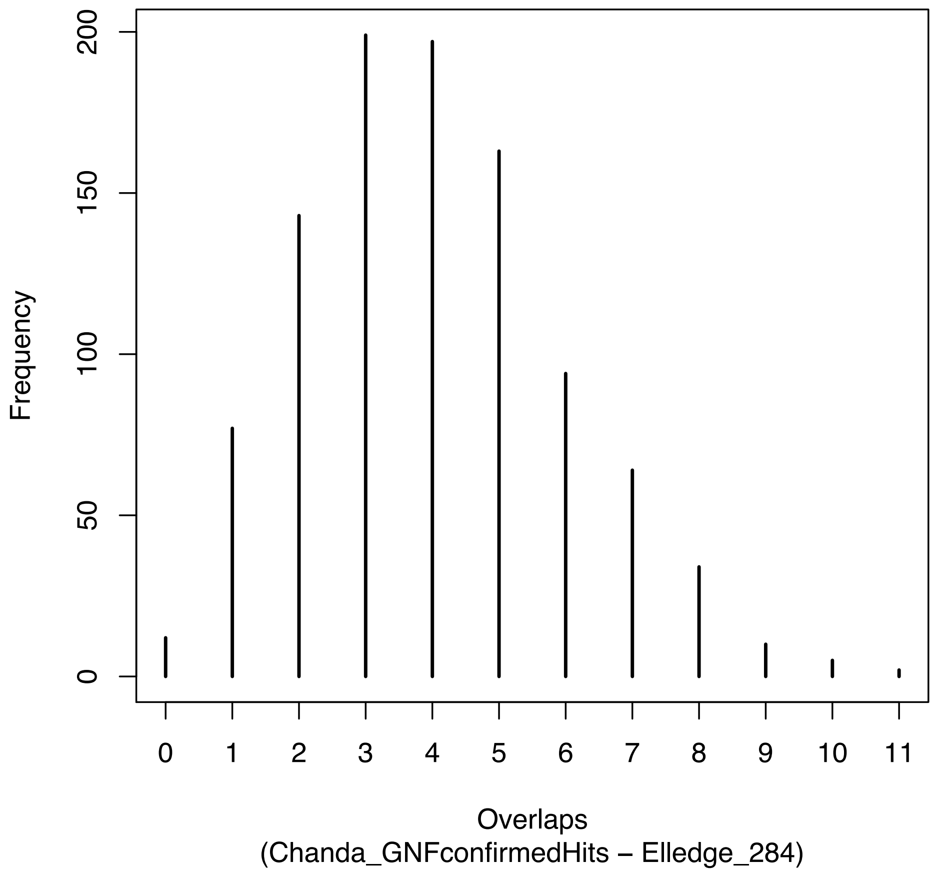

2.2 siRNA HIV König(293) vs. siRNA HIV Zhou(303)

Total number of Genes overlapping: 9
Overlapping Genes: MED7, CHST1, RELA, MRE11A, ANAPC2, HMCN2, MED19, ADRBK1, MED6,
Backgrounds Used:

```
  Name                        Size
1 "Y_ChandasiRNABackground" "19023"
2 "MercksiRNA_Background"   "16450"
```

Hypergeometric p-value: 0.01441603
Simulation p-value:
Number of counts that had equal to or greater overlap than (9) in 1000 permutations: 24 => p-value: 0.024

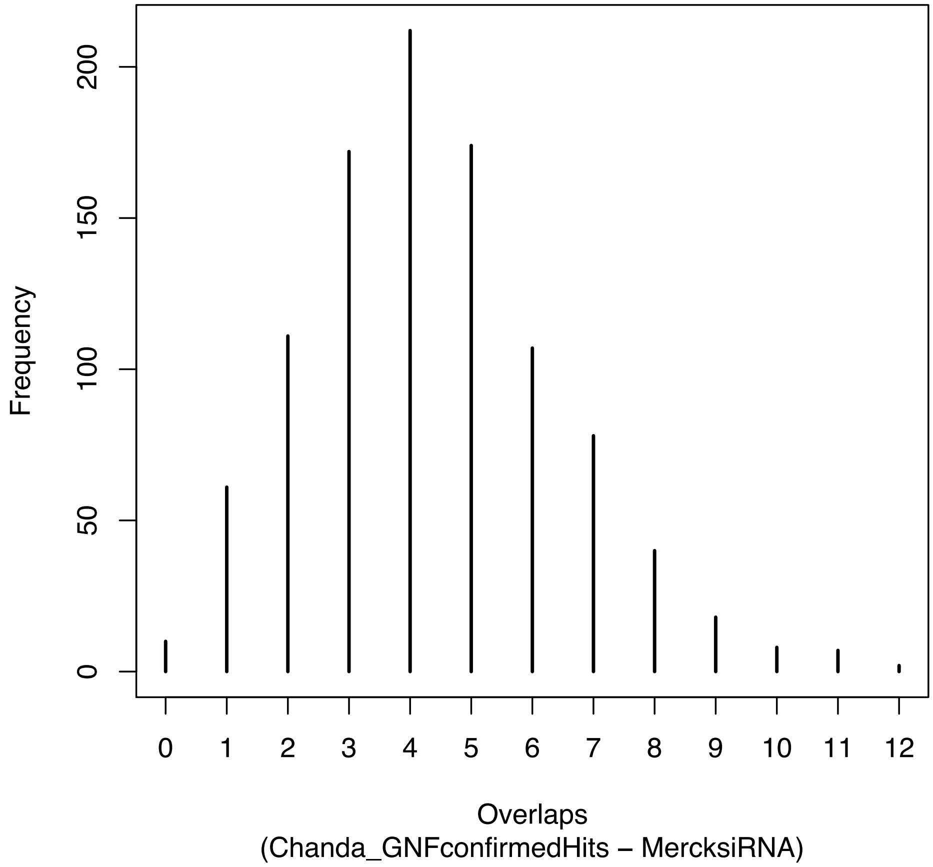

2.3 siRNA HIV König(293) vs. SNP HIV Fellay(63)

Total number of Genes overlapping: 0
Overlapping Genes: ,
Backgrounds Used:

```
  Name                        Size
1 "Y_ChandasiRNABackground" "19023"
2 "Y_UngarBackground"       "22495"
```

Hypergeometric p-value: 1
Simulation p-value:
Number of counts that had equal to or greater overlap than (0) in 1000 permutations: 1000 => p-value: 1

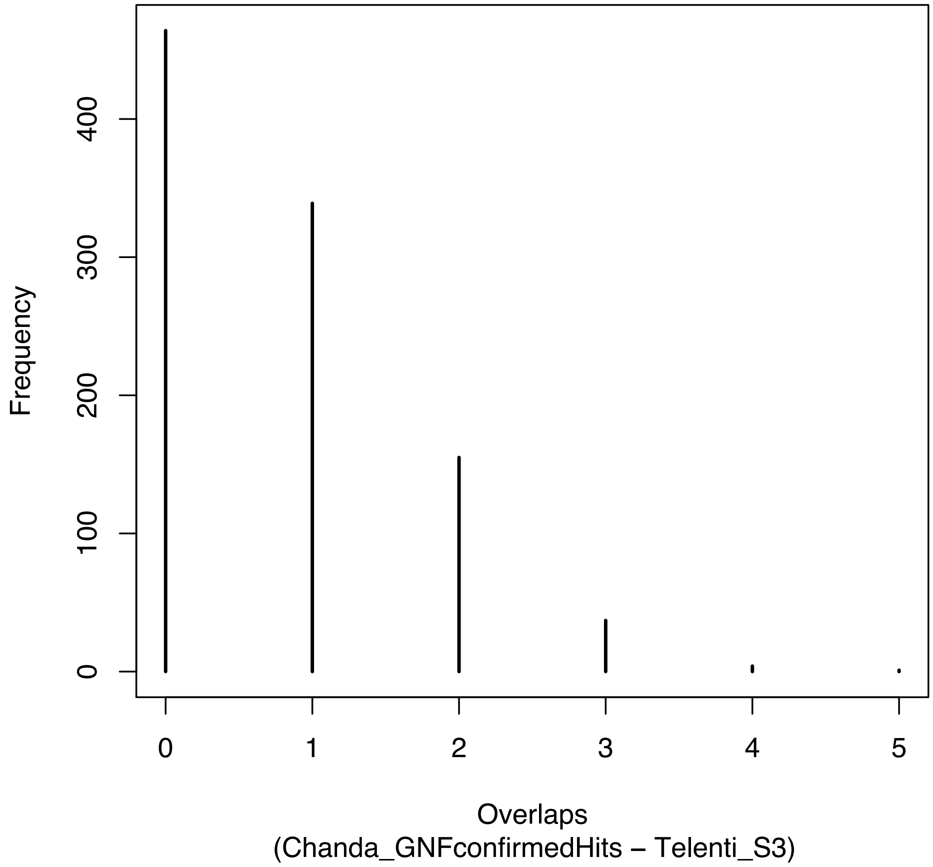

2.4 siRNA HIV König(293) vs. Particle Associated HIV(248)

Total number of Genes overlapping: 5
Overlapping Genes: PTPRJ, KPNB1, FER1L3, CYBB, CLTA,
Backgrounds Used:

	Name	Size
1	"Y_ChandasiRNABackground"	"19023"
2	"NCBI_EntrezProtGenes"	"25157"

Hypergeometric p-value: 0.06380556
Simulation p-value:
Number of counts that had equal to or greater overlap than (5) in 1000 permutations: 154 => p-value: 0.154

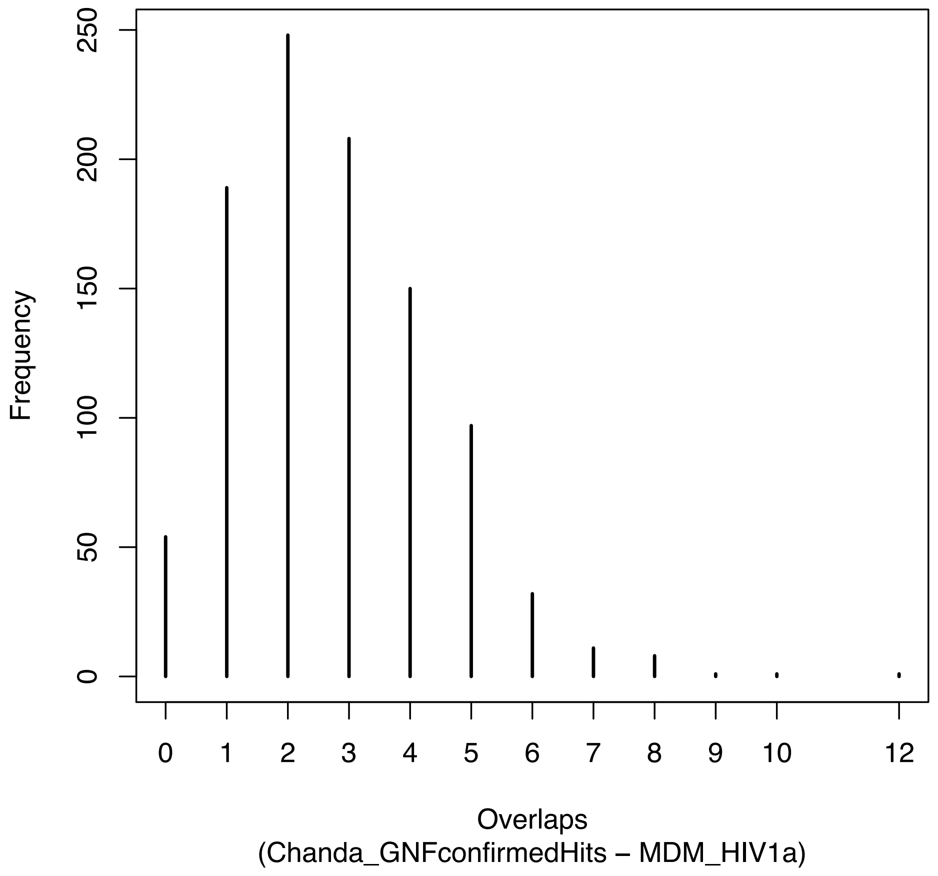

2.5 siRNA HIV König(293) vs. HARC Nef(6)

Total number of Genes overlapping: 0
Overlapping Genes: ,
Backgrounds Used:

```
  Name                        Size
1 "Y_ChandasiRNABackground"  "19023"
2 "NCBI_EntrezProtGenes"     "25157"
```

Hypergeometric p-value: 1
Simulation p-value:
Number of counts that had equal to or greater overlap than (0) in 1000 permutations: 1000 => p-value: 1

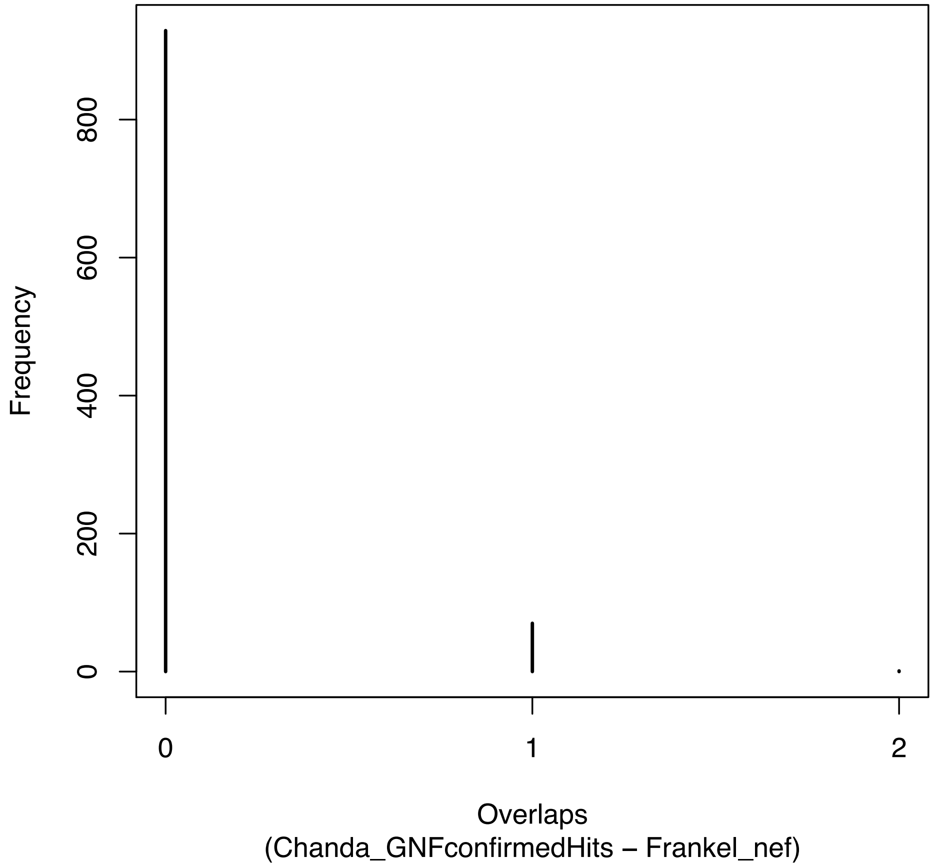

2.6 siRNA HIV König(293) vs. HARC Tat(69)

Total number of Genes overlapping: 0
Overlapping Genes: ,
Backgrounds Used:

```
  Name                       Size
1 "Y_ChandasiRNABackground" "19023"
2 "NCBI_EntrezProtGenes"    "25157"
```

Hypergeometric p-value: 1
Simulation p-value:
Number of counts that had equal to or greater overlap than (0) in 1000 permutations: 1000 => p-value: 1

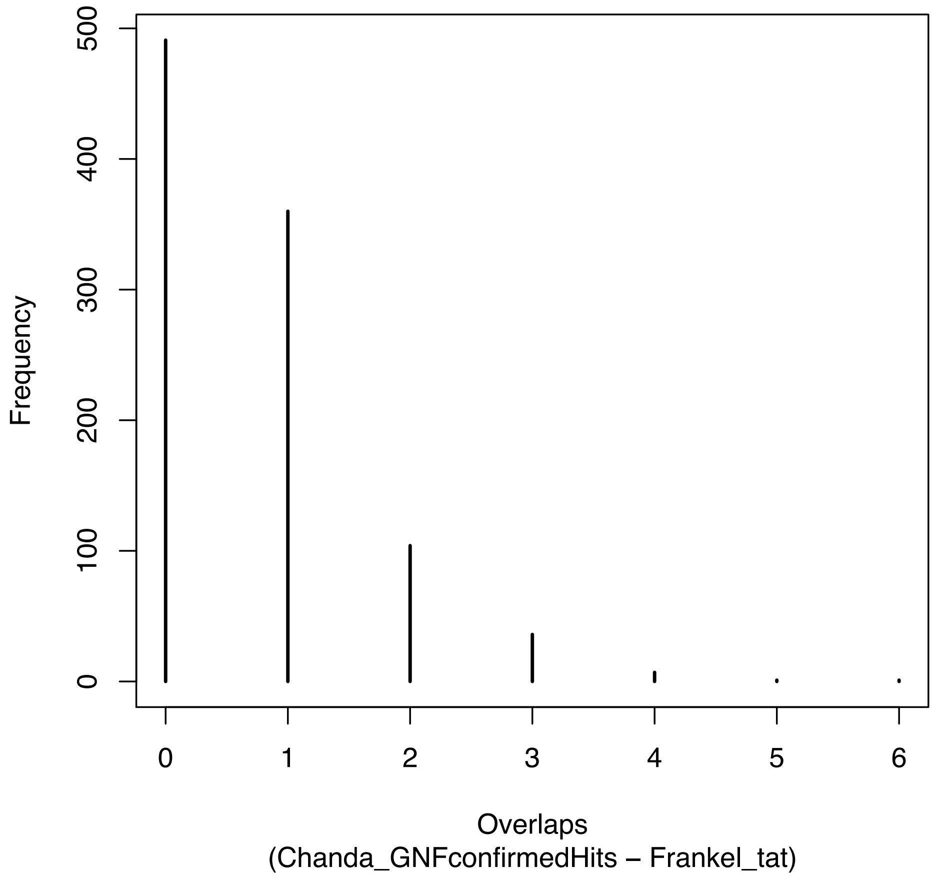

2.7 siRNA HIV König(293) vs. HARC Rev(56)

Total number of Genes overlapping: 2
Overlapping Genes: TAGLN2, DHX15,
Backgrounds Used:

```
  Name                        Size
1 "Y_ChandasiRNABackground" "19023"
2 "NCBI_EntrezProtGenes"    "25157"
```

Hypergeometric p-value: 0.02563252
Simulation p-value:
Number of counts that had equal to or greater overlap than (2) in 1000 permutations: 125 => p-value: 0.125

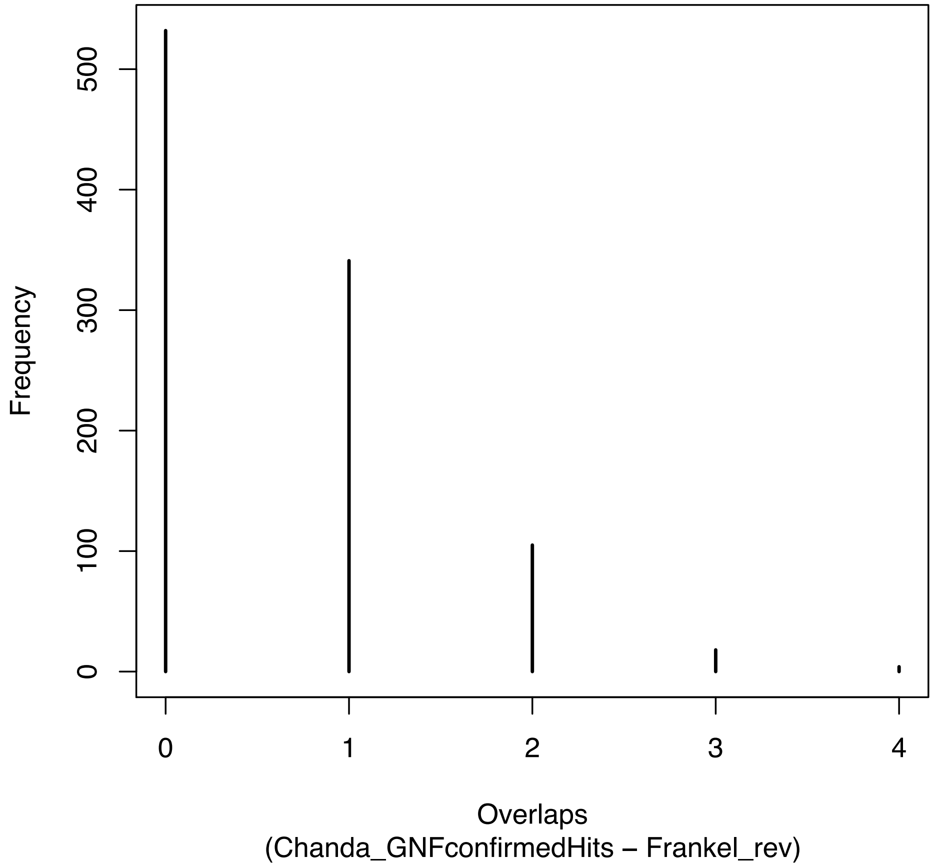

2.8 siRNA HIV König(293) vs. BIND HIV IN(23)

Total number of Genes overlapping: 3
Overlapping Genes: KIF3A, SF3B2, SLU7,
Backgrounds Used:

```
  Name                        Size
1 "Y_ChandasiRNABackground" "19023"
2 "NCBI_EntrezProtGenes"    "25157"
```

Hypergeometric p-value: <0.001
Simulation p-value:
Number of counts that had equal to or greater overlap than (3) in 1000 permutations: 0 => p-value: <0.001

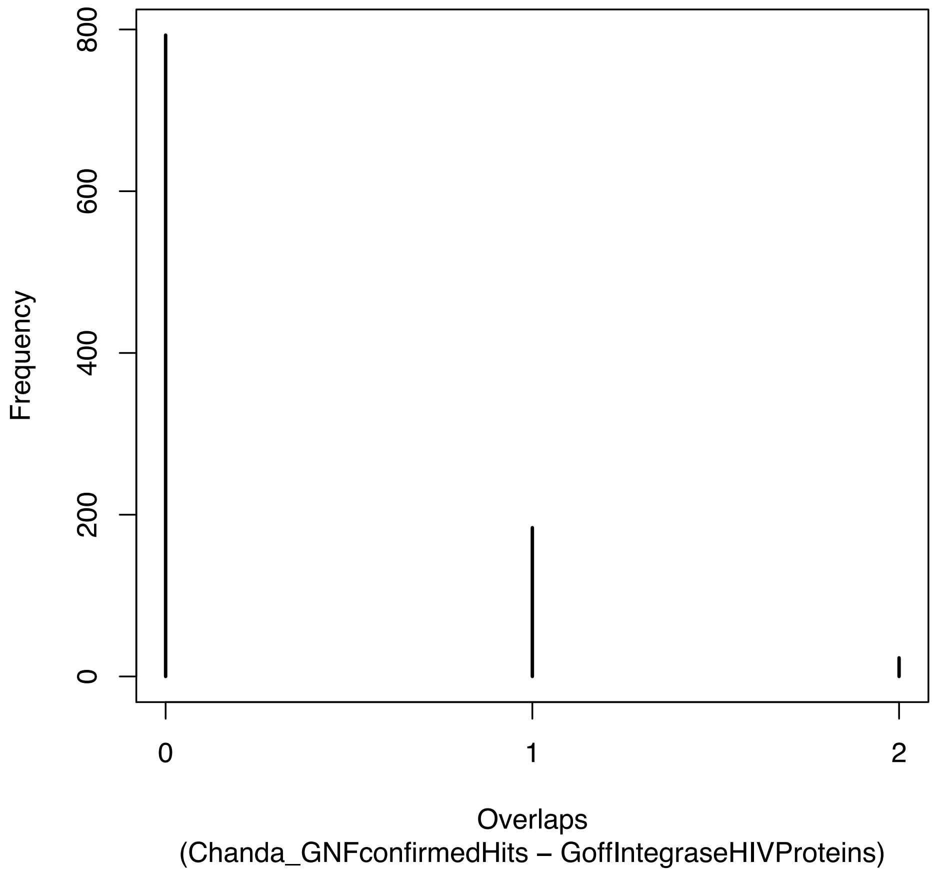

2.9 siRNA HIV König(293) vs. NCBI Interactions(1434)

Total number of Genes overlapping: 54
Overlapping Genes: NUP153, PSMD6, CTDP1, AP1G2, CHST1, NUP214, MYST3, UBE2L3, TCEB1, SNRPD3, SNRPA1, SNRPA, BMP1, ST3GAL3, RELA, PSMD12, PSMC5, PSMC4, PSMC3, PSMB6, PSMA7, PSMA5, PSMA3, PSMA2, PSMA1, PRKCH, PPP2R5E, RNF216, POLR2J, POLR2I, POLR2C, NUP98, YBX1, NEDD4, MT2A, MT1X, POLR2A, MANBA, MAN1A1, KPNB1, KARS, HDAC1, GTF2H2, NUP62, GANAB, SNW1, NLRP1, EP300, CYBB, SF3B2, SUB1, NCKAP1, PTGES3, KHDRBS1,
Backgrounds Used:

```
  Name                        Size
1 "Y_ChandasiRNABackground" "19023"
2 "NCBI_EntrezProtGenes"    "25157"
```

Hypergeometric p-value: <0.001
Simulation p-value:
Number of counts that had equal to or greater overlap than (54) in 1000 permutations: 0 => p-value: <0.001

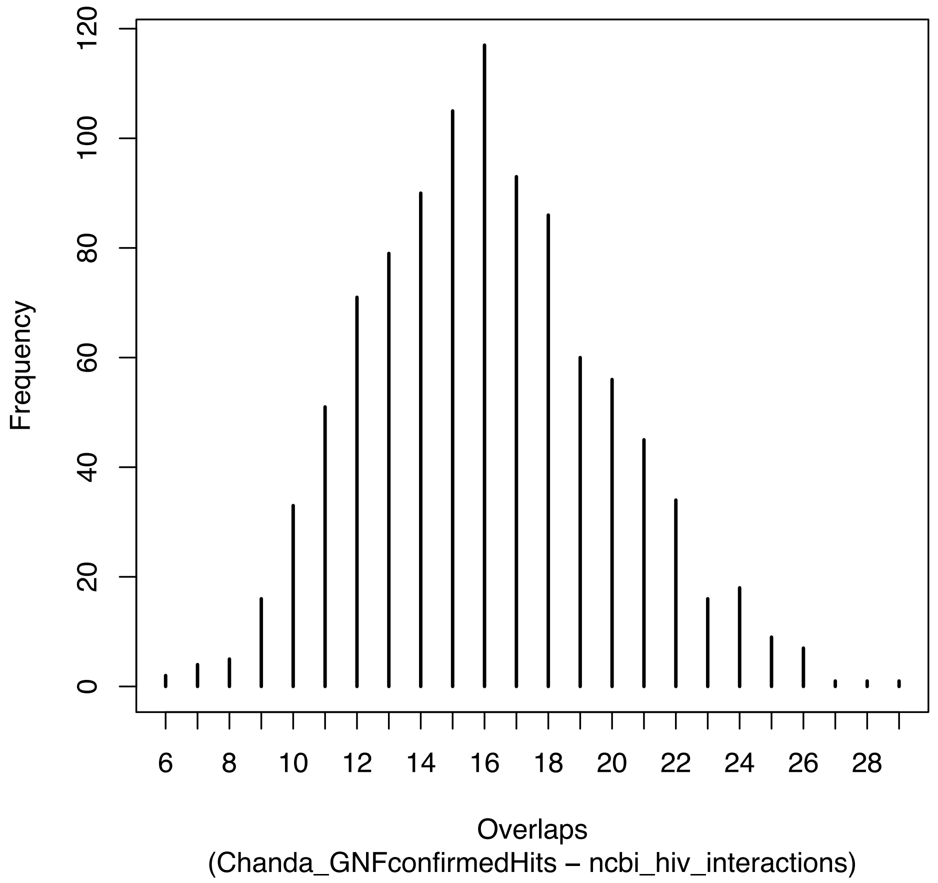

2.10 siRNA HIV König(293) vs. siRNA Flu Fly(98)

Total number of Genes overlapping: 13
Overlapping Genes: NUP153, PSMD6, AQR, SNRPC, PSMD12, PSMC3, PSMB6, ATP6V0C, NUP98, MAT2A, DCP2, RNPS1, NXF1,
Backgrounds Used:

```
  Name                        Size
1 "Y_ChandasiRNABackground" "19023"
2 "Fly_RNAi_background"     "19950"
```

Hypergeometric p-value: <0.001
Simulation p-value:
Number of counts that had equal to or greater overlap than (13) in 1000 permutations: 0 => p-value: <0.001

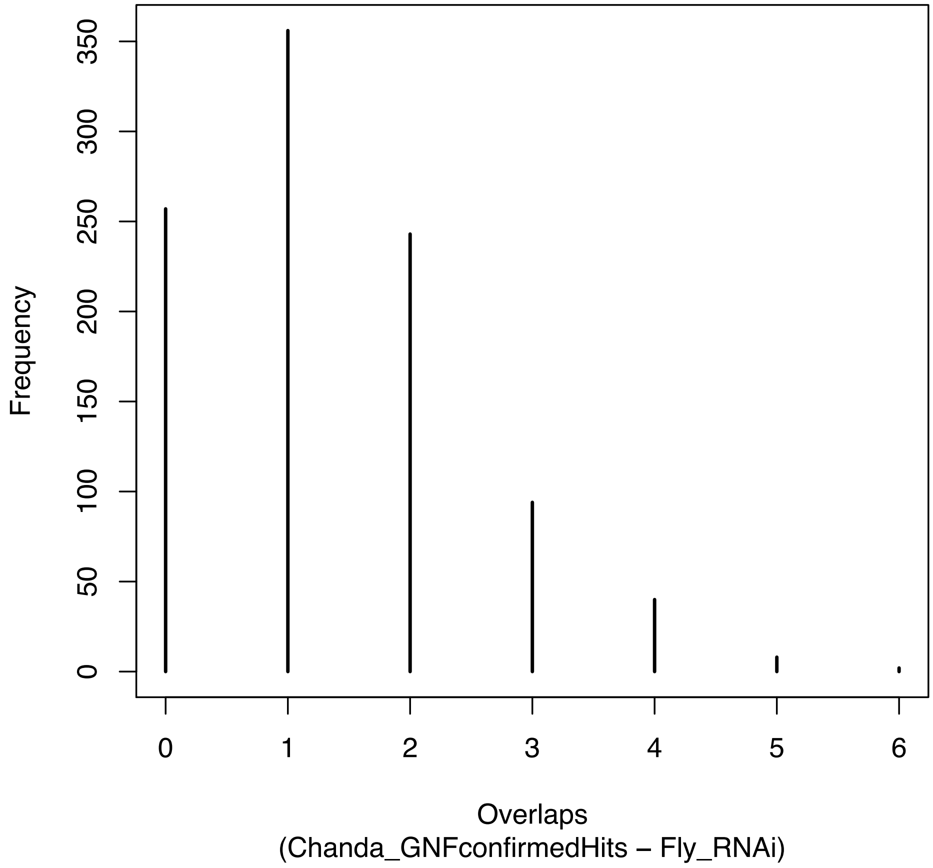

2.11 siRNA HIV König(293) vs. siRNA WNV(305)

Total number of Genes overlapping: 8
Overlapping Genes: NUMBL, NDFIP1, MID1IP1, XAB2, ATP6V0C, MT2A, DHX15, RBM5,
Backgrounds Used:

```
  Name                        Size
1 "Y_ChandasiRNABackground" "19023"
2 "NCBI_EntrezProtGenes"    "25157"
```

Hypergeometric p-value: 0.008299496
Simulation p-value:
Number of counts that had equal to or greater overlap than (8) in 1000 permutations: 20 => p-value: 0.02

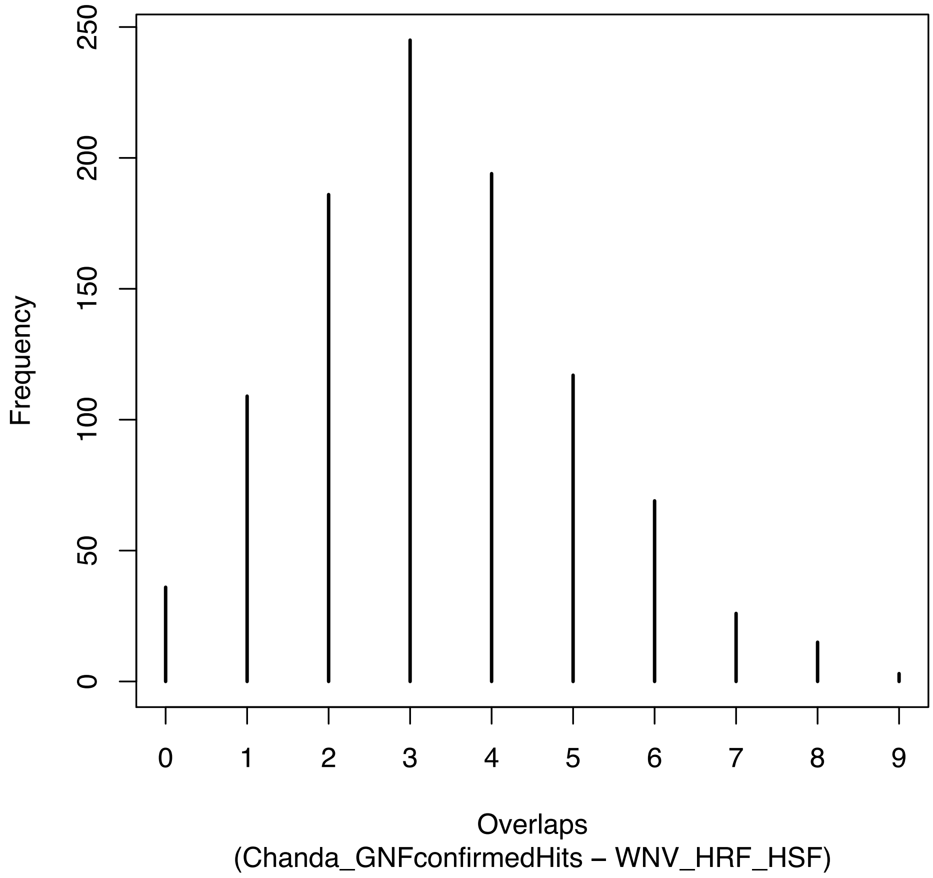

2.12 siRNA HIV Brass(283) vs. siRNA HIV Zhou(303)

Total number of Genes overlapping: 18
Overlapping Genes: MED7, RAB28, CD4, ANKRD30A, CCNT1, CAV2, MED28, RNF26, CXCR4, RGPD8, TCEB3, WNK1, RELA, JAK1, MED4, AKT1, DDX3X, MED6,
Backgrounds Used:

```
  Name                        Size
1 "Y_moch_ElledgeBackground" "20515"
2 "MercksiRNA_Background"    "16450"
```

Hypergeometric p-value: <0.001
Simulation p-value:
Number of counts that had equal to or greater overlap than (18) in 1000 permutations: 0 => p-value: <0.001

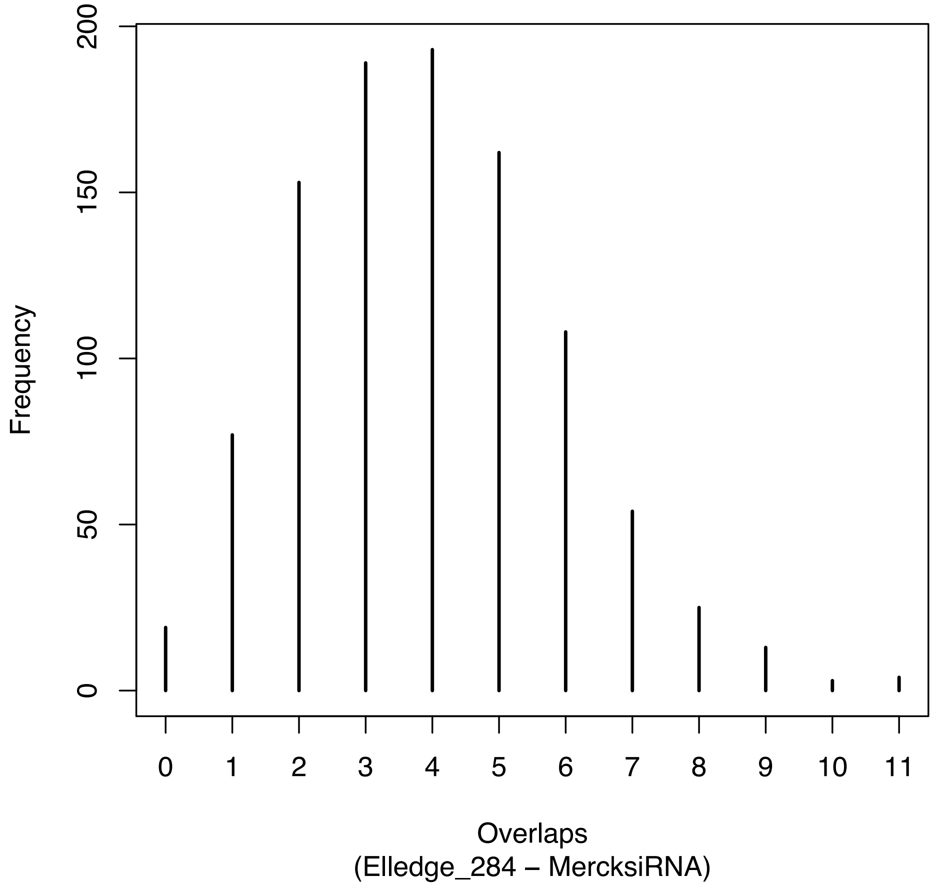

2.13 siRNA HIV Brass(283) vs. SNP HIV Fellay(63)

Total number of Genes overlapping: 1
Overlapping Genes: ZNRD1,
Backgrounds Used:

```
  Name                         Size
1 "Y_moch_ElledgeBackground" "20515"
2 "Y_UngarBackground"        "22495"
```

Hypergeometric p-value: 0.1670130
Simulation p-value:
Number of counts that had equal to or greater overlap than (1) in 1000 permutations: 511 => p-value: 0.511

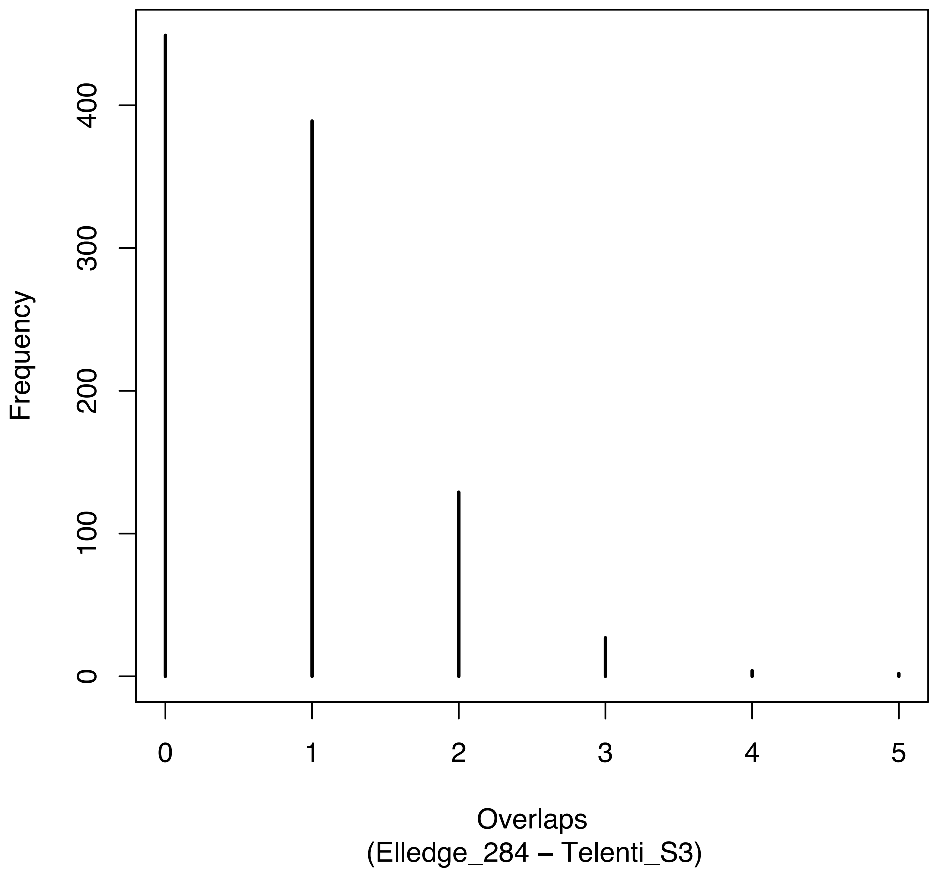

2.14 siRNA HIV Brass(283) vs. Particle Associated HIV(248)

Total number of Genes overlapping: 6
Overlapping Genes: RAP1B, PURA, PPIB, ATP6V0A1, MGAT1, ARF1,
Backgrounds Used:

```
  Name                         Size
1 "Y_moch_ElledgeBackground" "20515"
2 "NCBI_EntrezProtGenes"     "25157"
```

Hypergeometric p-value: 0.01508731
Simulation p-value:
Number of counts that had equal to or greater overlap than (6) in 1000 permutations: 35 => p-value: 0.035

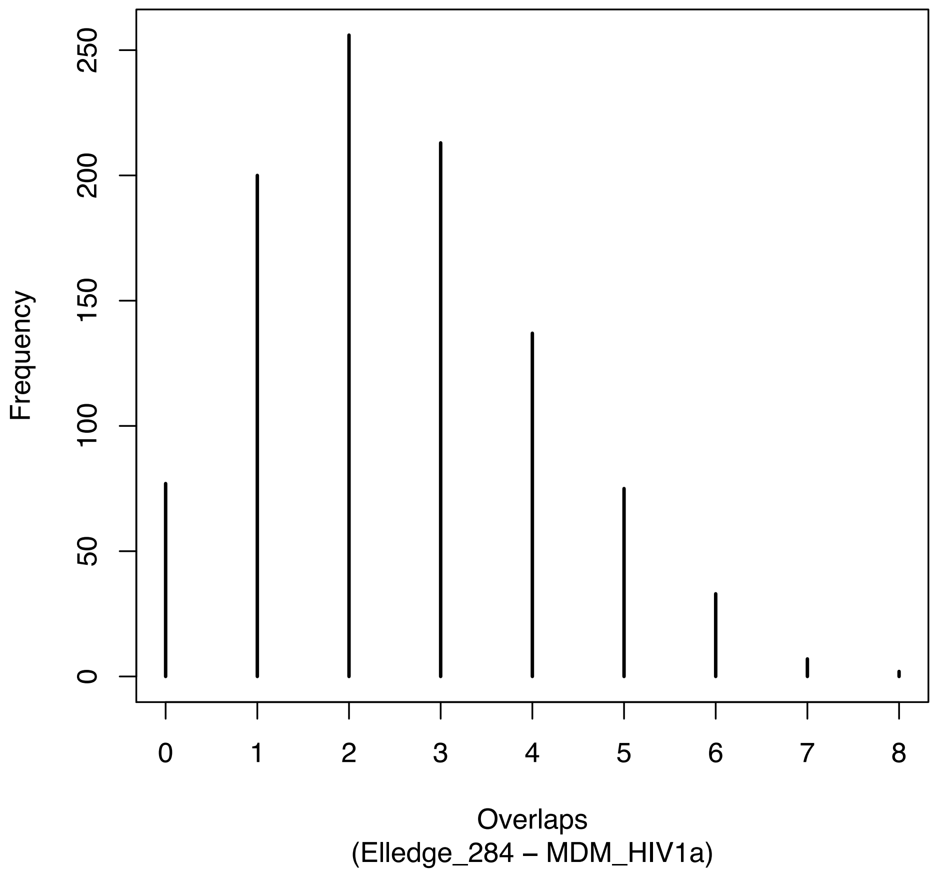

2.15 siRNA HIV Brass(283) vs. HARC Nef(6)

Total number of Genes overlapping: 0
Overlapping Genes: ,
Backgrounds Used:

```
  Name                        Size
1 "Y_moch_ElledgeBackground" "20515"
2 "NCBI_EntrezProtGenes"     "25157"
```

Hypergeometric p-value: 1
Simulation p-value:
Number of counts that had equal to or greater overlap than (0) in 1000 permutations: 1000 => p-value: 1

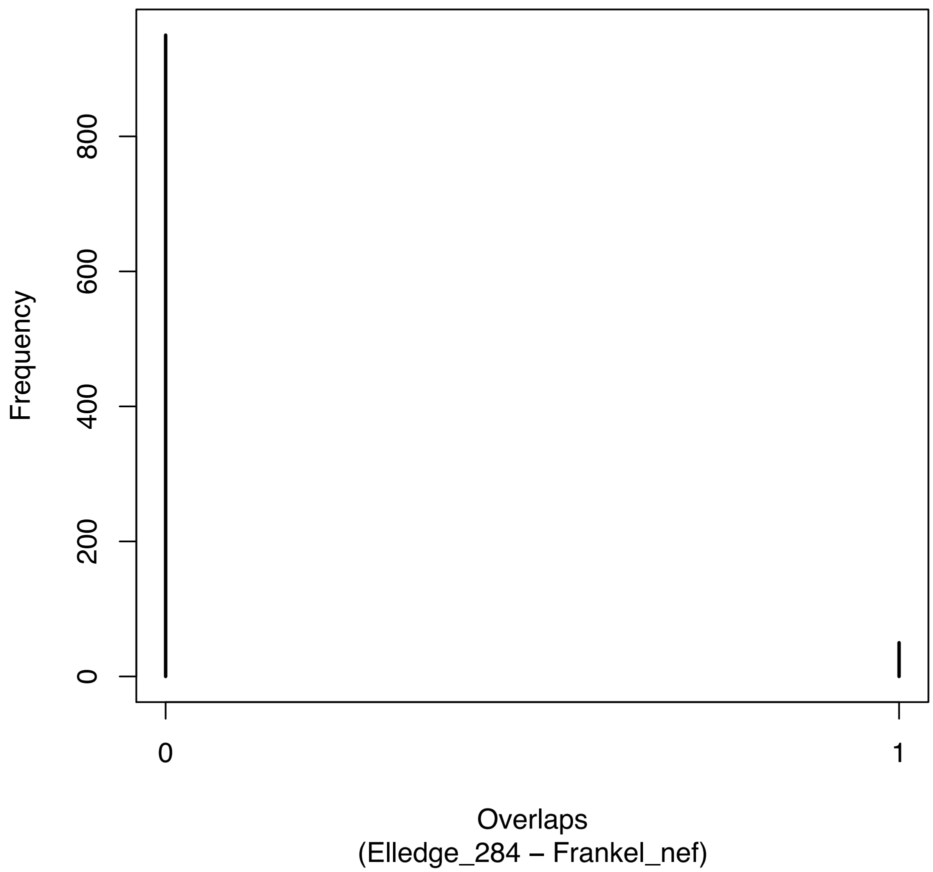

2.16 siRNA HIV Brass(283) vs. HARC Tat(69)

Total number of Genes overlapping: 5
Overlapping Genes: CCNT1, SPTBN1, HNRNPF, DDX3X, CLNS1A,
Backgrounds Used:

```
  Name                        Size
1 "Y_moch_ElledgeBackground" "20515"
2 "NCBI_EntrezProtGenes"     "25157"
```

Hypergeometric p-value: <0.001
Simulation p-value:
Number of counts that had equal to or greater overlap than (5) in 1000 permutations: 4 => p-value: 0.004

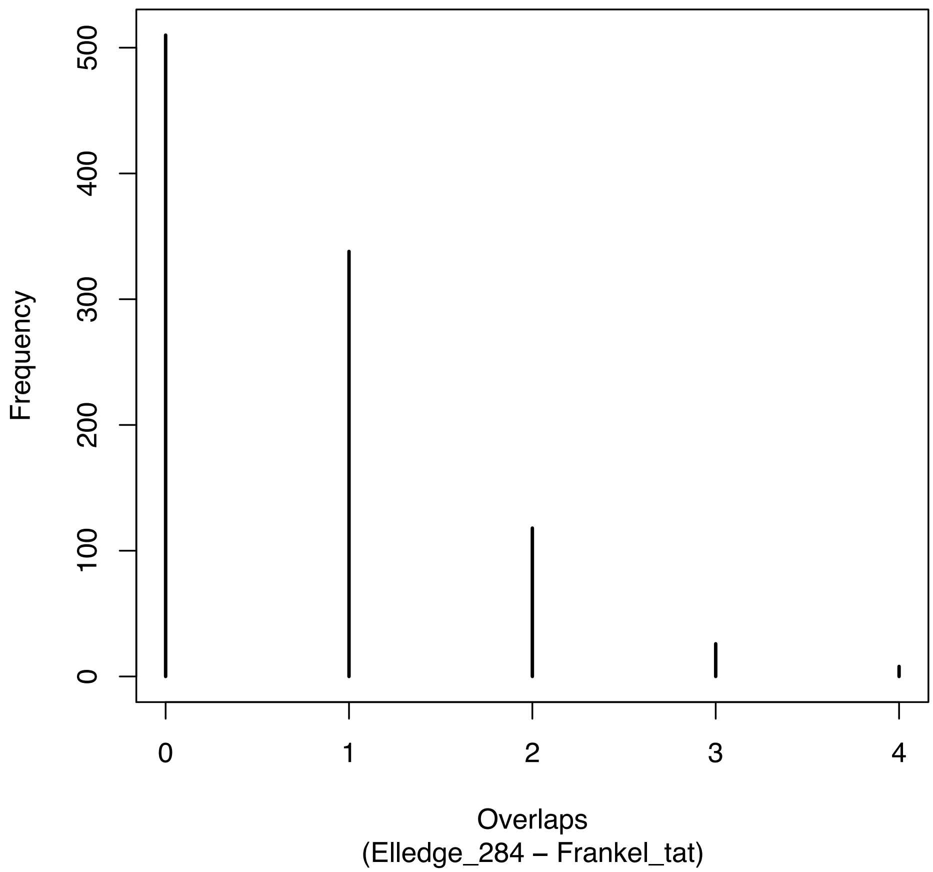

2.17 siRNA HIV Brass(283) vs. HARC Rev(56)

Total number of Genes overlapping: 2
Overlapping Genes: DDX3X, HUWE1,
Backgrounds Used:

```
  Name                         Size
1 "Y_moch_ElledgeBackground" "20515"
2 "NCBI_EntrezProtGenes"     "25157"
```

Hypergeometric p-value: 0.02028767
Simulation p-value:
Number of counts that had equal to or greater overlap than (2) in 1000 permutations: 440 => p-value: 0.44

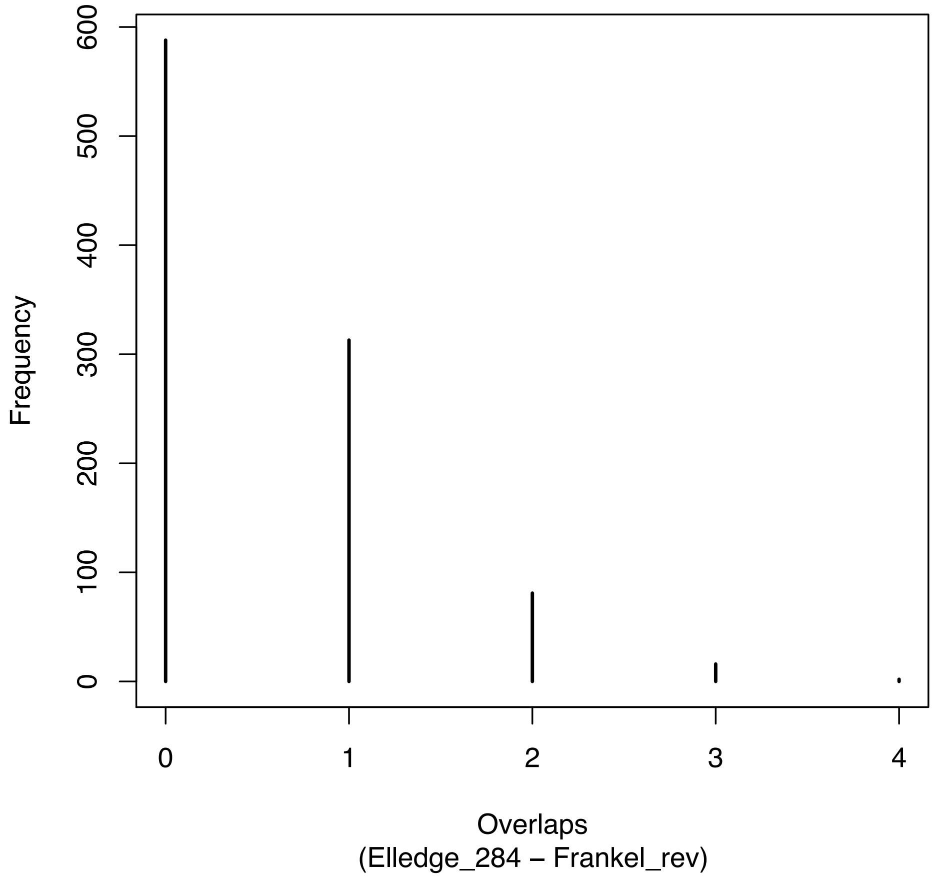

2.18 siRNA HIV Brass(283) vs. BIND HIV IN(23)

Total number of Genes overlapping: 0
Overlapping Genes: ,
Backgrounds Used:

```
  Name                        Size
1 "Y_moch_ElledgeBackground" "20515"
2 "NCBI_EntrezProtGenes"     "25157"
```

Hypergeometric p-value: 1
Simulation p-value:
Number of counts that had equal to or greater overlap than (0) in 1000 permutations: 1000 => p-value: 1

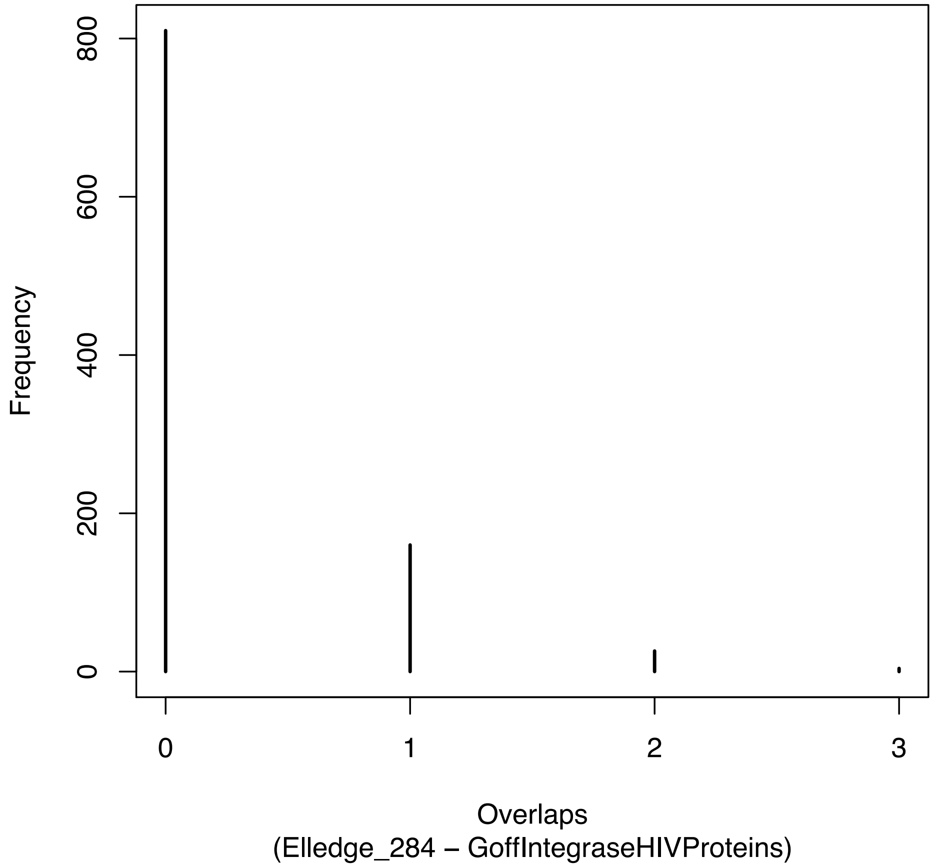

2.19 siRNA HIV Brass(283) vs. NCBI Interactions(1434)

Total number of Genes overlapping: 39
Overlapping Genes: NUP153, VPRBP, CD4, CTDP1, HGS, CCNT1, SIP1, NUP85, CXCR4, TFAP4, TCEB3, SSB, SPTBN1, SPTAN1, RELA, RANBP1, PURA, PSME2, PPP2R2A, PPIB, NMT1, NF2, ARF1, JAK1, SP110, DNAJB1, H3F3A, HTATSF1, KAT2A, FBXW11, ERCC3, AKT1, EGFR, EGF, DDX3X, AP2M1, POLR3A, POLR3F, PDIA6,
Backgrounds Used:

```
  Name                        Size
1 "Y_moch_ElledgeBackground" "20515"
2 "NCBI_EntrezProtGenes"     "25157"
```

Hypergeometric p-value: <0.001
Simulation p-value:
Number of counts that had equal to or greater overlap than (39) in 1000 permutations: 0 => p-value: <0.001

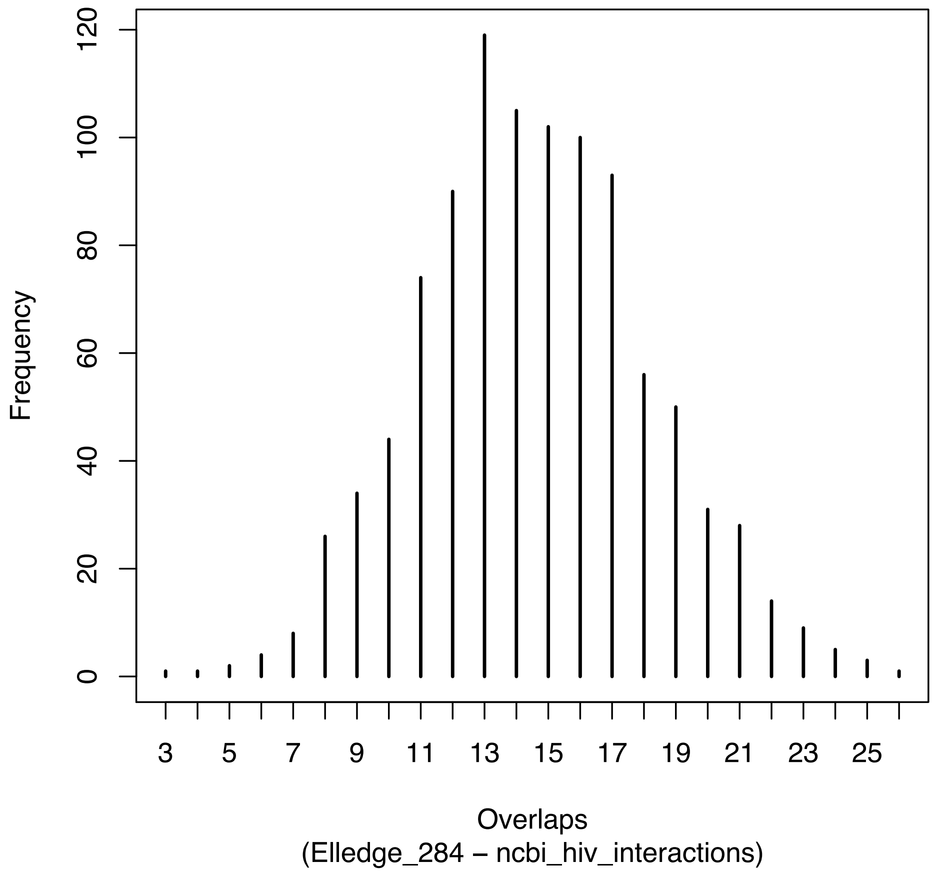

2.20 siRNA HIV Brass(283) vs. siRNA Flu Fly(98)

Total number of Genes overlapping: 3
Overlapping Genes: NUP153, EIF3H, RAB1B,
Backgrounds Used:

```
  Name                          Size
1 "Y_moch_ElledgeBackground" "20515"
2 "Fly_RNAi_background"      "19950"
```

Hypergeometric p-value: 0.03389694
Simulation p-value:
Number of counts that had equal to or greater overlap than (3) in 1000 permutations: 125 => p-value: 0.125

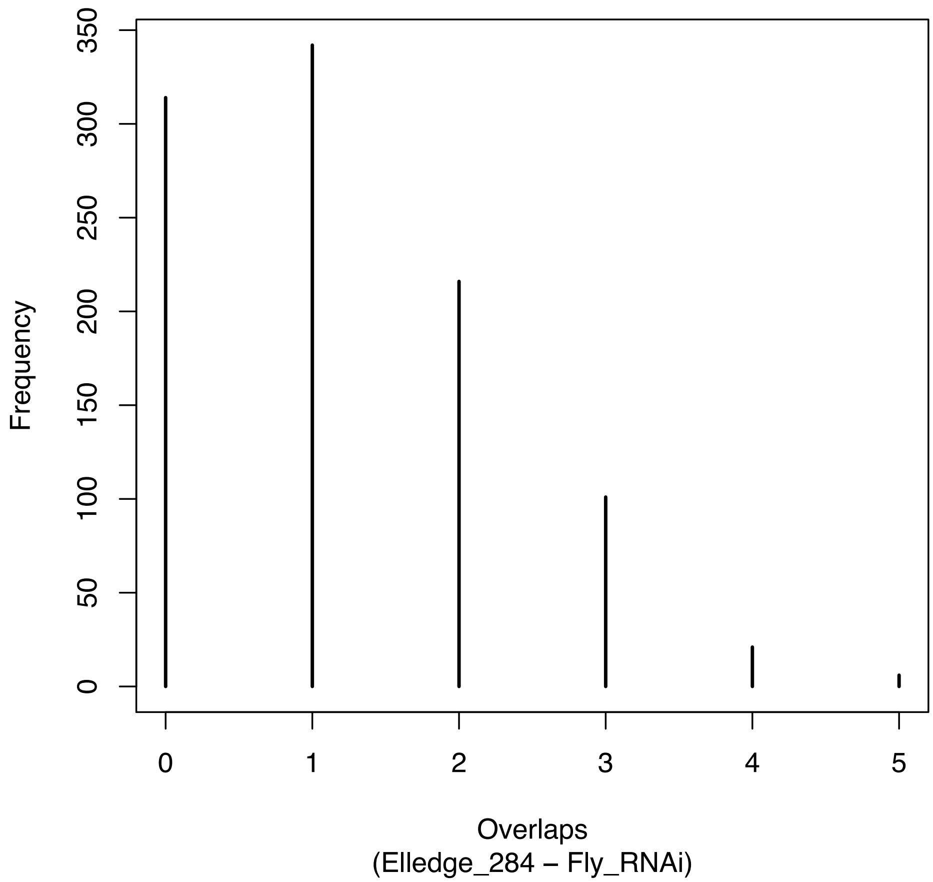

2.21 siRNA HIV Brass(283) vs. siRNA WNV(305)

Total number of Genes overlapping: 9
Overlapping Genes: RUSC2, USP6, CRTC3, MID1IP1, TRAPPC1, KLHL1, ATP6V0A1, SCFD1, RIMS4,
Backgrounds Used:

```
  Name                         Size
1 "Y_moch_ElledgeBackground" "20515"
2 "NCBI_EntrezProtGenes"     "25157"
```

Hypergeometric p-value: 0.001373285
Simulation p-value:
Number of counts that had equal to or greater overlap than (9) in 1000 permutations: 4 => p-value: 0.004

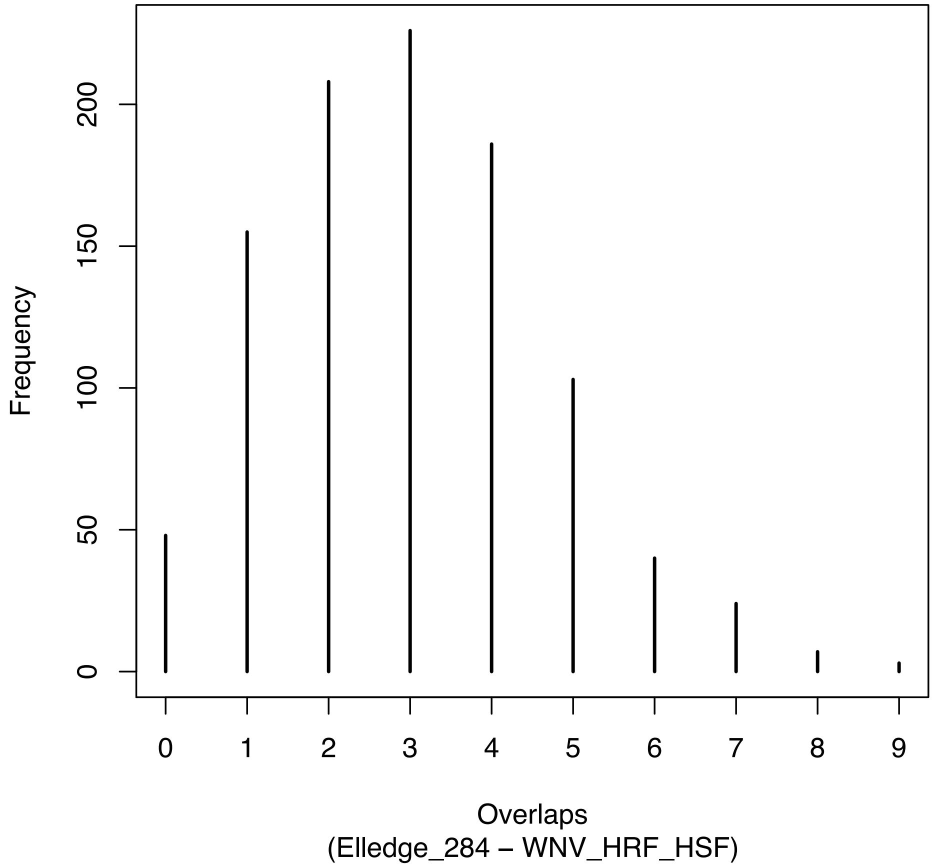

2.22 siRNA HIV Zhou(303) vs. SNP HIV Fellay(63)

Total number of Genes overlapping: 1
Overlapping Genes: MICB,
Backgrounds Used:

```
  Name                    Size
1 "MercksiRNA_Background" "16450"
2 "Y_UngarBackground"     "22495"
```

Hypergeometric p-value: 0.2011522
Simulation p-value:
Number of counts that had equal to or greater overlap than (1) in 1000 permutations: 541 => p-value: 0.541

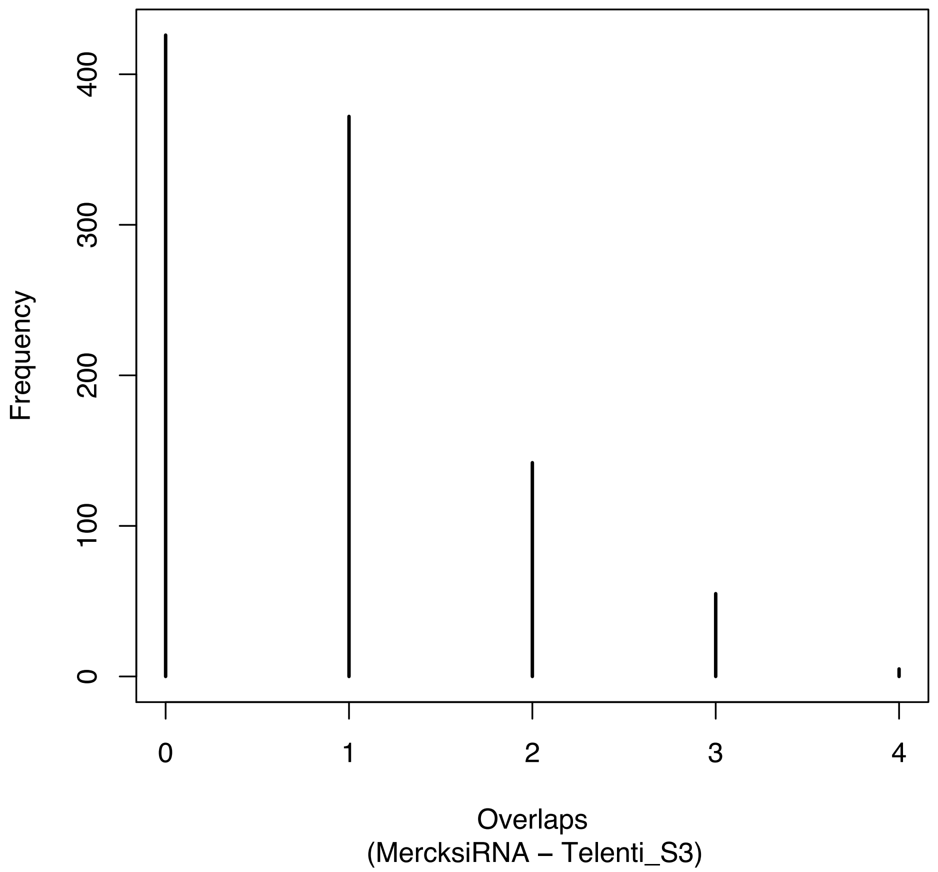

2.23 siRNA HIV Zhou(303) vs. Particle Associated HIV(248)

Total number of Genes overlapping: 6
Overlapping Genes: CCT2, PDIA3, ATP5B, RAB8A, RALB, TSG101,
Backgrounds Used:

```
  Name                     Size
1 "MercksiRNA_Background" "16450"
2 "NCBI_EntrezProtGenes"  "25157"
```

Hypergeometric p-value: 0.02756199
Simulation p-value:
Number of counts that had equal to or greater overlap than (6) in 1000 permutations: 70 => p-value: 0.07

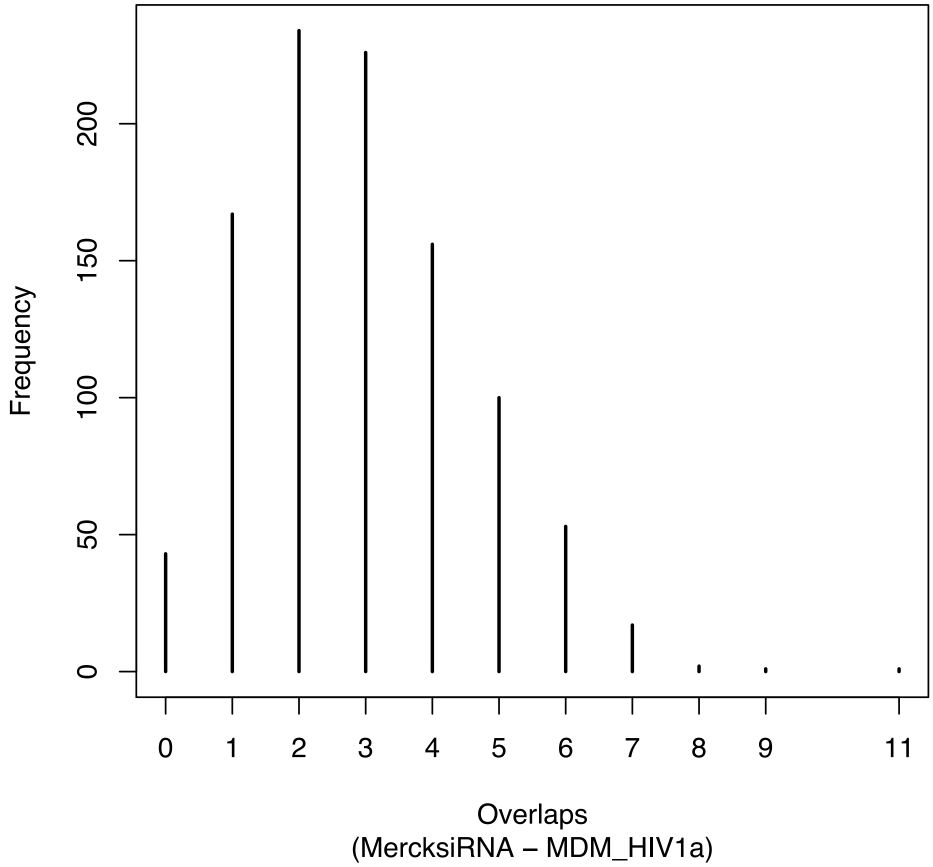

2.24 siRNA HIV Zhou(303) vs. HARC Nef(6)

Total number of Genes overlapping: 0
Overlapping Genes: ,
Backgrounds Used:

```
  Name                     Size
1 "MercksiRNA_Background" "16450"
2 "NCBI_EntrezProtGenes"  "25157"
```

Hypergeometric p-value: 1
Simulation p-value:
Number of counts that had equal to or greater overlap than (0) in 1000 permutations: 1000 => p-value: 1

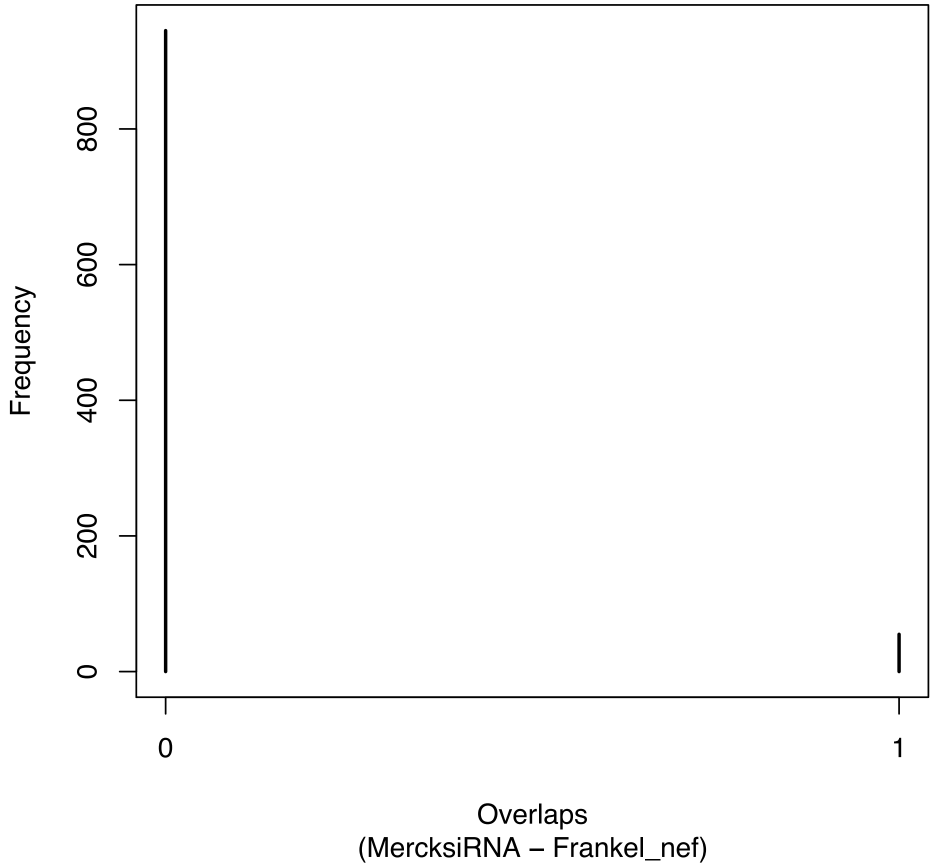

2.25 siRNA HIV Zhou(303) vs. HARC Tat(69)

Total number of Genes overlapping: 4
Overlapping Genes: CCT2, DDX3X, STIP1, CCNT1,
Backgrounds Used:

```
  Name                     Size
1 "MercksiRNA_Background" "16450"
2 "NCBI_EntrezProtGenes"  "25157"
```

Hypergeometric p-value: 0.001305099
Simulation p-value:
Number of counts that had equal to or greater overlap than (4) in 1000 permutations: 52 => p-value: 0.052

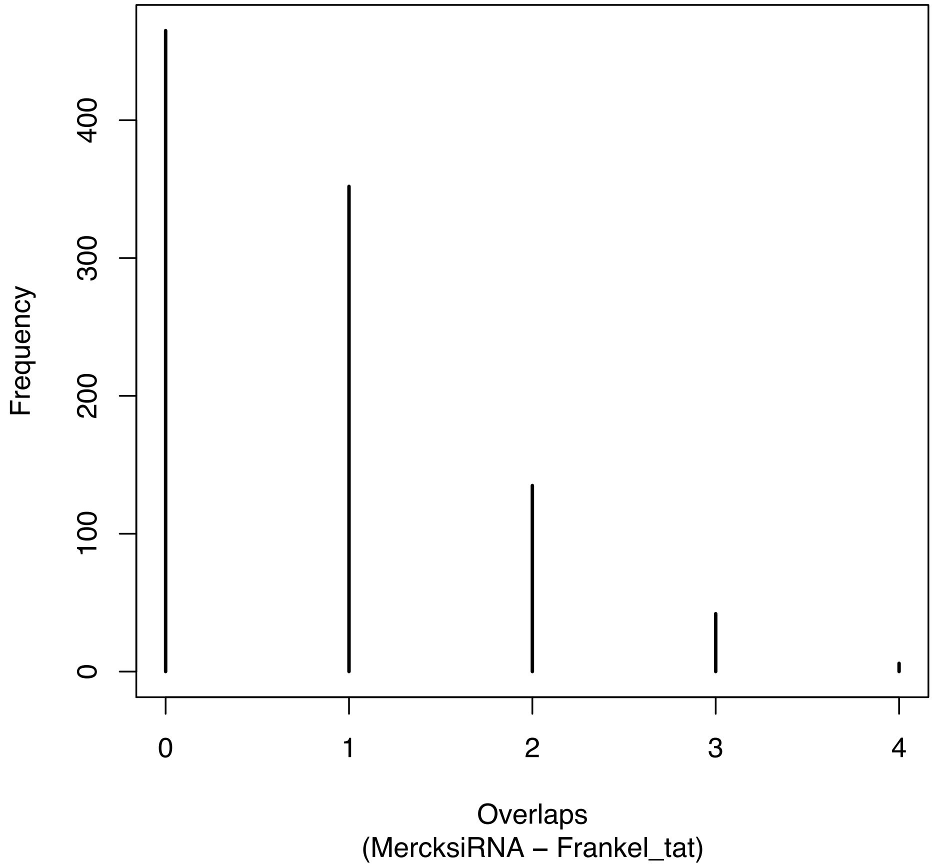

2.26 siRNA HIV Zhou(303) vs. HARC Rev(56)

Total number of Genes overlapping: 2
Overlapping Genes: UBR5, DDX3X,
Backgrounds Used:

```
  Name                     Size
1 "MercksiRNA_Background" "16450"
2 "NCBI_EntrezProtGenes"  "25157"
```

Hypergeometric p-value: 0.02802283
Simulation p-value:
Number of counts that had equal to or greater overlap than (2) in 1000 permutations: 469 => p-value: 0.469

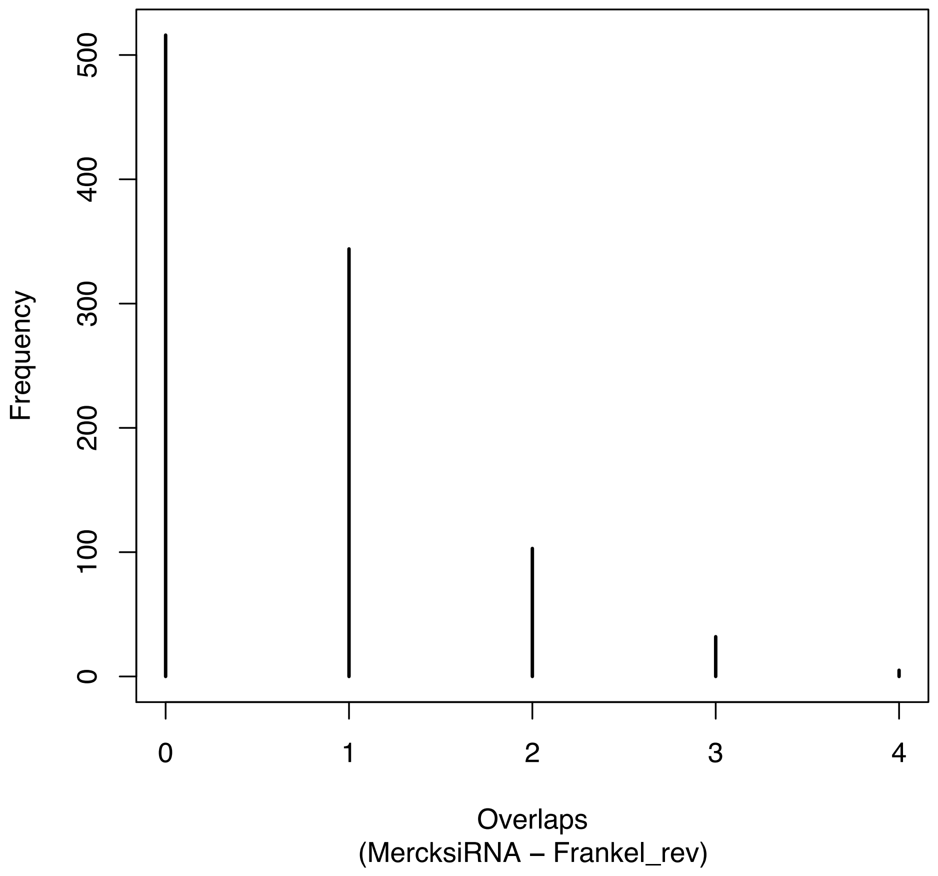

2.27 siRNA HIV Zhou(303) vs. BIND HIV IN(23)

Total number of Genes overlapping: 1
Overlapping Genes: BAZ2B,
Backgrounds Used:

```
  Name                     Size
1 "MercksiRNA_Background" "16450"
2 "NCBI_EntrezProtGenes"  "25157"
```

Hypergeometric p-value: 0.02947518
Simulation p-value:
Number of counts that had equal to or greater overlap than (1) in 1000 permutations: 232 => p-value: 0.232

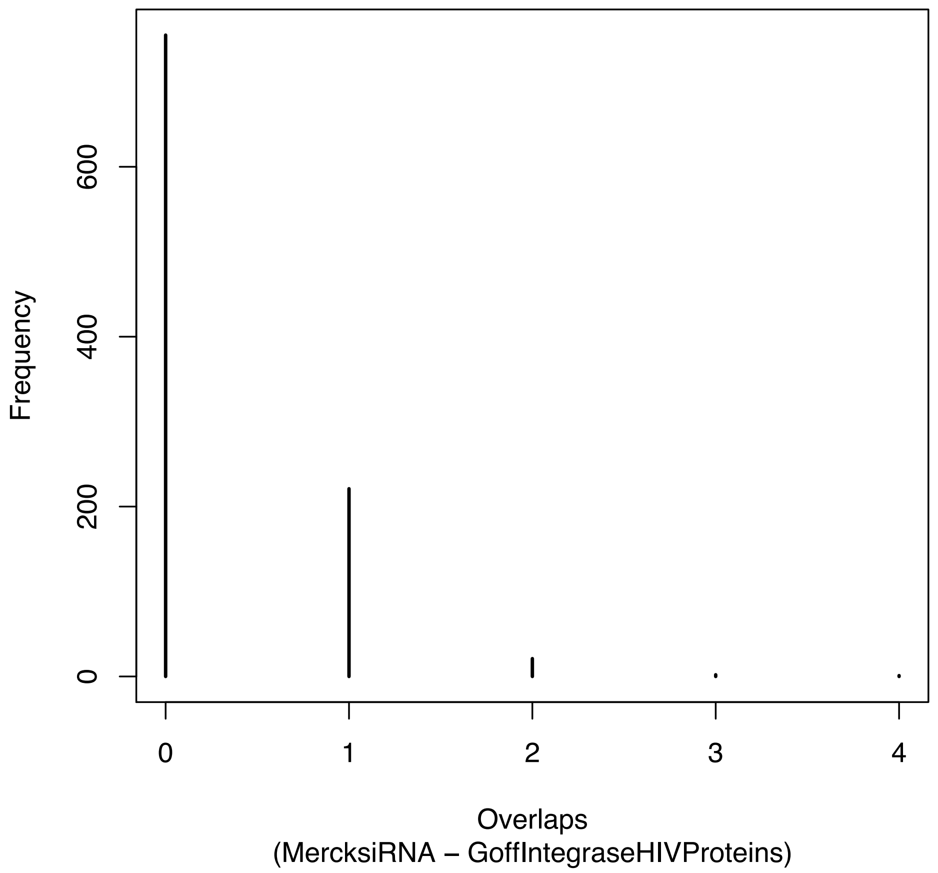

2.28 siRNA HIV Zhou(303) vs. NCBI Interactions(1434)

Total number of Genes overlapping: 40
Overlapping Genes: DPP4, MMP9, PCSK6, PDE8A, GTF2A1, IL1A, JAK1, MAP3K14, NFKB1, POLR2H, POLR2K, POLR2L, PSMD4, SFRS2, PDIA3, AKT1, CD4, CHST1, CMKLR1, CYCS, DDX3X, F2, GRINA, HLA-DOA, PCK1, RELA, SDC1, TCEB3, TUBA8, TWF1, VDR, XPO1, BRCA1, CXCR4, GTF2H1, RAD23A, GTF3C3, TNFSF11, CCNT1, TSG101,
Backgrounds Used:

	Name	Size
1	"MercksiRNA_Background"	"16450"
2	"NCBI_EntrezProtGenes"	"25157"

Hypergeometric p-value: <0.001
Simulation p-value:
Number of counts that had equal to or greater overlap than (40) in 1000 permutations: 0 => p-value: <0.001

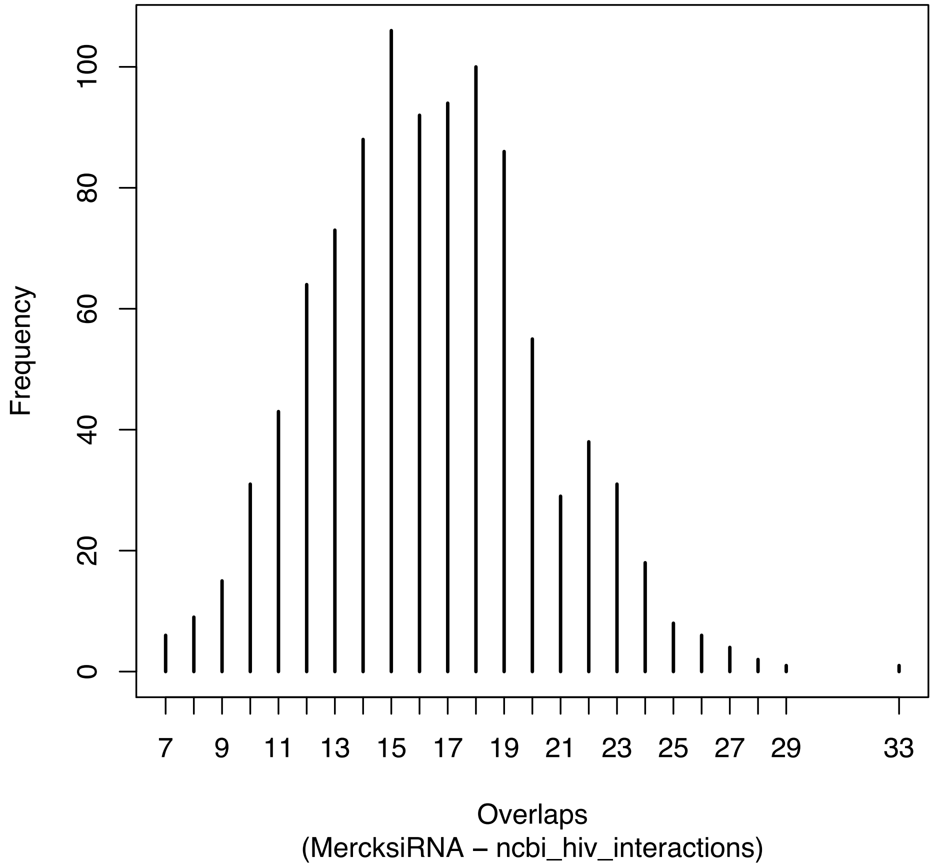

2.29 siRNA HIV Zhou(303) vs. siRNA Flu Fly(98)

Total number of Genes overlapping: 1
Overlapping Genes: ATP5B,
Backgrounds Used:

```
  Name                    Size
1 "MercksiRNA_Background" "16450"
2 "Fly_RNAi_background"   "19950"
```

Hypergeometric p-value: 0.411969
Simulation p-value:
Number of counts that had equal to or greater overlap than (1) in 1000 permutations: 738 => p-value: 0.738

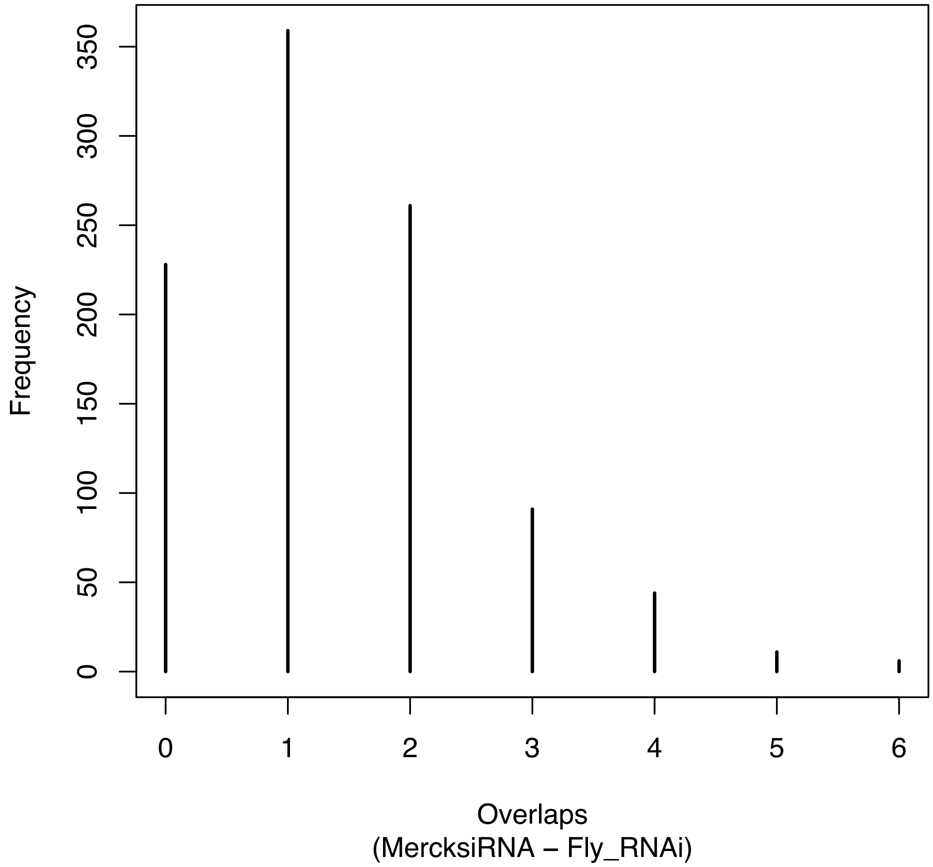

2.30 siRNA HIV Zhou(303) vs. siRNA WNV(305)

Total number of Genes overlapping: 3
Overlapping Genes: KIF17, SEC61G, CFHR5,
Backgrounds Used:

```
  Name                     Size
1 "MercksiRNA_Background" "16450"
2 "NCBI_EntrezProtGenes"  "25157"
```

Hypergeometric p-value: 0.4810913
Simulation p-value:
Number of counts that had equal to or greater overlap than (3) in 1000 permutations: 693 => p-value: 0.693

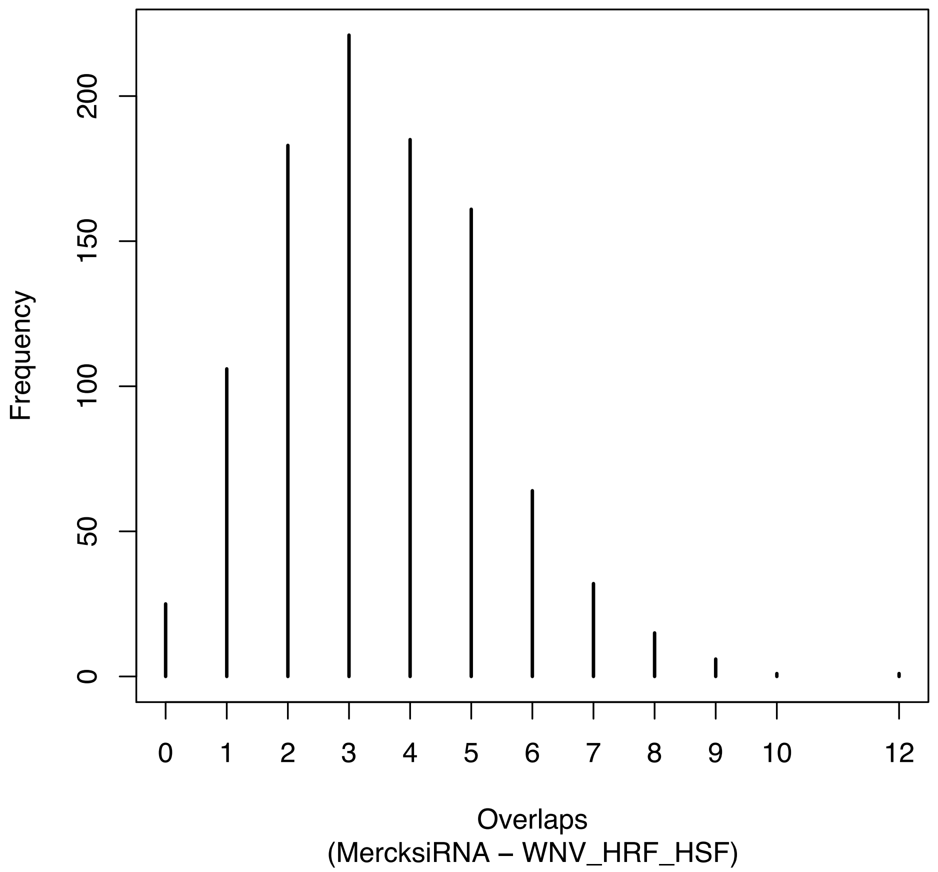

2.31 SNP HIV Fellay(63) vs. Particle Associated HIV(248)

Total number of Genes overlapping: 2
Overlapping Genes: HLA-C, CYFIP1,
Backgrounds Used:

```
  Name                    Size
1 "Y_UngarBackground"     "22495"
2 "NCBI_EntrezProtGenes"  "25157"
```

Hypergeometric p-value: 0.02013418
Simulation p-value:
Number of counts that had equal to or greater overlap than (2) in 1000 permutations: 108 => p-value: 0.108

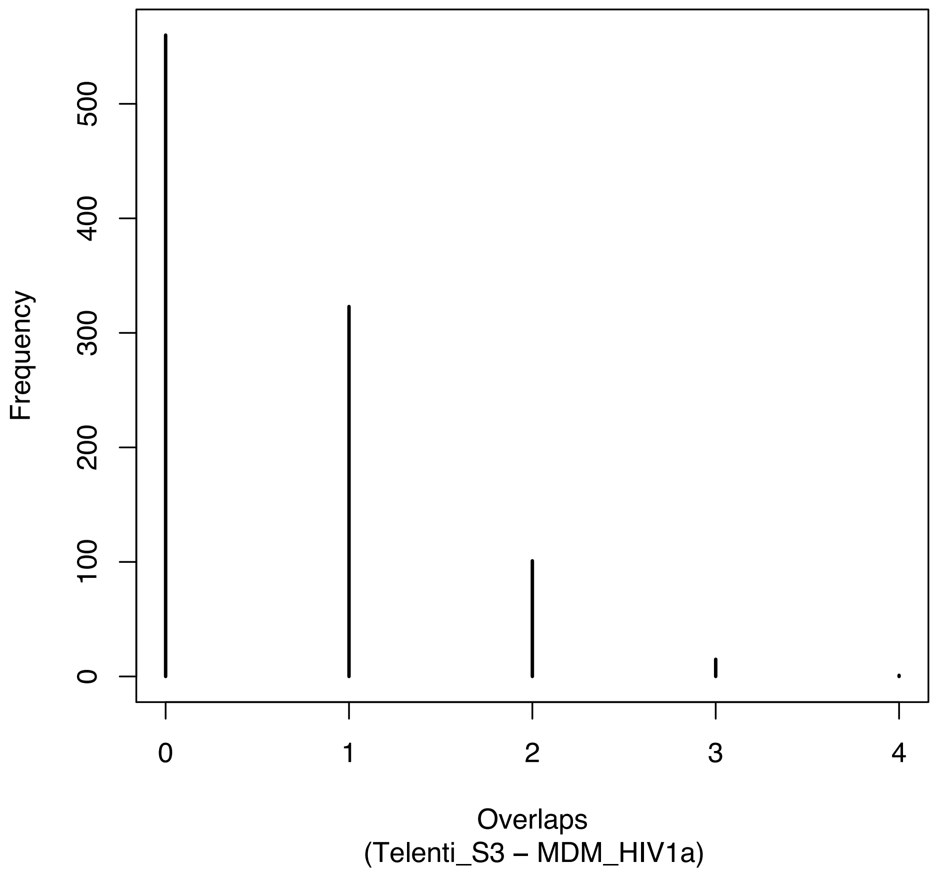

2.32 SNP HIV Fellay(63) vs. HARC Nef(6)

Total number of Genes overlapping: 0
Overlapping Genes: ,
Backgrounds Used:

```
  Name                    Size
1 "Y_UngarBackground"     "22495"
2 "NCBI_EntrezProtGenes" "25157"
```

Hypergeometric p-value: 1
Simulation p-value:
Number of counts that had equal to or greater overlap than (0) in 1000 permutations: 1000 => p-value: 1

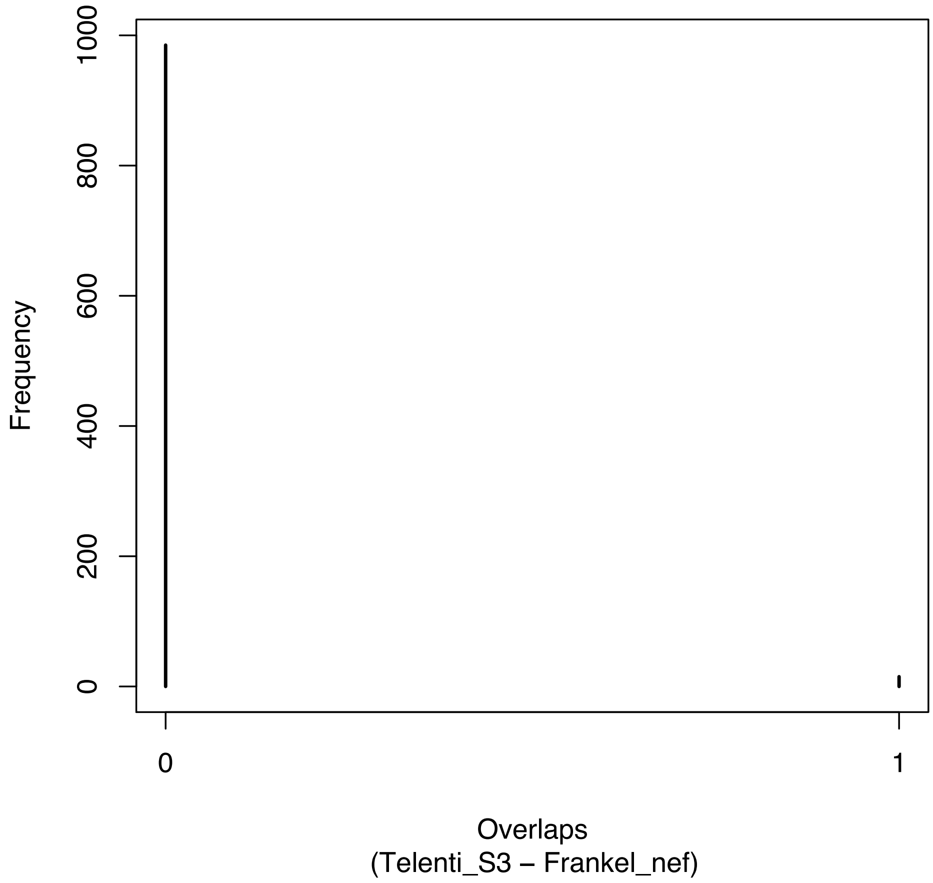

2.33 SNP HIV Fellay(63) vs. HARC Tat(69)

Total number of Genes overlapping: 0
Overlapping Genes: ,
Backgrounds Used:

```
  Name                    Size
1 "Y_UngarBackground"     "22495"
2 "NCBI_EntrezProtGenes"  "25157"
```

Hypergeometric p-value: 1
Simulation p-value:
Number of counts that had equal to or greater overlap than (0) in 1000 permutations: 1000 => p-value: 1

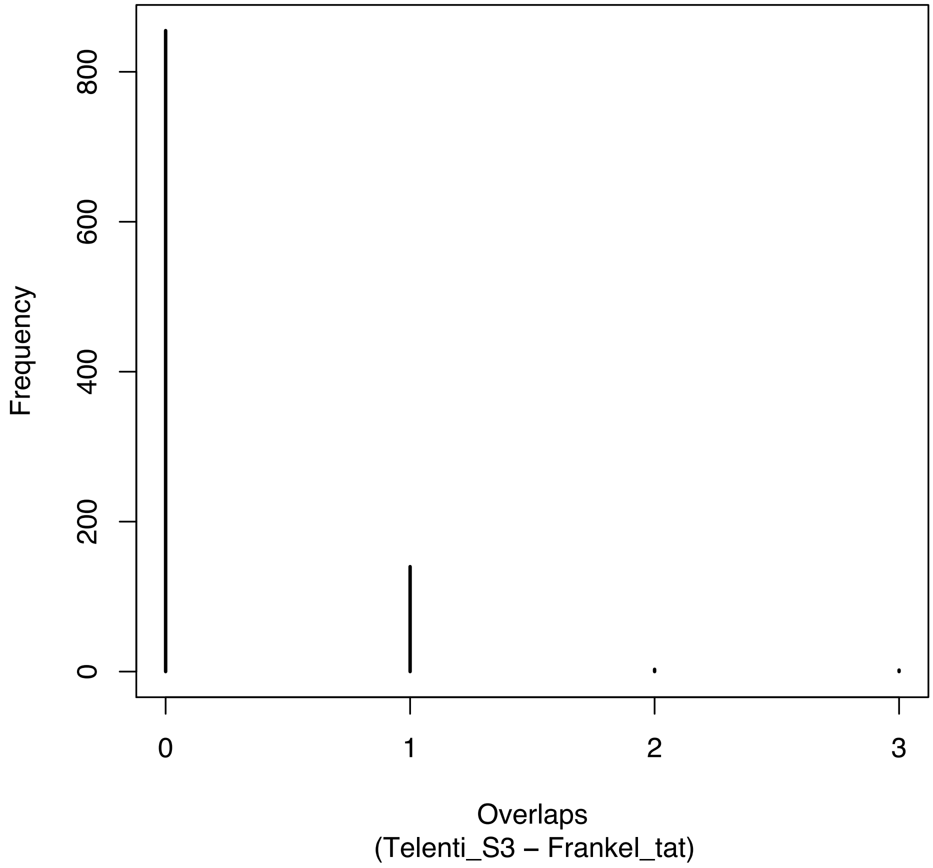

2.34 SNP HIV Fellay(63) vs. HARC Rev(56)

Total number of Genes overlapping: 0
Overlapping Genes: ,
Backgrounds Used:

```
  Name                    Size
1 "Y_UngarBackground"     "22495"
2 "NCBI_EntrezProtGenes"  "25157"
```

Hypergeometric p-value: 1
Simulation p-value:
Number of counts that had equal to or greater overlap than (0) in 1000 permutations: 1000 => p-value: 1

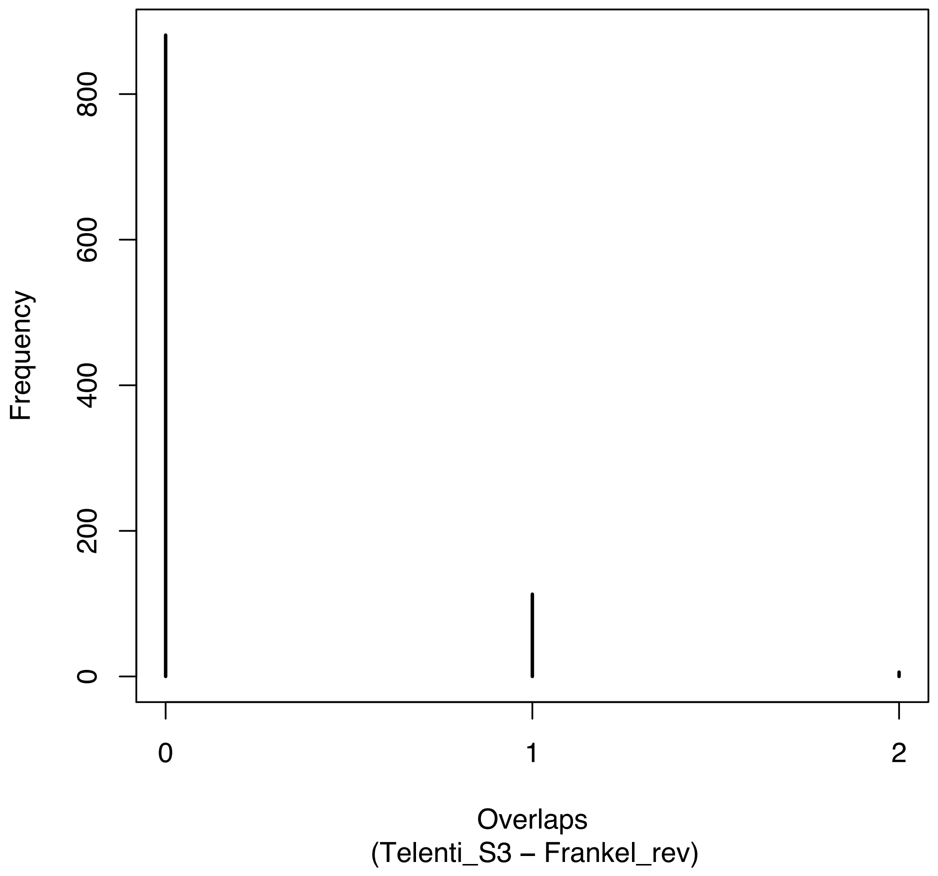

2.35 SNP HIV Fellay(63) vs. BIND HIV IN(23)

Total number of Genes overlapping: 0
Overlapping Genes: ,
Backgrounds Used:

```
  Name                    Size
1 "Y_UngarBackground"     "22495"
2 "NCBI_EntrezProtGenes" "25157"
```

Hypergeometric p-value: 1
Simulation p-value:
Number of counts that had equal to or greater overlap than (0) in 1000 permutations: 1000 => p-value: 1

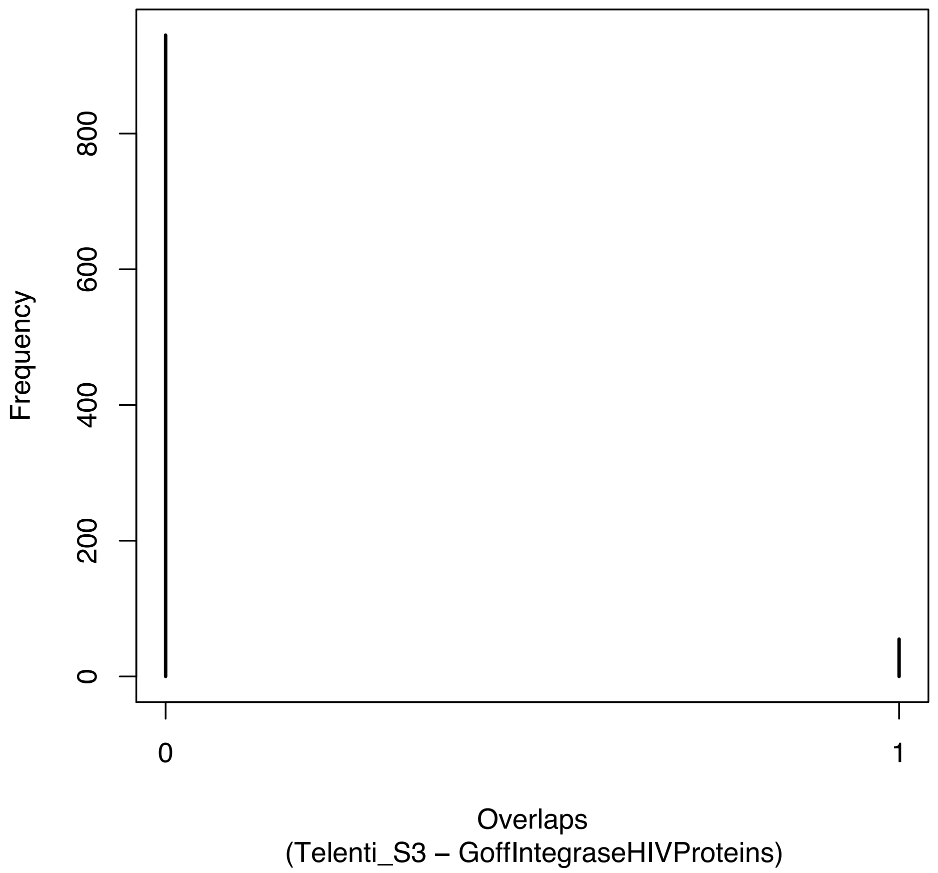

2.36 SNP HIV Fellay(63) vs. NCBI Interactions(1434)

Total number of Genes overlapping: 5
Overlapping Genes: HLA-C, CHMP4C, UBD, DICER1, HLA-B,
Backgrounds Used:

	Name	Size
1	"Y_UngarBackground"	"22495"
2	"NCBI_EntrezProtGenes"	"25157"

Hypergeometric p-value: 0.1156355
Simulation p-value:
Number of counts that had equal to or greater overlap than (5) in 1000 permutations: 234 => p-value: 0.234

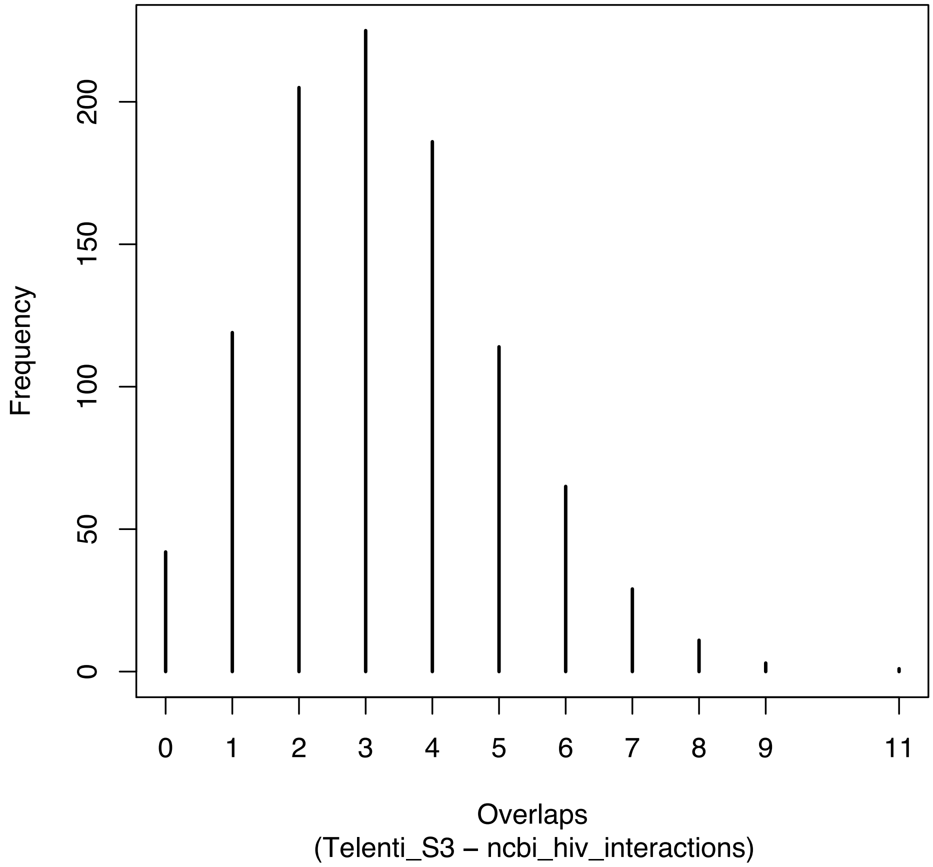

2.37 SNP HIV Fellay(63) vs. siRNA Flu Fly(98)

Total number of Genes overlapping: 0
Overlapping Genes: ,
Backgrounds Used:

```
  Name                   Size
1 "Y_UngarBackground"    "22495"
2 "Fly_RNAi_background" "19950"
```

Hypergeometric p-value: 1
Simulation p-value:
Number of counts that had equal to or greater overlap than (0) in 1000 permutations: 1000 => p-value: 1

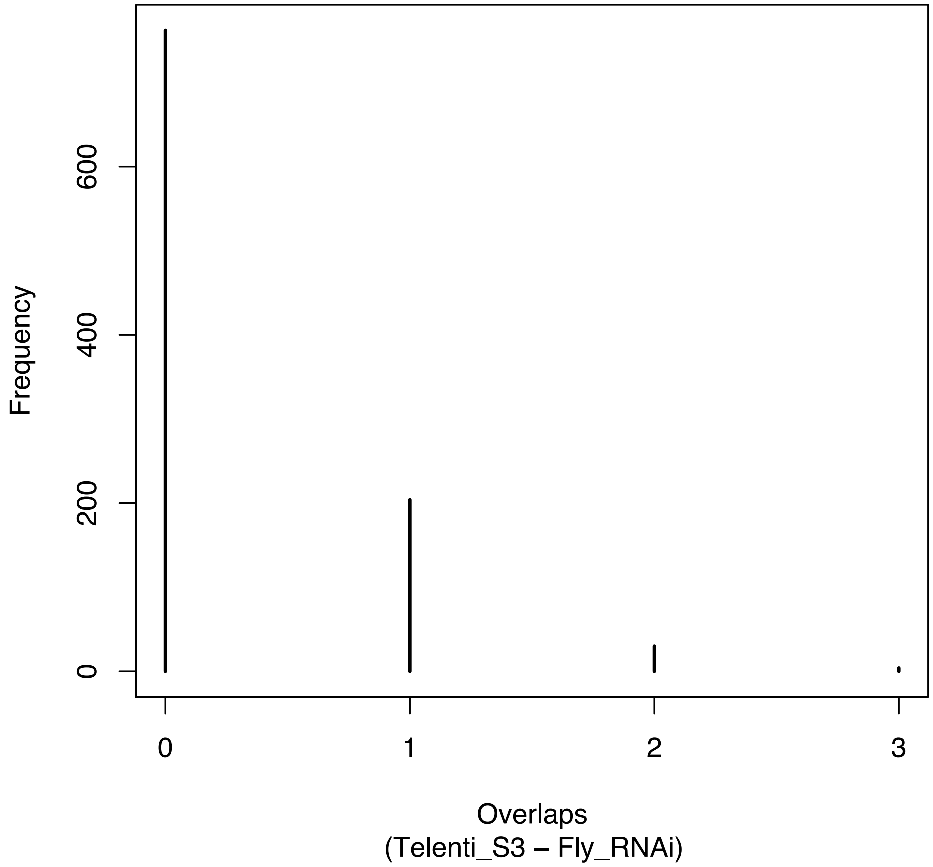

2.38 SNP HIV Fellay(63) vs. siRNA WNV(305)

Total number of Genes overlapping: 2
Overlapping Genes: SEPT5, SLC39A11,
Backgrounds Used:

	Name	Size
1	"Y_UngarBackground"	"22495"
2	"NCBI_EntrezProtGenes"	"25157"

Hypergeometric p-value: 0.03422128
Simulation p-value:
Number of counts that had equal to or greater overlap than (2) in 1000 permutations: 140 => p-value: 0.14

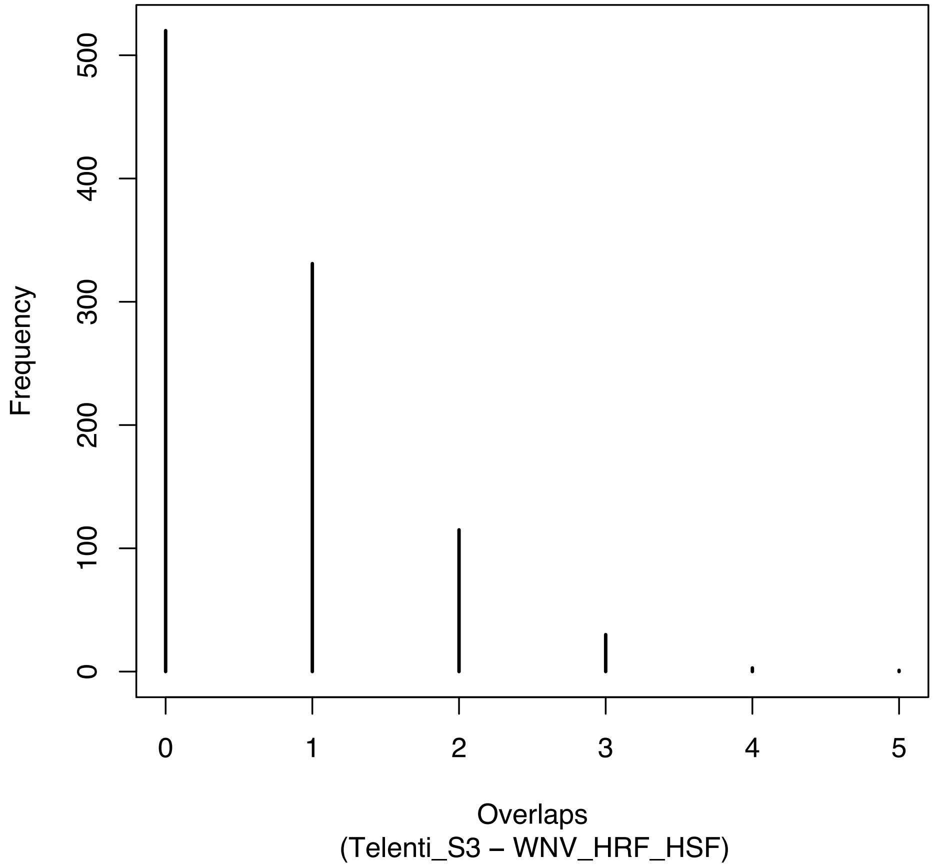

2.39 Particle Associated HIV(248) vs. HARC Nef(6)

Total number of Genes overlapping: 2
Overlapping Genes: S100A10, ANXA6,
Backgrounds Used:

```
  Name                   Size
1 "NCBI_EntrezProtGenes" "25157"
2 "NCBI_EntrezProtGenes" "25157"
```

Hypergeometric p-value: <0.001
Simulation p-value:
Number of counts that had equal to or greater overlap than (2) in 1000 permutations: 0 => p-value: <0.001

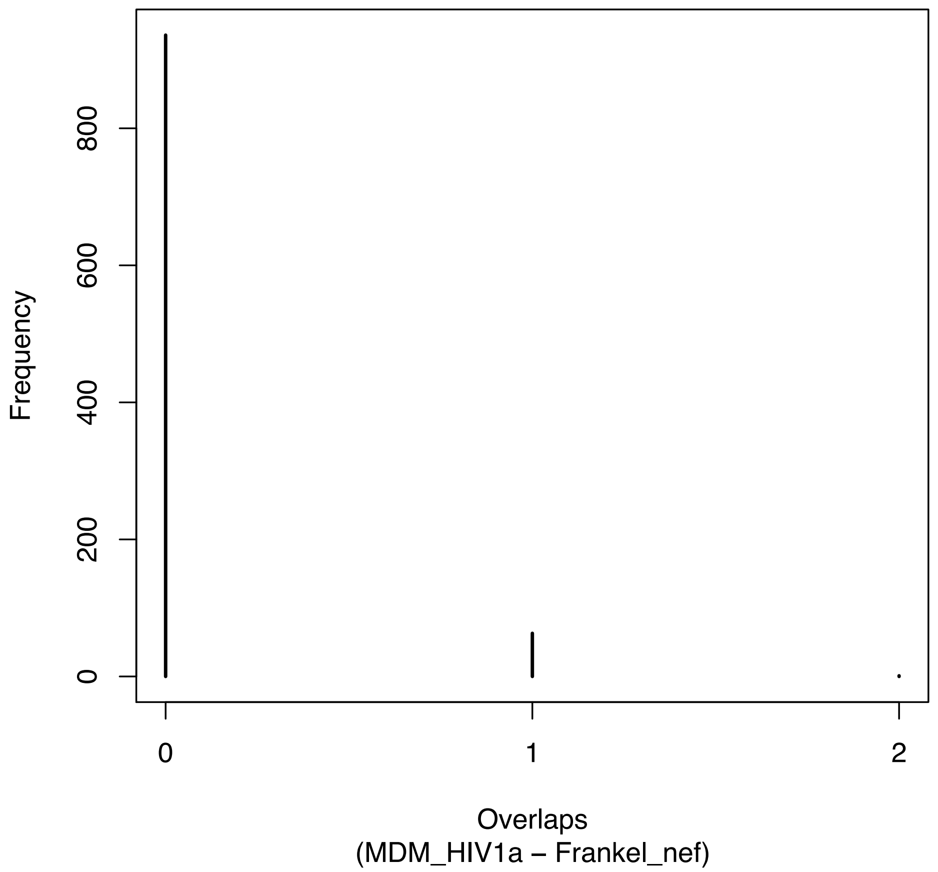

2.40 Particle Associated HIV(248) vs. HARC Tat(69)

Total number of Genes overlapping: 3
Overlapping Genes: CCT2, ALB, ATP1A1,
Backgrounds Used:

```
  Name                    Size
1 "NCBI_EntrezProtGenes" "25157"
2 "NCBI_EntrezProtGenes" "25157"
```

Hypergeometric p-value: 0.004827426
Simulation p-value:
Number of counts that had equal to or greater overlap than (3) in 1000 permutations: 27 => p-value: 0.027

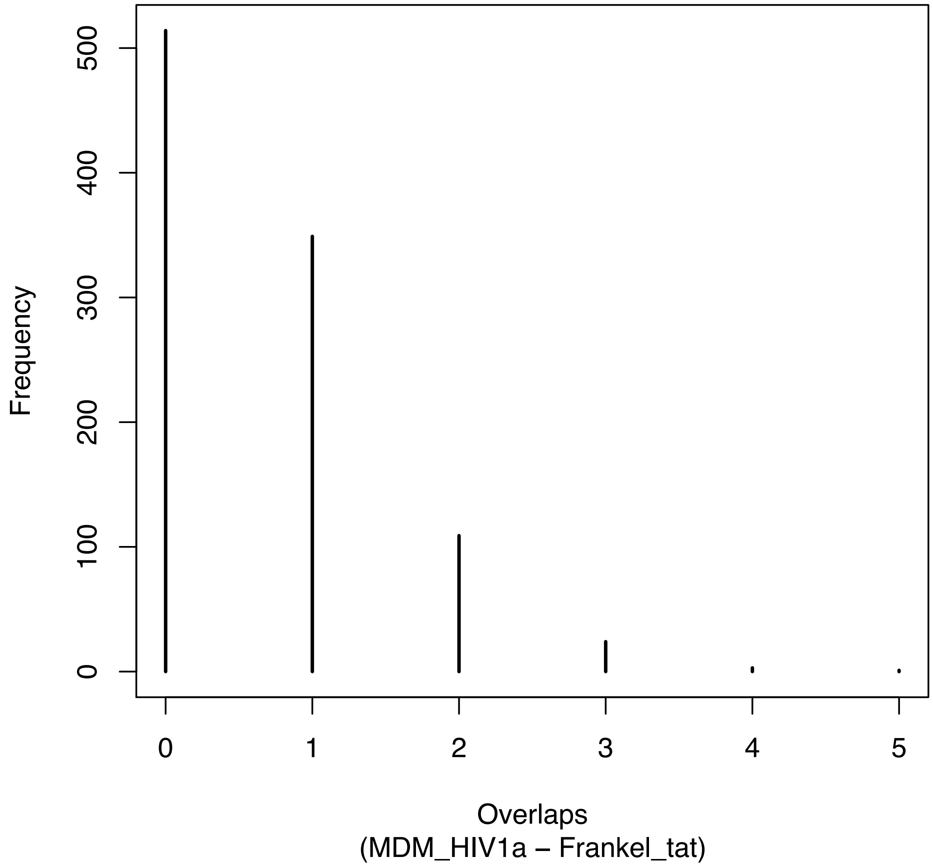

2.41 Particle Associated HIV(248) vs. HARC Rev(56)

Total number of Genes overlapping: 10
Overlapping Genes: PFN1, PPIA, CFL1, YWHAZ, PRDX1, ANXA1, PKM2, HSPA9, VCP, EEF2,
Backgrounds Used:

```
  Name                   Size
1 "NCBI_EntrezProtGenes" "25157"
2 "NCBI_EntrezProtGenes" "25157"
```

Hypergeometric p-value: <0.001
Simulation p-value:
Number of counts that had equal to or greater overlap than (10) in 1000 permutations: 0 => p-value: <0.001

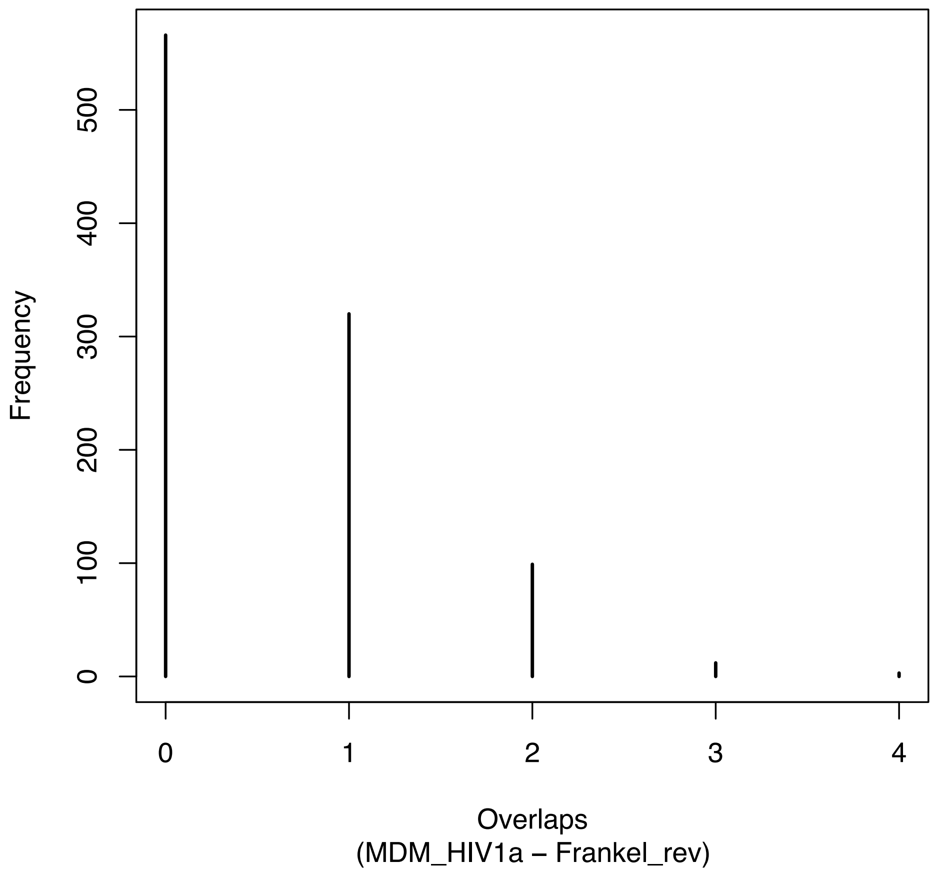

2.42 Particle Associated HIV(248) vs. BIND HIV IN(23)

Total number of Genes overlapping: 1
Overlapping Genes: RDX,
Backgrounds Used:

```
  Name                   Size
1 "NCBI_EntrezProtGenes" "25157"
2 "NCBI_EntrezProtGenes" "25157"
```

Hypergeometric p-value: 0.02136938
Simulation p-value:
Number of counts that had equal to or greater overlap than (1) in 1000 permutations: 191 => p-value: 0.191

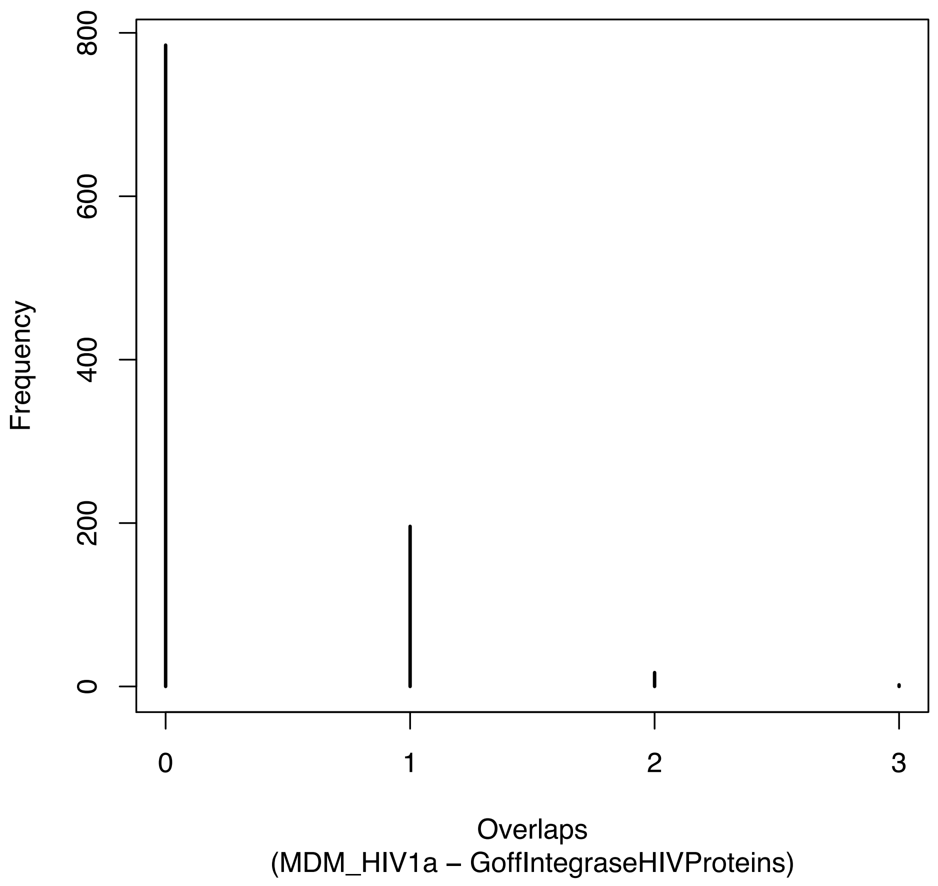

2.43 Particle Associated HIV(248) vs. NCBI Interactions(1434)

Total number of Genes overlapping: 94
Overlapping Genes: PDE3B, ACTN4, HIST4H4, B2M, SDC2, HIST1H3B, LGALS1, PPIA, CFL1, RHOG, ARF1, CDC42, PPIB, CD81, TSG101, CD9, SDCBP, RAB11A, YWHAZ, RAN, LGALS3, TIMP2, YWHAB, TNFSF14, VPS28, RHOA, RAC1, RAC2, PSME1, EEF1A2, HLA-DRA, ANXA2, APOE, FBP1, PURA, PLCH2, PTBP1, ACTB, ACTA2, CNP, HLA-A, HLA-C, CD82, VIM, PDIA3, ENO1, CD14, BSG, TUBA4A, CD2, RDX, LCP1, MSN, HSPA9, HSPA5, HSPA8, HSPA2, HSPA1B, CD58, HSPD1, CAT, LAMP1, SLC3A2, ICAM1, ALB, GSN, HSP90AA1, STAT1, CYBB, PSMD2, ITGB2, SPN, CD44, ITGB3, ITGA3, CD86, PDCD6IP, EEF2, KPNB1, VCL, ITGAM, ITGAX, ITGB1, ITGA5, ITGAV, THBS1, PTPRC, ITPR3, FN1, CLTC, TLN1, C3, FLNA, PLEC1,
Backgrounds Used:

```
  Name                    Size
1 "NCBI_EntrezProtGenes" "25157"
2 "NCBI_EntrezProtGenes" "25157"
```

Hypergeometric p-value: <0.001
Simulation p-value:
Number of counts that had equal to or greater overlap than (94) in 1000 permutations: 0 => p-value: <0.001

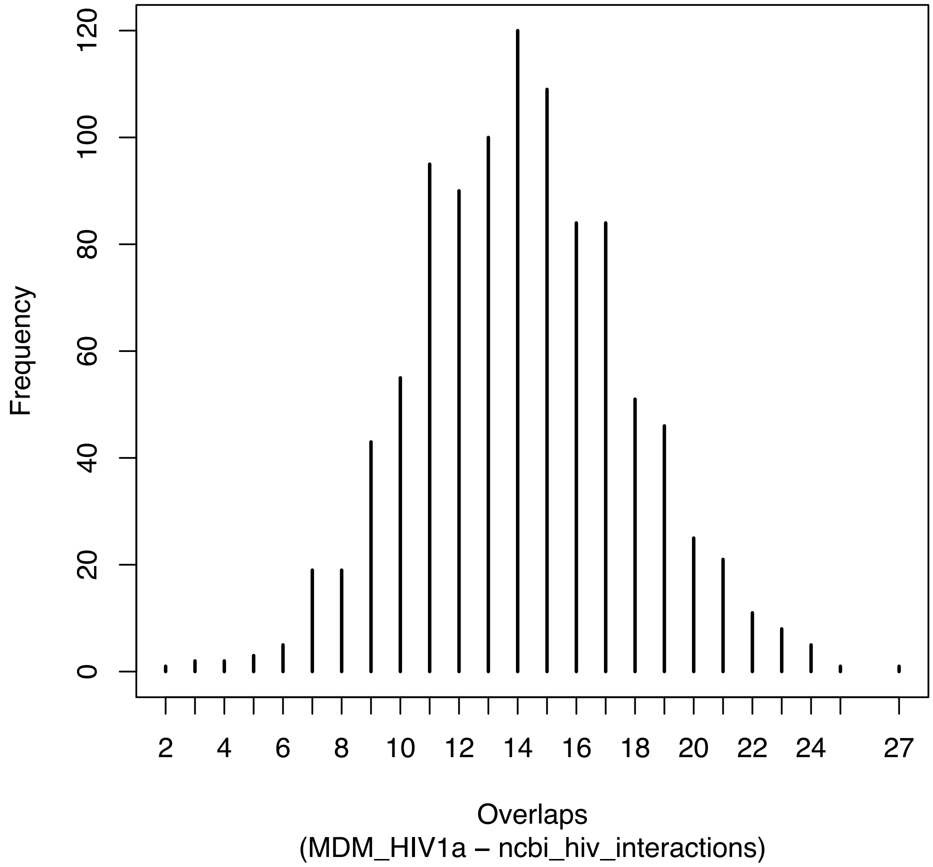

2.44 Particle Associated HIV(248) vs. siRNA Flu Fly(98)

Total number of Genes overlapping: 9
Overlapping Genes: RPS16, RAB10, RAB5A, ATP6V0D1, PGD, ATP5B, HSPA5, HSPA8, VCP,
Backgrounds Used:

```
  Name                    Size
1 "NCBI_EntrezProtGenes" "25157"
2 "Fly_RNAi_background"  "19950"
```

Hypergeometric p-value: <0.001
Simulation p-value:
Number of counts that had equal to or greater overlap than (9) in 1000 permutations: 0 => p-value: <0.001

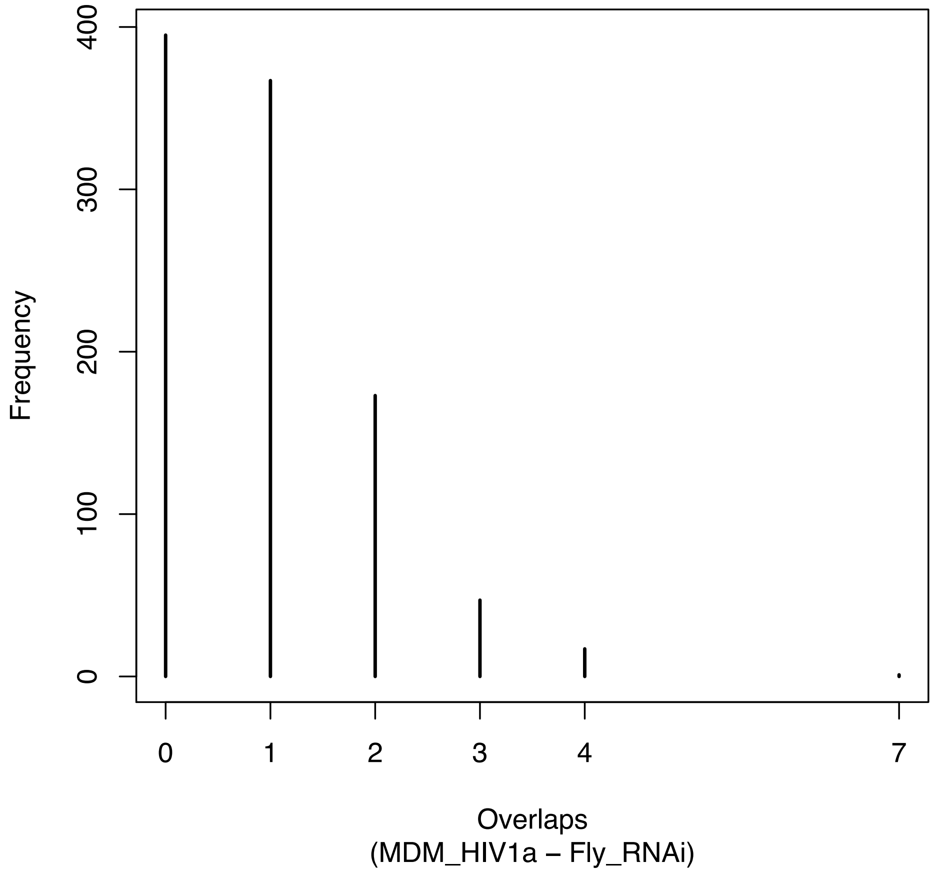

2.45 Particle Associated HIV(248) vs. siRNA WNV(305)

Total number of Genes overlapping: 8
Overlapping Genes: PDE3B, CLEC7A, BCAP31, CLIC1, ATP6V0D1, CNP, ATP6V1B2, ATP6V0A1,
Backgrounds Used:

```
  Name                    Size
1 "NCBI_EntrezProtGenes" "25157"
2 "NCBI_EntrezProtGenes" "25157"
```

Hypergeometric p-value: 0.003424005
Simulation p-value:
Number of counts that had equal to or greater overlap than (8) in 1000 permutations: 13 => p-value: 0.013

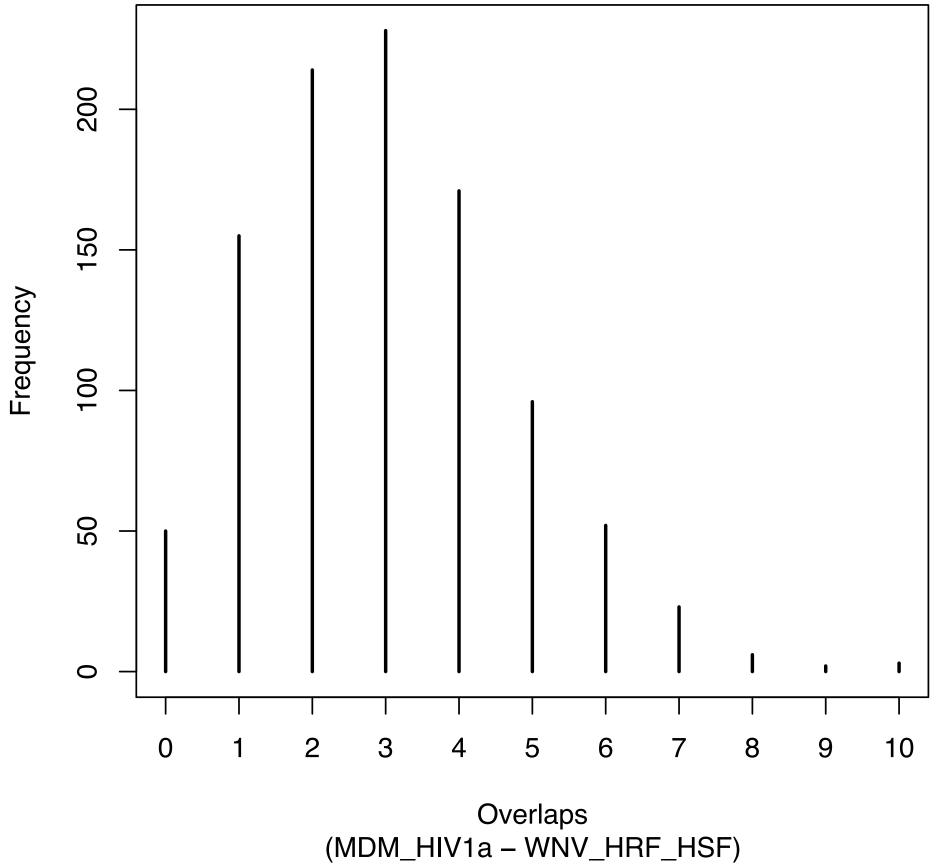

2.46 HARC Nef(6) vs. HARC Tat(69)

Total number of Genes overlapping: 0
Overlapping Genes: ,
Backgrounds Used:

```
  Name                     Size
1 "NCBI_EntrezProtGenes" "25157"
2 "NCBI_EntrezProtGenes" "25157"
```

Hypergeometric p-value: 1
Simulation p-value:
Number of counts that had equal to or greater overlap than (0) in 1000 permutations: 1000 => p-value: 1

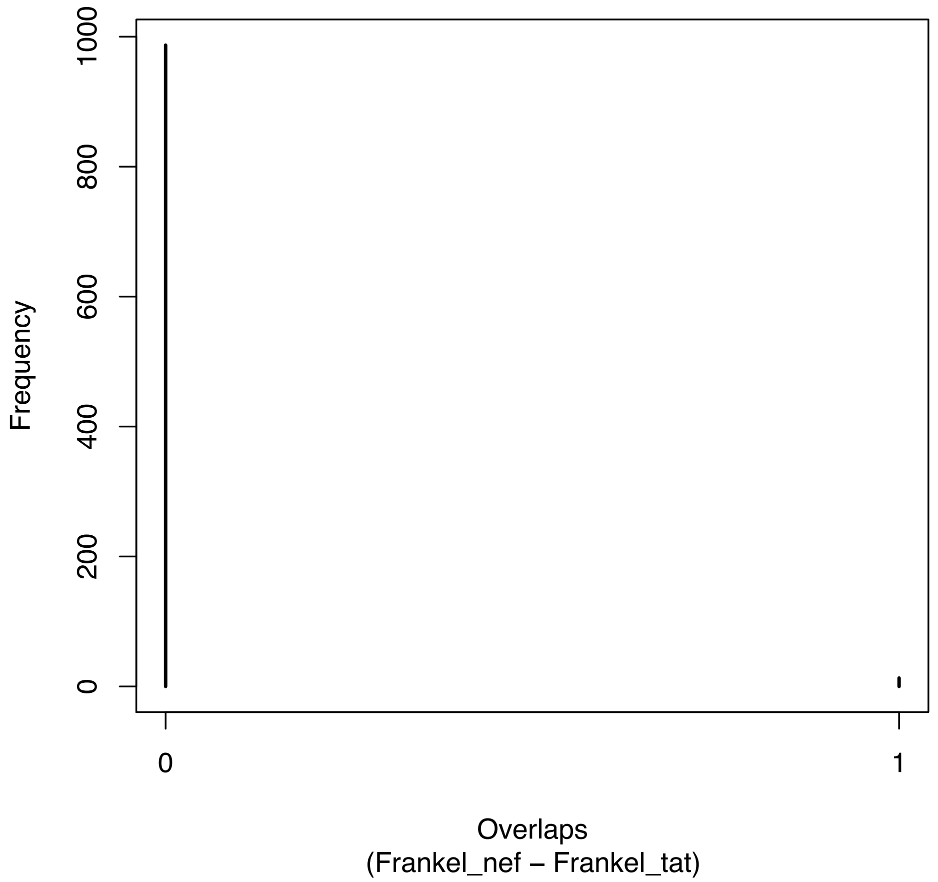

2.47 HARC Nef(6) vs. HARC Rev(56)

Total number of Genes overlapping: 0
Overlapping Genes: ,
Backgrounds Used:

```
  Name                   Size
1 "NCBI_EntrezProtGenes" "25157"
2 "NCBI_EntrezProtGenes" "25157"
```

Hypergeometric p-value: 1
Simulation p-value:
Number of counts that had equal to or greater overlap than (0) in 1000 permutations: 1000 => p-value: 1

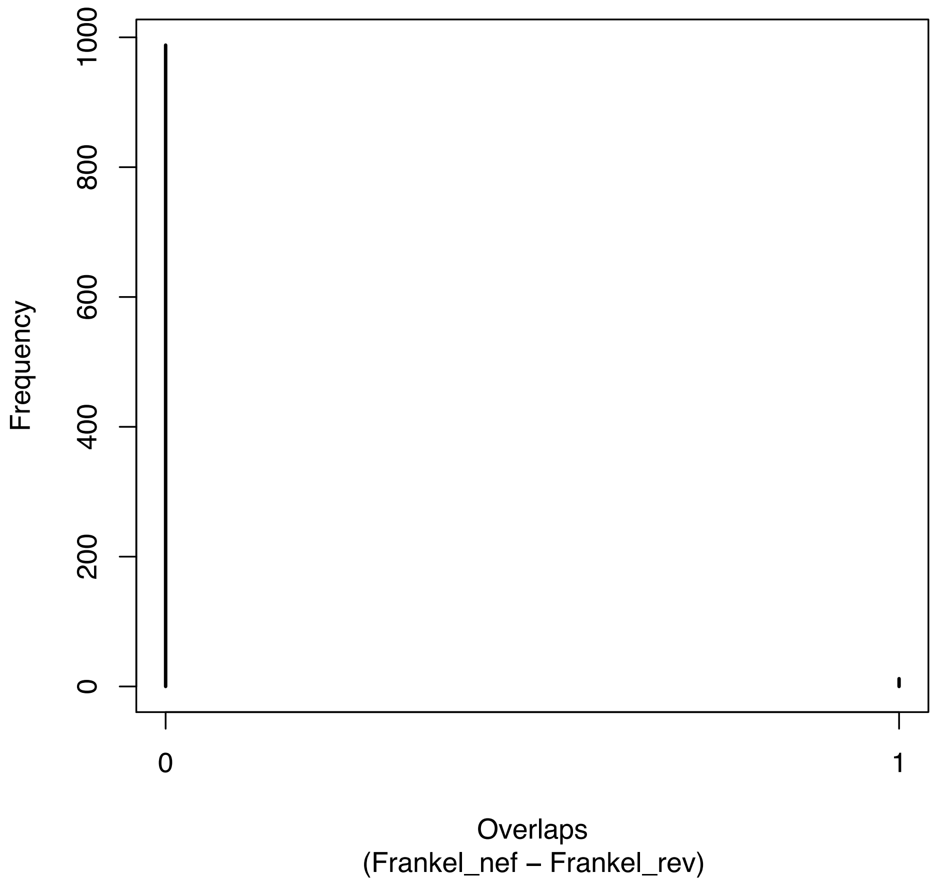

2.48 HARC Nef(6) vs. BIND HIV IN(23)

Total number of Genes overlapping: 0
Overlapping Genes: ,
Backgrounds Used:

```
  Name                   Size
1 "NCBI_EntrezProtGenes" "25157"
2 "NCBI_EntrezProtGenes" "25157"
```

Hypergeometric p-value: 1
Simulation p-value:
Number of counts that had equal to or greater overlap than (0) in 1000 permutations: 1000 => p-value: 1

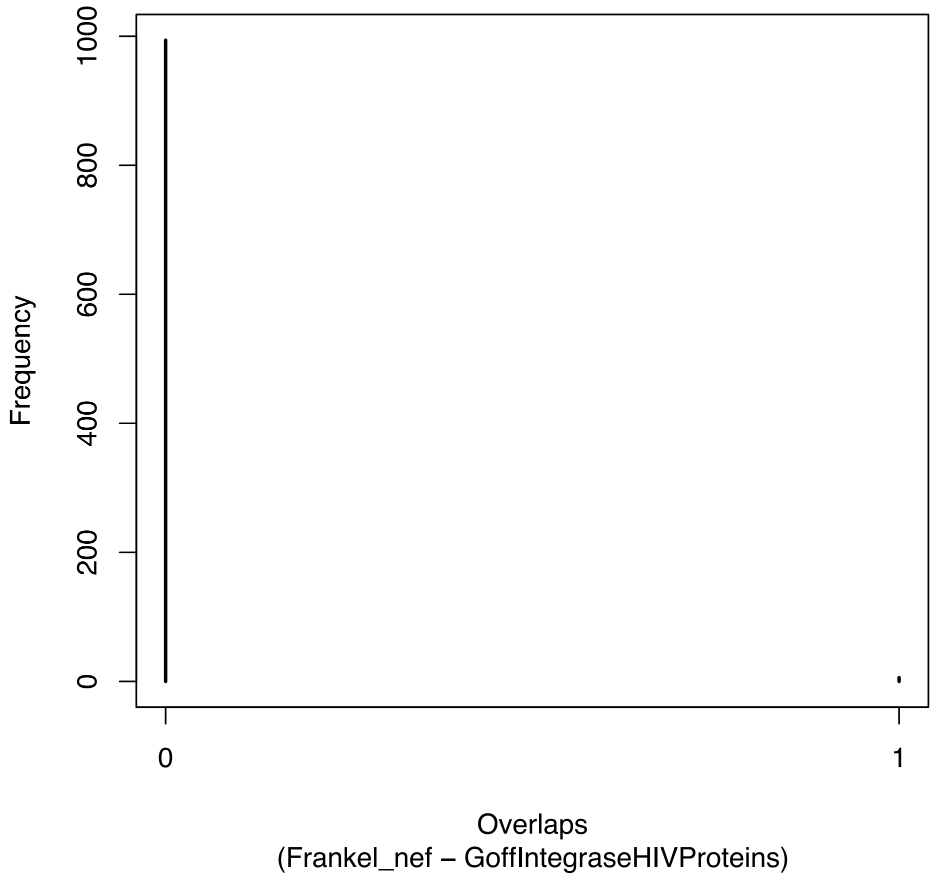

2.49 HARC Nef(6) vs. NCBI Interactions(1434)

Total number of Genes overlapping: 0
Overlapping Genes: ,
Backgrounds Used:

```
  Name                   Size
1 "NCBI_EntrezProtGenes" "25157"
2 "NCBI_EntrezProtGenes" "25157"
```

Hypergeometric p-value: 1
Simulation p-value:
Number of counts that had equal to or greater overlap than (0) in 1000 permutations: 1000 => p-value: 1

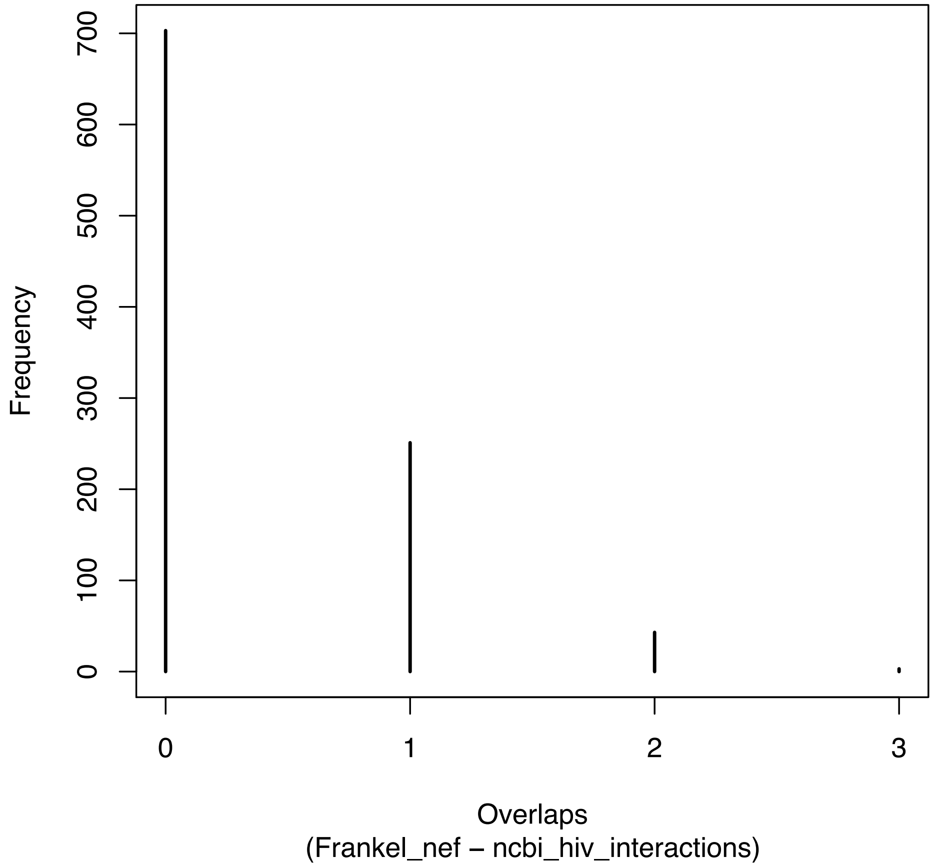

2.50 HARC Nef(6) vs. siRNA Flu Fly(98)

Total number of Genes overlapping: 0
Overlapping Genes: ,
Backgrounds Used:

```
  Name                    Size
1 "NCBI_EntrezProtGenes" "25157"
2 "Fly_RNAi_background"  "19950"
```

Hypergeometric p-value: 1
Simulation p-value:
Number of counts that had equal to or greater overlap than (0) in 1000 permutations: 1000 => p-value: 1

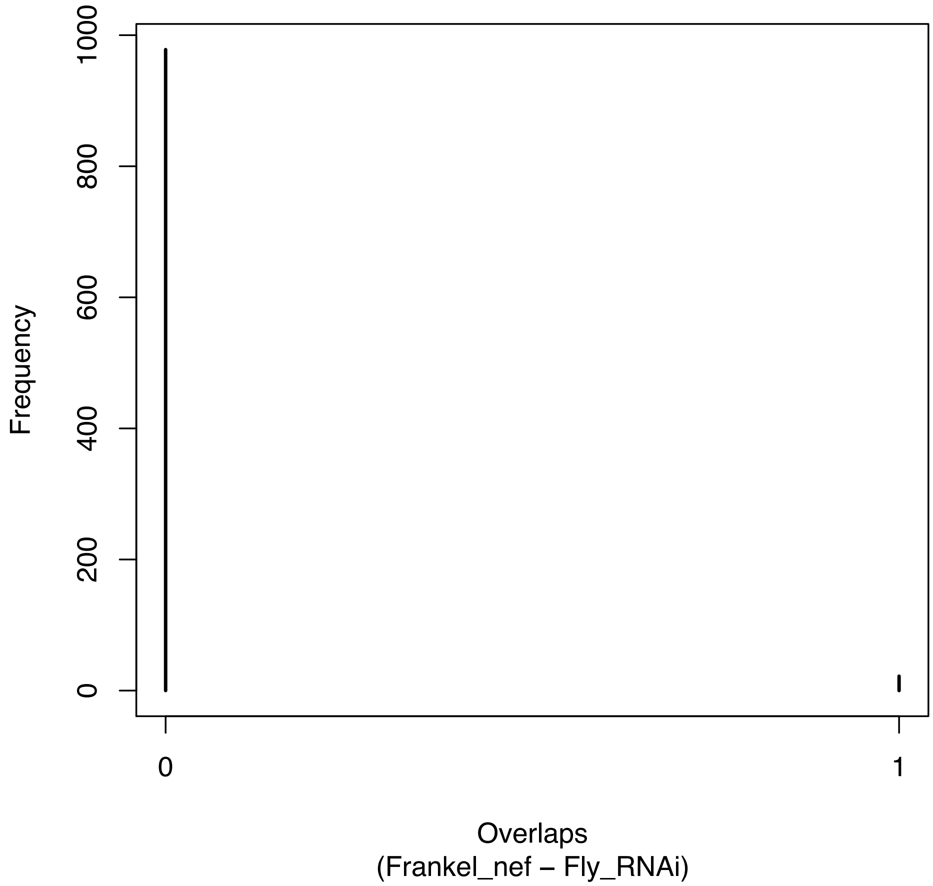

2.51 HARC Nef(6) vs. siRNA WNV(305)

Total number of Genes overlapping: 0
Overlapping Genes: ,
Backgrounds Used:

```
  Name                    Size
1 "NCBI_EntrezProtGenes" "25157"
2 "NCBI_EntrezProtGenes" "25157"
```

Hypergeometric p-value: 1
Simulation p-value:
Number of counts that had equal to or greater overlap than (0) in 1000 permutations: 1000 => p-value: 1

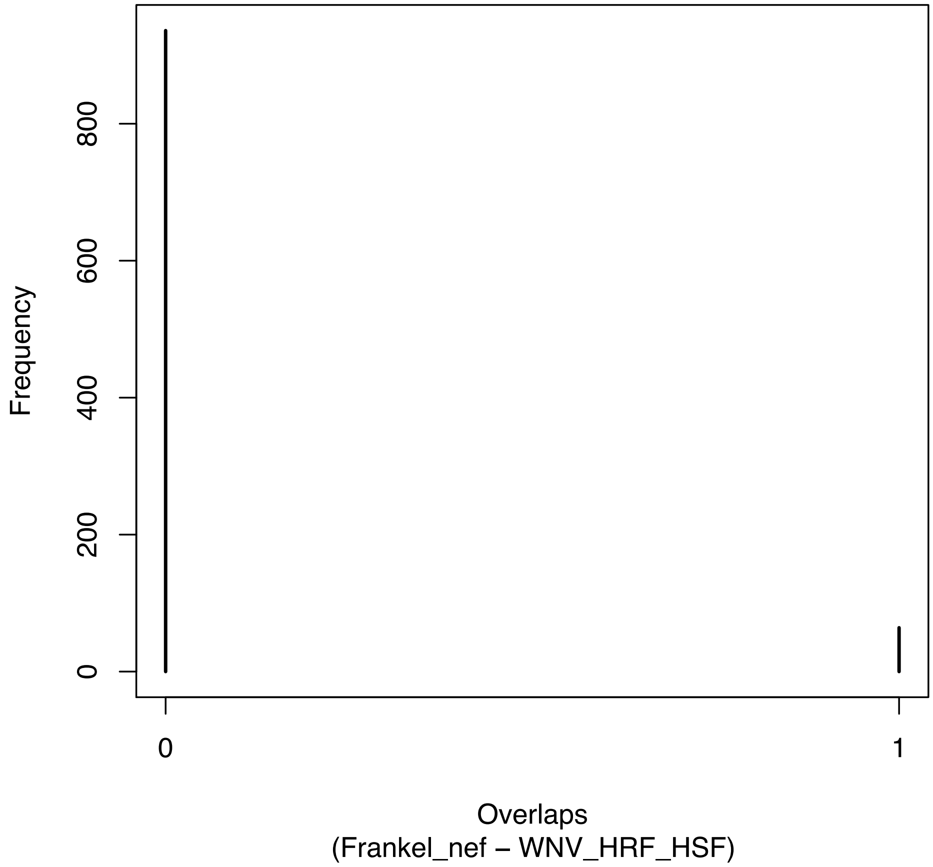

2.52 HARC Tat(69) vs. HARC Rev(56)

Total number of Genes overlapping: 1
Overlapping Genes: DDX3X,
Backgrounds Used:

```
  Name                    Size
1 "NCBI_EntrezProtGenes" "25157"
2 "NCBI_EntrezProtGenes" "25157"
```

Hypergeometric p-value: 0.010378
Simulation p-value:
Number of counts that had equal to or greater overlap than (1) in 1000 permutations: 1000 => p-value: 1

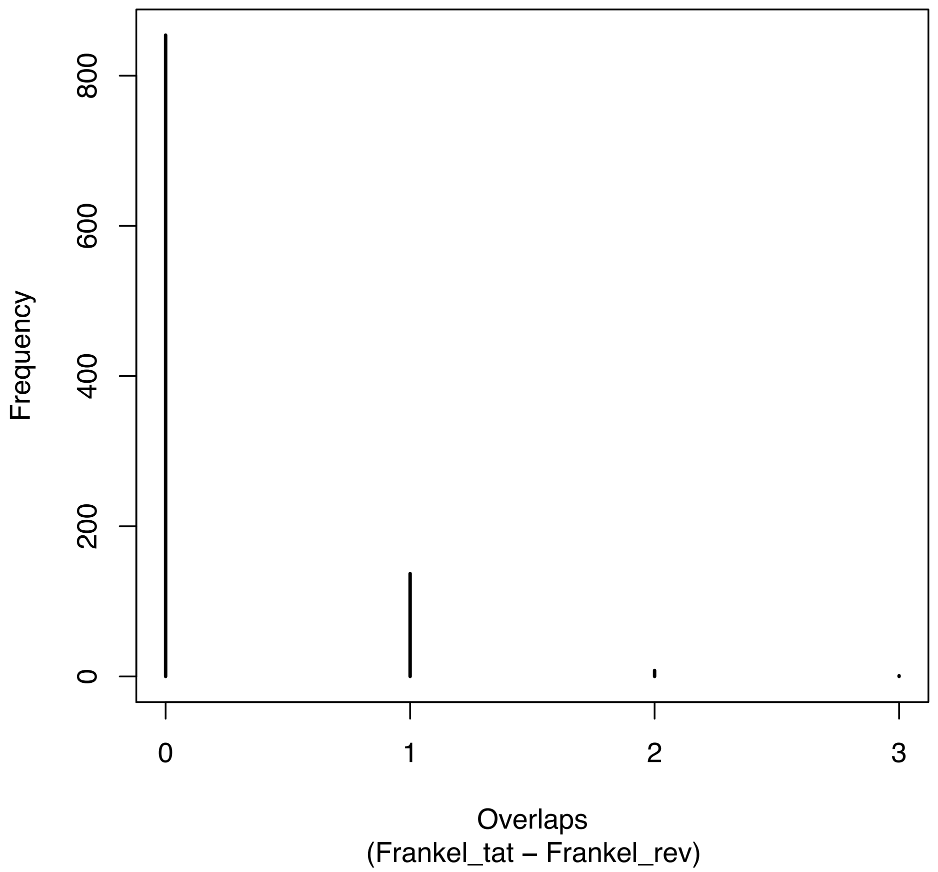

2.53 HARC Tat(69) vs. BIND HIV IN(23)

Total number of Genes overlapping: 0
Overlapping Genes: ,
Backgrounds Used:

```
  Name                    Size
1 "NCBI_EntrezProtGenes" "25157"
2 "NCBI_EntrezProtGenes" "25157"
```

Hypergeometric p-value: 1
Simulation p-value:
Number of counts that had equal to or greater overlap than (0) in 1000 permutations: 1000 => p-value: 1

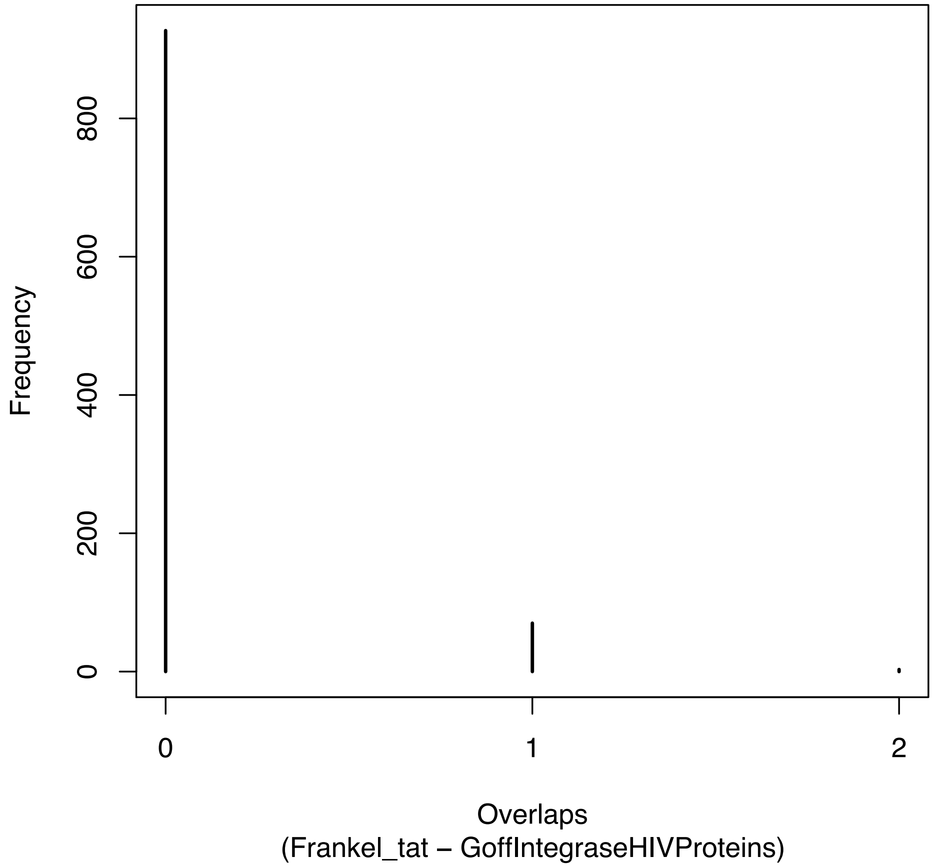

2.54 HARC Tat(69) vs. NCBI Interactions(1434)

Total number of Genes overlapping: 31
Overlapping Genes: CDK9, CCNT1, CCT4, PRKDC, IPO7, PABPC1, CAMK2A, PSME3, CPT1A, DHX9, SPTBN1, ILF3, KPNA1, KPNA6, DDX3X, AIFM1, IPO5, H2AFX, HIST1H2AD, HIST1H2AE, HIST1H2AH, HIST1H2AA, HIST1H2AJ, HIST1H2AC, HIST3H2A, HIST2H2AC, HIST2H2AA3, CAMK2B, ALB, ALPP, KPNA2,
Backgrounds Used:

```
  Name                   Size
1 "NCBI_EntrezProtGenes" "25157"
2 "NCBI_EntrezProtGenes" "25157"
```

Hypergeometric p-value: <0.001
Simulation p-value:
Number of counts that had equal to or greater overlap than (31) in 1000 permutations: 0 => p-value: <0.001

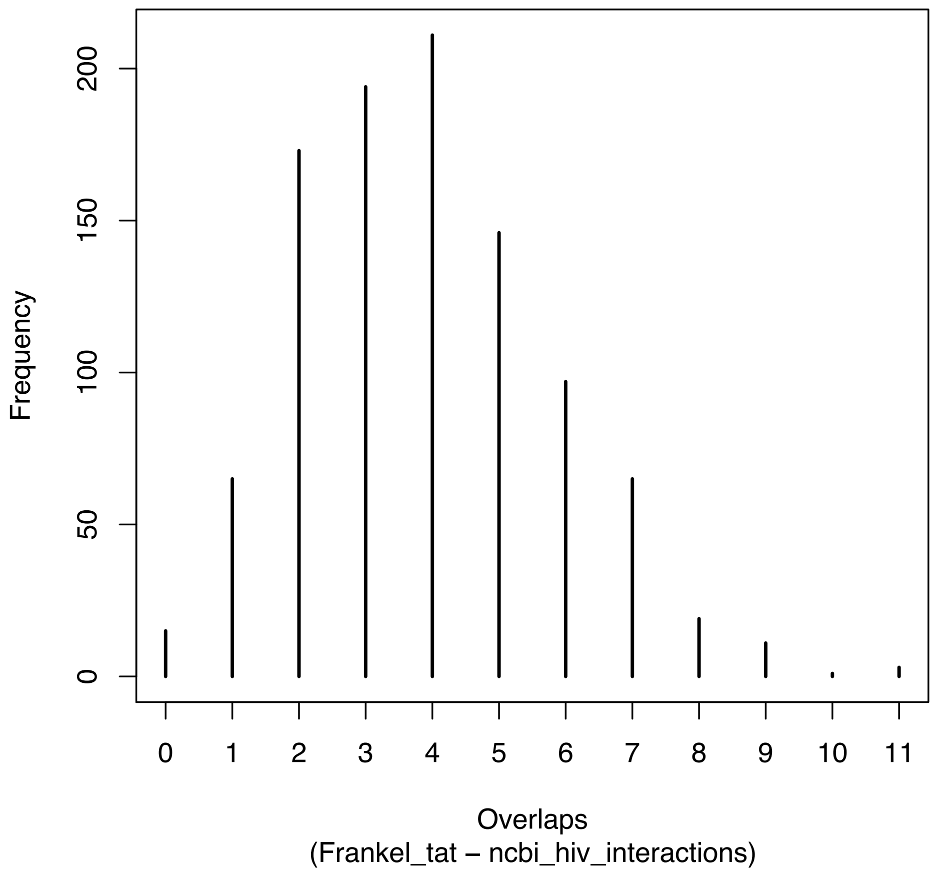

2.55 HARC Tat(69) vs. siRNA Flu Fly(98)

Total number of Genes overlapping: 0
Overlapping Genes: ,
Backgrounds Used:

```
  Name                    Size
1 "NCBI_EntrezProtGenes" "25157"
2 "Fly_RNAi_background"  "19950"
```

Hypergeometric p-value: 1
Simulation p-value:
Number of counts that had equal to or greater overlap than (0) in 1000 permutations: 1000 => p-value: 1

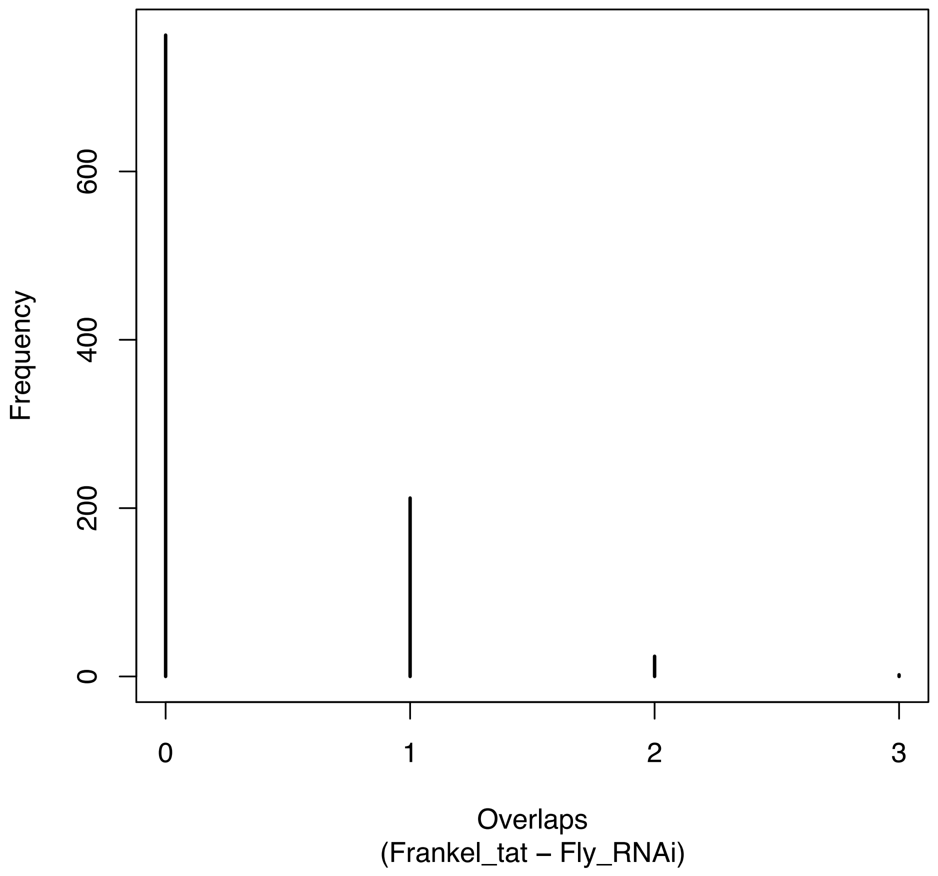

2.56 HARC Tat(69) vs. siRNA WNV(305)

Total number of Genes overlapping: 3
Overlapping Genes: USP11, ANP32B, LAS1L,
Backgrounds Used:

```
  Name                   Size
1 "NCBI_EntrezProtGenes" "25157"
2 "NCBI_EntrezProtGenes" "25157"
```

Hypergeometric p-value: 0.009878738
Simulation p-value:
Number of counts that had equal to or greater overlap than (3) in 1000 permutations: 61 => p-value: 0.061

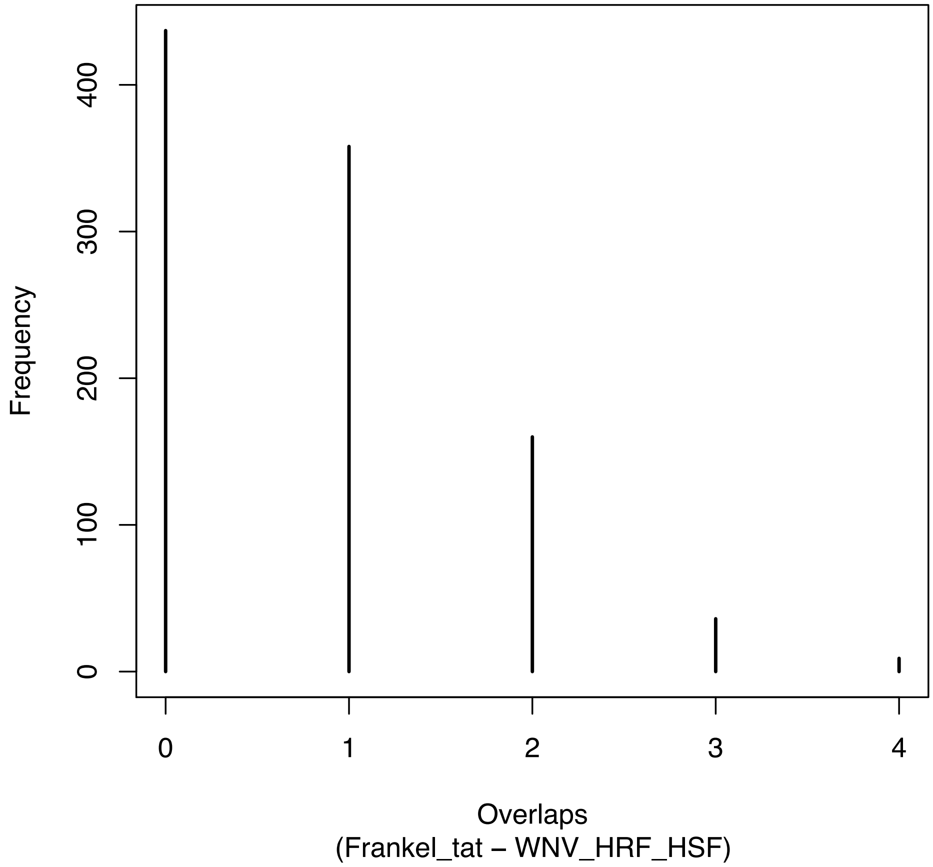

2.57 HARC Rev(56) vs. BIND HIV IN(23)

Total number of Genes overlapping: 1
Overlapping Genes: DDX5,
Backgrounds Used:

```
  Name                    Size
1 "NCBI_EntrezProtGenes" "25157"
2 "NCBI_EntrezProtGenes" "25157"
```

Hypergeometric p-value: 0.001194893
Simulation p-value:
Number of counts that had equal to or greater overlap than (1) in 1000 permutations: 70 => p-value: 0.07

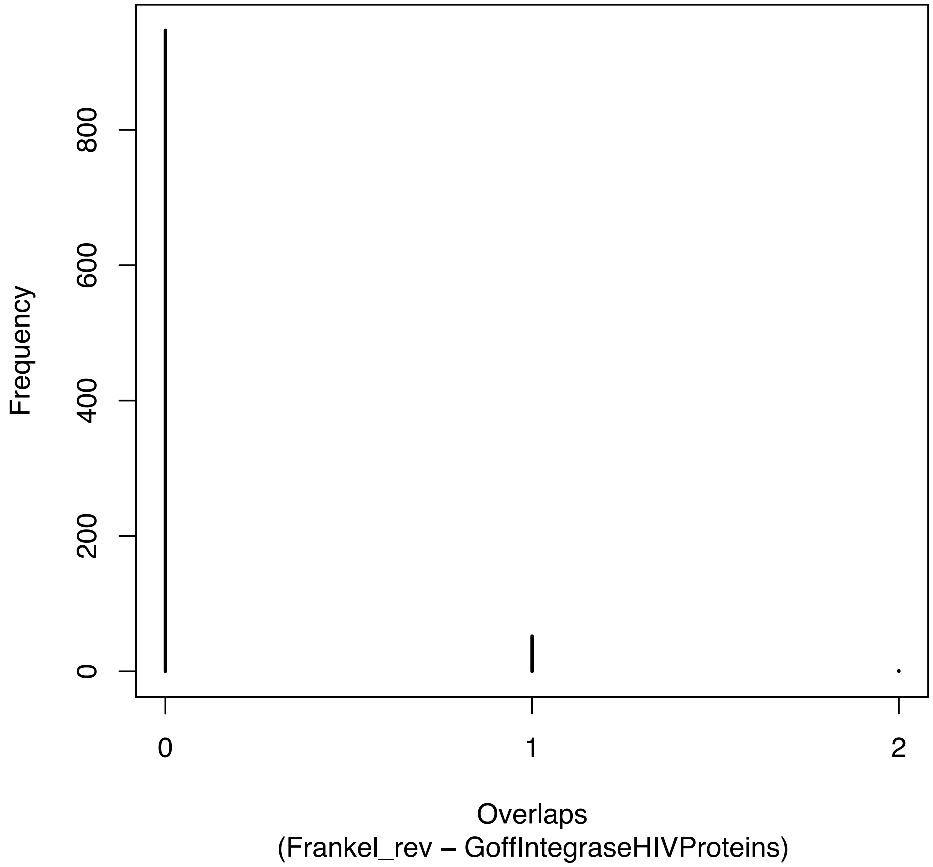

2.58 HARC Rev(56) vs. NCBI Interactions(1434)

Total number of Genes overlapping: 22
Overlapping Genes: TPM2, PPIA, MKI67, YWHAZ, CFL1, HSPA9, NCL, EEF2, HIST1H2BK, UBB, HNRNPK, H2AFV, YWHAQ, GNB2L1, EEF1D, DDX3X, HNRNPA1, SSBP1, STAT3, TRIM21, YWHAE, PTMA,
Backgrounds Used:

```
  Name                    Size
1 "NCBI_EntrezProtGenes" "25157"
2 "NCBI_EntrezProtGenes" "25157"
```

Hypergeometric p-value: <0.001
Simulation p-value:
Number of counts that had equal to or greater overlap than (22) in 1000 permutations: 0 => p-value: <0.001

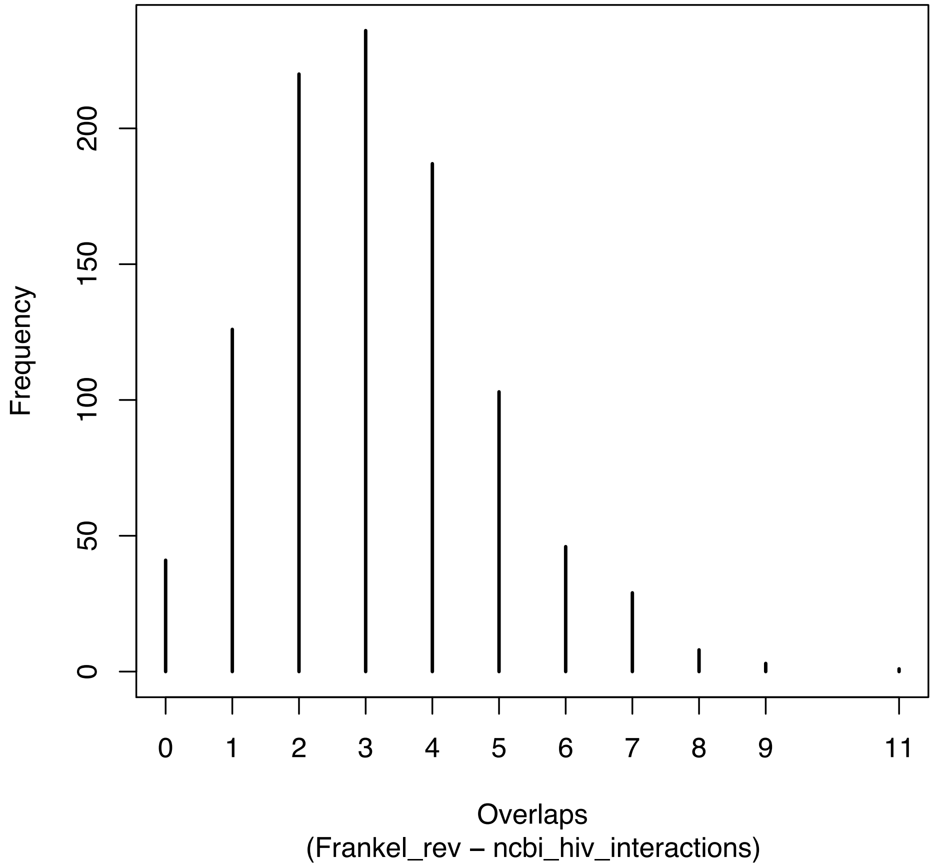

2.59 HARC Rev(56) vs. siRNA Flu Fly(98)

Total number of Genes overlapping: 3
Overlapping Genes: THOC4, VCP, HNRNPA1,
Backgrounds Used:

```
  Name                    Size
1 "NCBI_EntrezProtGenes" "25157"
2 "Fly_RNAi_background"  "19950"
```

Hypergeometric p-value: <0.001
Simulation p-value:
Number of counts that had equal to or greater overlap than (3) in 1000 permutations: 2 => p-value: 0.002

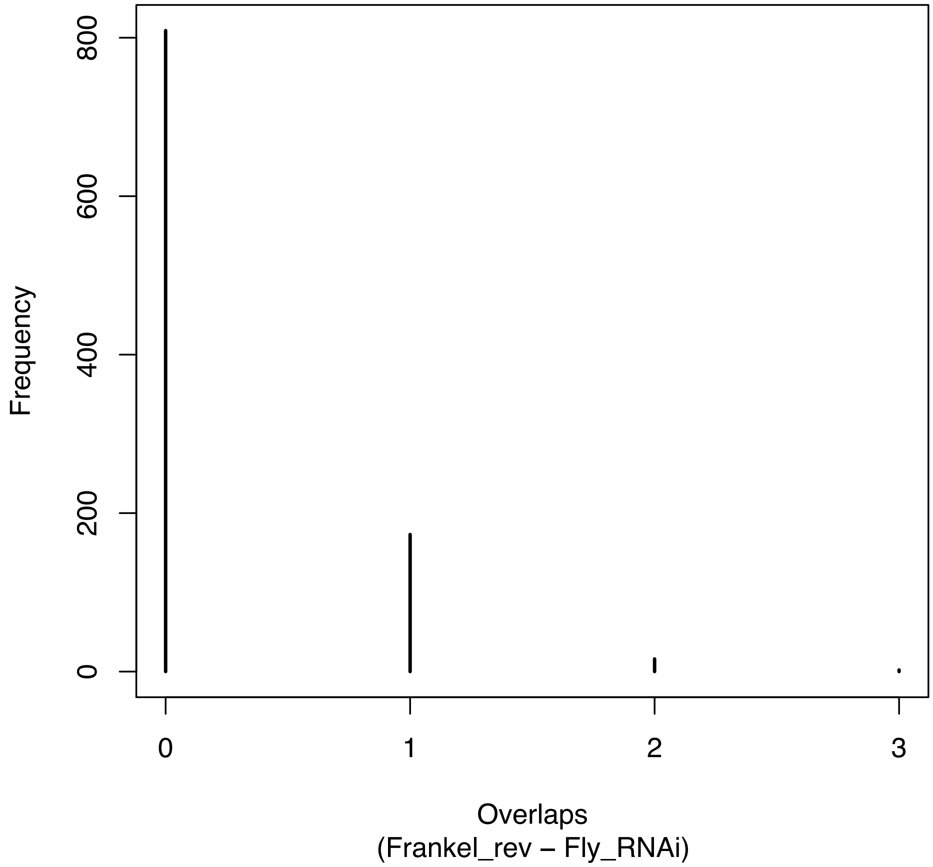

2.60 HARC Rev(56) vs. siRNA WNV(305)

Total number of Genes overlapping: 1
Overlapping Genes: DHX15,
Backgrounds Used:

```
  Name                   Size
1 "NCBI_EntrezProtGenes" "25157"
2 "NCBI_EntrezProtGenes" "25157"
```

Hypergeometric p-value: 0.1477061
Simulation p-value:
Number of counts that had equal to or greater overlap than (1) in 1000 permutations: 481 => p-value: 0.481

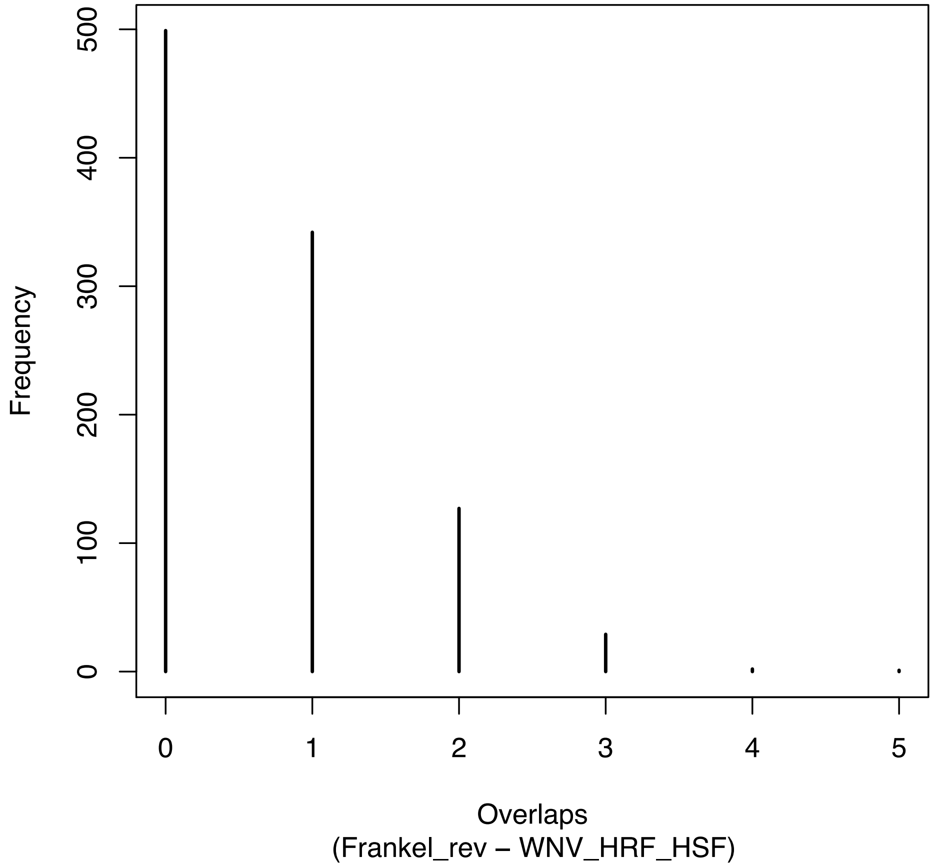

2.61 BIND HIV IN(23) vs. NCBI Interactions(1434)

Total number of Genes overlapping: 5
Overlapping Genes: GTF2E2, RDX, FEN1, SF3B2, XRCC6,
Backgrounds Used:

```
  Name                    Size
1 "NCBI_EntrezProtGenes" "25157"
2 "NCBI_EntrezProtGenes" "25157"
```

Hypergeometric p-value: 0.001479381
Simulation p-value:
Number of counts that had equal to or greater overlap than (5) in 1000 permutations: 9 => p-value: 0.009

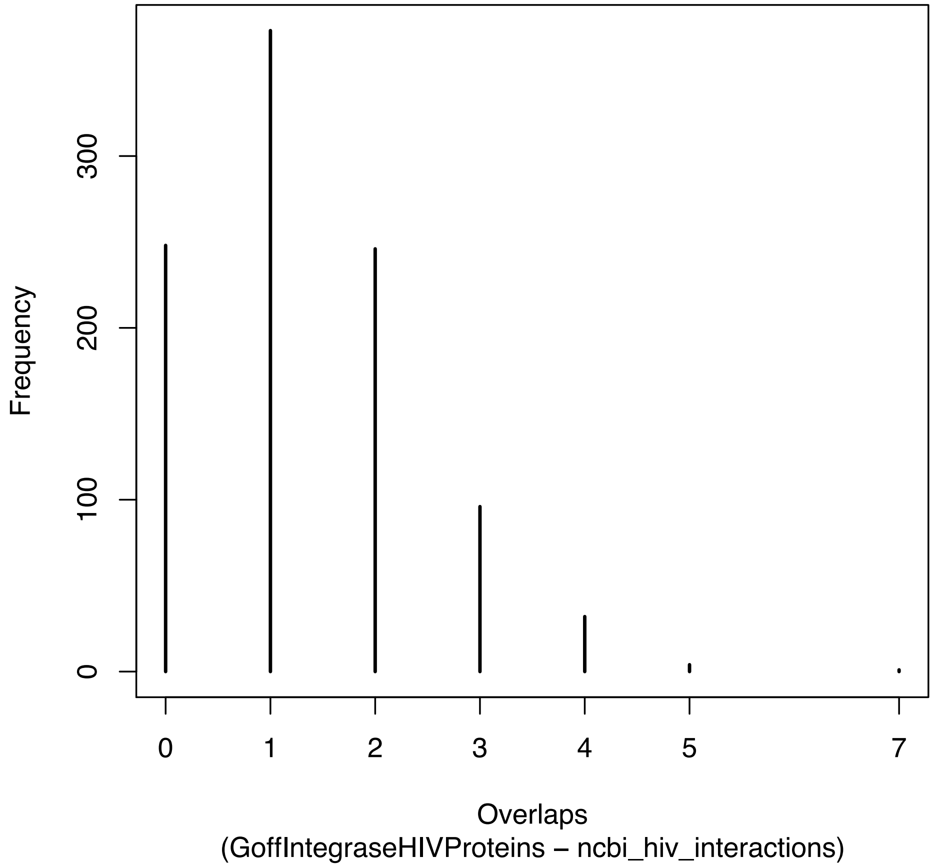

2.62 BIND HIV IN(23) vs. siRNA Flu Fly(98)

Total number of Genes overlapping: 0
Overlapping Genes: ,
Backgrounds Used:

```
  Name                   Size
1 "NCBI_EntrezProtGenes" "25157"
2 "Fly_RNAi_background"  "19950"
```

Hypergeometric p-value: 1
Simulation p-value:
Number of counts that had equal to or greater overlap than (0) in 1000 permutations: 1000 => p-value: 1

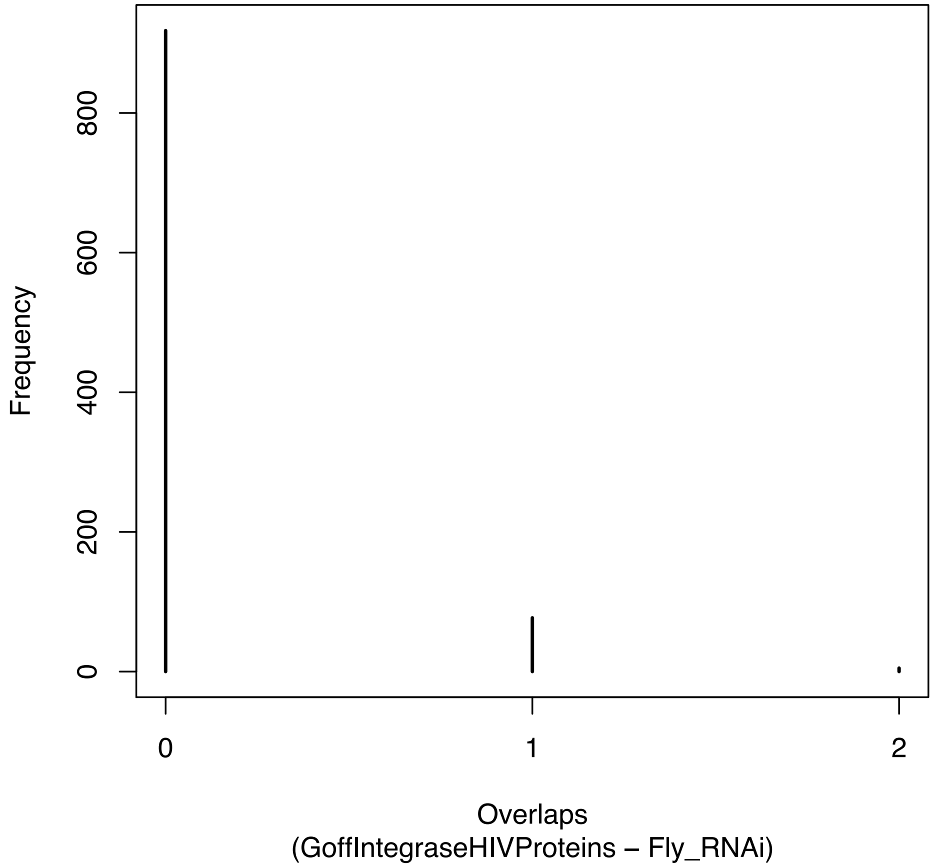

2.63 BIND HIV IN(23) vs. siRNA WNV(305)

Total number of Genes overlapping: 0
Overlapping Genes: ,
Backgrounds Used:

```
  Name                    Size
1 "NCBI_EntrezProtGenes" "25157"
2 "NCBI_EntrezProtGenes" "25157"
```

Hypergeometric p-value: 1
Simulation p-value:
Number of counts that had equal to or greater overlap than (0) in 1000 permutations: 1000 => p-value: 1

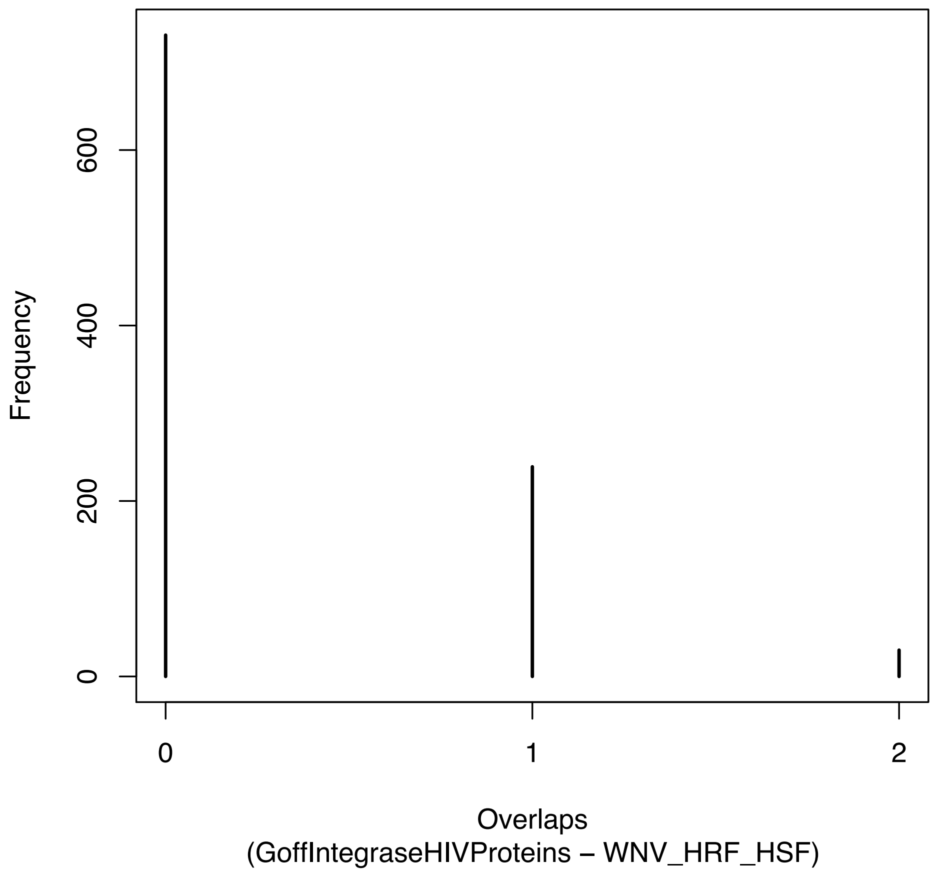

2.64 NCBI Interactions(1434) vs. siRNA Flu Fly(98)

Total number of Genes overlapping: 20
Overlapping Genes: CDC2, HMGCR, HNRNPA1, HSPA5, HSPA8, NUP98, PSMB1, PSMB3, PSMB4, PSMB6, PSMC1, PSMC3, PSMD11, PSMD12, RPL5, SNRPD1, TAF12, HIST3H3, PSMD6, NUP153,
Backgrounds Used:

```
  Name                   Size
1 "NCBI_EntrezProtGenes" "25157"
2 "Fly_RNAi_background"  "19950"
```

Hypergeometric p-value: <0.001
Simulation p-value:
Number of counts that had equal to or greater overlap than (20) in 1000 permutations: 0 => p-value: <0.001

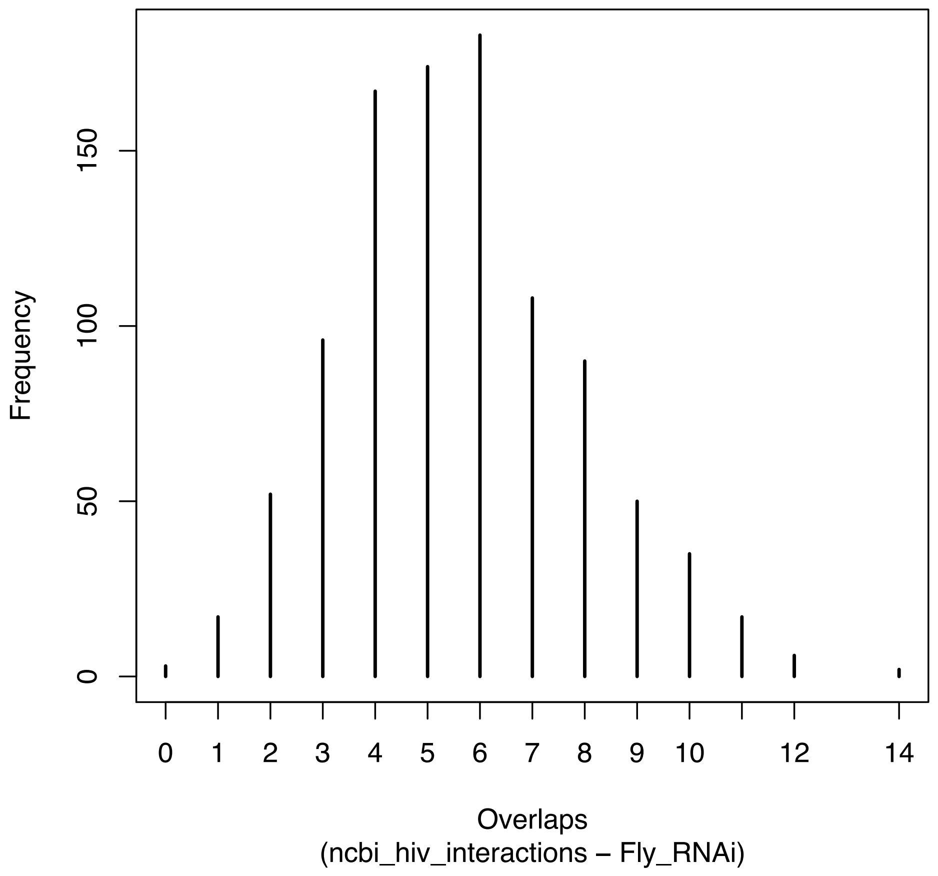

2.65 NCBI Interactions(1434) vs. siRNA WNV(305)

Total number of Genes overlapping: 29
Overlapping Genes: CNP, CSNK2A1, CTSE, DEFA3, PTK2B, B4GALT1, AGFG2, IRF3, MBL2, SCGB2A1, MT2A, PAK1, PDE3B, PHKA1, MAP2K7, PXN, RAD51, ABCE1, SHC1, UBE2I, ZAP70, TUBA1A, AP1M1, PSMF1, TUBB3, TNFSF13B, SPTBN4, PLA2G2F, PLA2G4D,
Backgrounds Used:

```
  Name                    Size
1 "NCBI_EntrezProtGenes" "25157"
2 "NCBI_EntrezProtGenes" "25157"
```

Hypergeometric p-value: 0.0026512
Simulation p-value:
Number of counts that had equal to or greater overlap than (29) in 1000 permutations: 6 => p-value: 0.006

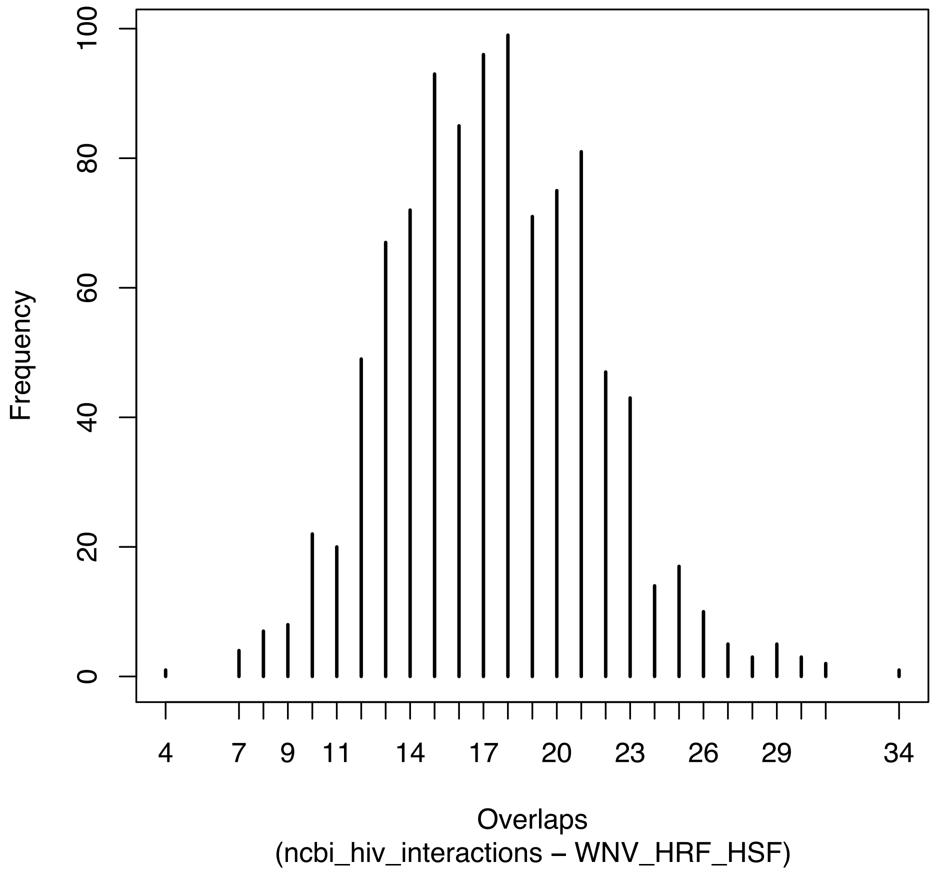

2.66 siRNA Flu Fly(98) vs. siRNA WNV(305)

Total number of Genes overlapping: 2
Overlapping Genes: ATP6V0D1, ATP6V0C,
Backgrounds Used:

```
  Name                    Size
1 "Fly_RNAi_background"  "19950"
2 "NCBI_EntrezProtGenes" "25157"
```

Hypergeometric p-value: 0.1152244
Simulation p-value:
Number of counts that had equal to or greater overlap than (2) in 1000 permutations: 337 => p-value: 0.337

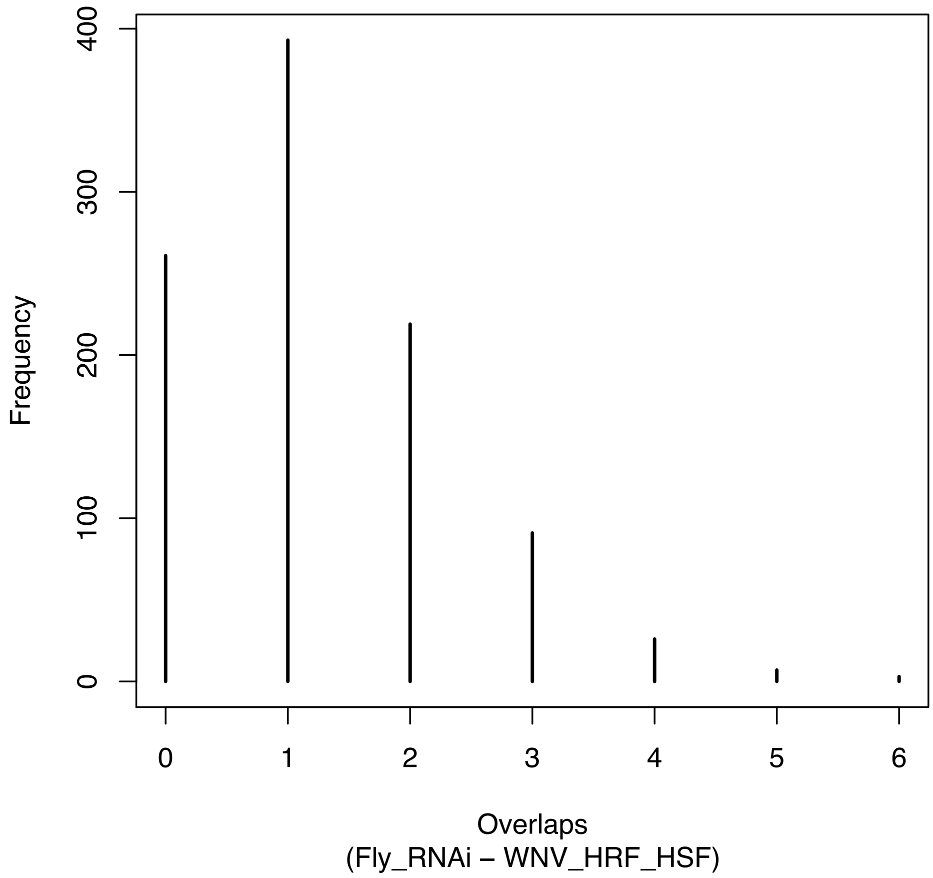

Frequency
0
100
200
300
400
0
1
2
3
4
5
6
Overlaps
(Fly_RNAi – WNV_HRF_HSF)

3 Table of genes called in two or more studies

Table 2: Table of 388 Genes that appear in two or more lists.

**Gene ID**	**Symbol**	**Frequency**	siRNA HIV König	siRNA HIV Brass	siRNA HIV Zhou	SNP HIV Fellay	Particle Associated HIV	HARC Nef	HARC Tat	HARC Rev	BIND HIV IN	NCBI Interactions	siRNA Flu Fly	siRNA WNV	**Druggable HopGrm**	**Druggable Hogenome**	**Exp in CD4**	**Gene Description**
9282	MED14	2	√	√												√	√	mediator complex subunit 14
84675	TRIM55	2	√	√														tripartite motif-containing 55
5903	RANBP2	2	√	√											√	√	√	RAN binding protein 2
4134	MAP4	2	√	√													√	microtubule-associated protein 4
3417	IDH1	2	√	√													√	isocitrate dehydrogenase 1 (NADP+), soluble
23534	TNPO3	2	√	√												√	√	transportin 3
1657	DMXL1	2	√	√													√	Dmx-like 1
4361	MRE11A	2	√		√											√	√	MRE11 meiotic recombination 11 homolog A (S. cerevisiae)
29882	ANAPC2	2	√		√											√	√	anaphase promoting complex subunit 2
256158	HMCN2	2	√		√													hemicentin 2
219541	MED19	2	√		√											√	√	mediator complex subunit 19
156	ADRBK1	2	√		√										√	√	√	adrenergic, beta, receptor kinase 1
5795	PTPRJ	2	√				√								√	√		protein tyrosine phosphatase, receptor type, J
26509	FER1L3	2	√				√										√	fer-1-like 3, myoferlin (C. elegans)
1211	CLTA	2	√				√										√	clathrin, light chain (Lca)
8407	TAGLN2	2	√							√							√	transgelin 2
11127	KIF3A	2	√								√					√	√	kinesin family member 3A
10569	SLU7	2	√								√							SLU7 splicing factor homolog (S. cerevisiae)

Table 2: continued

**Gene ID**	**Symbol**	**Frequency**	siRNA HIV König	siRNA HIV Brass	siRNA HIV Zhou	SNP HIV Fellay	Particle Associated HIV	HARC Nef	HARC Tat	HARC Rev	BIND HIV IN	NCBI Interactions	siRNA Flu Fly	siRNA WNV	**Druggable HopGrm**	**Druggable Hogenome**	**Exp in CD4**	**Gene Description**
8906	AP1G2	2	√									√					√	adaptor-related protein complex 1, gamma 2 subunit
8021	NUP214	2	√									√					√	nucleoporin 214kDa
7994	MYST3	2	√									√				√	√	MYST histone acetyltransferase (monocytic leukemia) 3
7332	UBE2L3	2	√									√					√	ubiquitin-conjugating enzyme E2L 3
6921	TCEB1	2	√									√				√	√	transcription elongation factor B (SIII), polypeptide 1 (15kDa, elongin C)
6634	SNRPD3	2	√									√				√	√	small nuclear ribonucleoprotein D3 polypeptide 18kDa
6627	SNRPA1	2	√									√				√	√	small nuclear ribonucleoprotein polypeptide A'
6626	SNRPA	2	√									√					√	small nuclear ribonucleoprotein polypeptide A
649	BMP1	2	√									√			√	√		bone morphogenetic protein 1
6487	ST3GAL3	2	√									√						ST3 beta-galactoside alpha-2,3-sialyltransferase 3
5705	PSMC5	2	√									√				√	√	proteasome (prosome, macropain) 26S subunit, ATPase, 5
5704	PSMC4	2	√									√				√	√	proteasome (prosome, macropain) 26S subunit, ATPase, 4

Table 2: continued

**Gene ID**	**Symbol**	**Frequency**	siRNA HIV König	siRNA HIV Brass	siRNA HIV Zhou	SNP HIV Fellay	Particle Associated HIV	HARC Nef	HARC Tat	HARC Rev	BIND HIV IN	NCBI Interactions	siRNA Flu Fly	siRNA WNV	**Druggable HopGrm**	**Druggable Hogenome**	**Exp in CD4**	**Gene Description**
5688	PSMA7	2	√									√				√	√	proteasome (prosome, macropain) subunit, alpha type, 7
5686	PSMA5	2	√									√				√	√	proteasome (prosome, macropain) subunit, alpha type, 5
5684	PSMA3	2	√									√				√	√	proteasome (prosome, macropain) subunit, alpha type, 3
5683	PSMA2	2	√									√				√	√	proteasome (prosome, macropain) subunit, alpha type, 2
5682	PSMA1	2	√									√				√	√	proteasome (prosome, macropain) subunit, alpha type, 1
5583	PRKCH	2	√									√			√	√	√	protein kinase C, eta
5529	PPP2R5E	2	√									√				√	√	protein phosphatase 2, regulatory subunit B', epsilon isoform
54476	RNF216	2	√									√					√	ring finger protein 216
5439	POLR2J	2	√									√					√	polymerase (RNA) II (DNA directed) polypeptide J, 13.3kDa
5438	POLR2I	2	√									√					√	polymerase (RNA) II (DNA directed) polypeptide I, 14.5kDa
5432	POLR2C	2	√									√			√	√	√	polymerase (RNA) II (DNA directed) polypeptide C, 33kDa
4904	YBX1	2	√									√					√	Y box binding protein 1

Table 2: continued

**Gene ID**	**Symbol**	**Frequency**	siRNA HIV König	siRNA HIV Brass	siRNA HIV Zhou	SNP HIV Fellay	Particle Associated HIV	HARC Nef	HARC Tat	HARC Rev	BIND HIV IN	NCBI Interactions	siRNA Flu Fly	siRNA WNV	**Druggable HopGrm**	**Druggable Hogenome**	**Exp in CD4**	**Gene Description**
4734	NEDD4	2	√									√				√	√	neural precursor cell expressed, developmentally down-regulated 4
4501	MT1X	2	√									√					√	metallothionein 1X
5430	POLR2A	2	√									√						polymerase (RNA) II (DNA directed) polypeptide A, 220kDa
4126	MANBA	2	√									√					√	mannosidase, beta A, lysosomal
4121	MAN1A1	2	√									√					√	mannosidase, alpha, class 1A, member 1
3735	KARS	2	√									√					√	lysyl-tRNA synthetase
3065	HDAC1	2	√									√				√	√	histone deacetylase 1
2966	GTF2H2	2	√									√				√	√	general transcription factor IIH, polypeptide 2, 44kDa
23636	NUP62	2	√									√				√	√	nucleoporin 62kDa
23193	GANAB	2	√									√			√	√	√	glucosidase, alpha; neutral AB
22938	SNW1	2	√									√				√	√	SNW domain containing 1
22861	NLRP1	2	√									√				√	√	NLR family, pyrin domain containing 1
2033	EP300	2	√									√				√	√	E1A binding protein p300
10923	SUB1	2	√									√					√	SUB1 homolog (S. cerevisiae)
10787	NCKAP1	2	√									√				√	√	NCK-associated protein 1
10728	PTGES3	2	√									√				√	√	prostaglandin E synthase 3 (cytosolic)

Table 2: continued

Gene ID	Symbol	Frequency	siRNA HIV König	siRNA HIV Brass	siRNA HIV Zhou	SNP HIV Fellay	Particle Associated HIV	HARC Nef	HARC Tat	HARC Rev	BIND HIV IN	NCBI Interactions	siRNA Flu Fly	siRNA WNV	**Druggable HopGrm**	**Druggable Hogenome**	**Exp in CD4**	**Gene Description**
10657	KHDRBS1	2	√									√					√	KH domain containing, RNA binding, signal transduction associated 1
9716	AQR	2	√										√				√	aquarius homolog (mouse)
6631	SNRPC	2	√										√				√	small nuclear ribonucleoprotein polypeptide C
4144	MAT2A	2	√										√				√	methionine adenosyltransferase II, alpha
167227	DCP2	2	√										√				√	DCP2 decapping enzyme homolog (S. cerevisiae)
10921	RNPS1	2	√										√				√	RNA binding protein S1, serine-rich domain
10482	NXF1	2	√										√				√	nuclear RNA export factor 1
9253	NUMBL	2	√											√			√	numb homolog (Drosophila)-like
80762	NDFIP1	2	√											√			√	Nedd4 family interacting protein 1
56949	XAB2	2	√											√		√		XPA binding protein 2
10181	RBM5	2	√											√	√	√	√	RNA binding motif protein 5
9364	RAB28	2		√	√											√	√	RAB28, member RAS oncogene family
91074	ANKRD30A	2		√	√										√	√		ankyrin repeat domain 30A
858	CAV2	2		√	√											√	√	caveolin 2
80306	MED28	2		√	√												√	mediator complex subunit 28
79102	RNF26	2		√	√											√		ring finger protein 26
727851	RGPD8	2		√	√												√	RANBP2-like and GRIP domain containing 8

Table 2: continued

**Gene ID**	**Symbol**	**Frequency**	siRNA HIV König	siRNA HIV Brass	siRNA HIV Zhou	SNP HIV Fellay	Particle Associated HIV	HARC Nef	HARC Tat	HARC Rev	BIND HIV IN	NCBI Interactions	siRNA Flu Fly	siRNA WNV	**Druggable HopGrm**	**Druggable Hogenome**	**Exp in CD4**	**Gene Description**
65125	WNK1	2		√	√											√	√	WNK lysine deficient protein kinase 1
29079	MED4	2		√	√											√	√	mediator complex subunit 4
30834	ZNRD1	2		√		√												zinc ribbon domain containing 1
5908	RAP1B	2		√			√									√	√	RAP1B, member of RAS oncogene family
4245	MGAT1	2		√			√									√	√	mannosyl (alpha-1,3-)-glycoprotein beta-1,2-N-acetylglucosaminyltran
3185	HNRNPF	2		√					√								√	heterogeneous nuclear ribonucleoprotein F
1207	CLNS1A	2		√					√								√	chloride channel, nucleotide-sensitive, 1A
10075	HUWE1	2		√						√						√	√	HECT, UBA and WWE domain containing 1
9730	VPRBP	2		√								√					√	Vpr (HIV-1) binding protein
9146	HGS	2		√								√				√	√	hepatocyte growth factor-regulated tyrosine kinase substrate
8487	SIP1	2		√								√				√	√	survival of motor neuron protein interacting protein 1
79902	NUP85	2		√								√					√	nucleoporin 85kDa
7023	TFAP4	2		√								√				√		transcription factor AP-4 (activating enhancer binding protein 4)
6741	SSB	2		√								√					√	Sjogren syndrome antigen B (autoantigen La)

Table 2: continued

**Gene ID**	**Symbol**	**Frequency**	siRNA HIV König	siRNA HIV Brass	siRNA HIV Zhou	SNP HIV Fellay	Particle Associated HIV	HARC Nef	HARC Tat	HARC Rev	BIND HIV IN	NCBI Interactions	siRNA Flu Fly	siRNA WNV	**Druggable HopGrm**	**Druggable Hogenome**	**Exp in CD4**	**Gene Description**
6709	SPTAN1	2		√								√				√	√	spectrin, alpha, non-erythrocytic 1 (alpha-fodrin)
5902	RANBP1	2		√								√					√	RAN binding protein 1
5721	PSME2	2		√								√					√	proteasome (prosome, macropain) activator subunit 2 (PA28 beta)
5520	PPP2R2A	2		√								√				√	√	protein phosphatase 2 (formerly 2A), regulatory subunit B, alpha isoform
4836	NMT1	2		√								√			√	√	√	N-myristoyltransferase 1
4771	NF2	2		√								√				√	√	neurofibromin 2 (merlin)
3431	SP110	2		√								√				√	√	SP110 nuclear body protein
3337	DNAJB1	2		√								√					√	DnaJ (Hsp40) homolog, subfamily B, member 1
3020	H3F3A	2		√								√					√	H3 histone, family 3A
27336	HTATSF1	2		√								√					√	HIV-1 Tat specific factor 1
2648	KAT2A	2		√								√					√	K(lysine) acetyltransferase 2A
23291	FBXW11	2		√								√				√	√	F-box and WD repeat domain containing 11
2071	ERCC3	2		√								√				√	√	excision repair cross-complementing rodent repair deficiency, complementation group 3 (xeroderma pigmentosum group B complementing)

Table 2: continued

**Gene ID**	**Symbol**	**Frequency**	siRNA HIV König	siRNA HIV Brass	siRNA HIV Zhou	SNP HIV Fellay	Particle Associated HIV	HARC Nef	HARC Tat	HARC Rev	BIND HIV IN	NCBI Interactions	siRNA Flu Fly	siRNA WNV	**Druggable HopGrm**	**Druggable Hogenome**	**Exp in CD4**	**Gene Description**
1956	EGFR	2		√								√			√	√	√	epidermal growth factor receptor (erythroblastic leukemia viral (v-erb-b) oncogene homolog, avian)
1950	EGF	2		√								√				√	√	epidermal growth factor (beta-urogastrone)
1173	AP2M1	2		√								√				√	√	adaptor-related protein complex 2, mu 1 subunit
11128	POLR3A	2		√								√						polymerase (RNA) III (DNA directed) polypeptide A, 155kDa
10621	POLR3F	2		√								√					√	polymerase (RNA) III (DNA directed) polypeptide F, 39 kDa
10130	PDIA6	2		√								√			√	√	√	protein disulfide isomerase family A, member 6
8667	EIF3H	2		√									√				√	eukaryotic translation initiation factor 3, subunit H
81876	RAB1B	2		√									√				√	RAB1B, member RAS oncogene family
9853	RUSC2	2		√										√				RUN and SH3 domain containing 2
9098	USP6	2		√										√		√	√	ubiquitin specific peptidase 6 (Tre-2 oncogene)
64784	CRTC3	2		√										√			√	CREB regulated transcription coactivator 3
58485	TRAPPC1	2		√										√				trafficking protein particle complex 1
57626	KLHL1	2		√										√				kelch-like 1 (Drosophila)

Table 2: continued

**Gene ID**	**Symbol**	**Frequency**	siRNA HIV König	siRNA HIV Brass	siRNA HIV Zhou	SNP HIV Fellay	Particle Associated HIV	HARC Nef	HARC Tat	HARC Rev	BIND HIV IN	NCBI Interactions	siRNA Flu Fly	siRNA WNV	**Druggable HopGrm**	**Druggable Hogenome**	**Exp in CD4**	**Gene Description**
23256	SCFD1	2		√										√			√	sec1 family domain containing 1
140730	RIMS4	2		√										√				regulating synaptic membrane exocytosis 4
4277	MICB	2			√	√										√	√	MHC class I polypeptide-related sequence B
4218	RAB8A	2			√		√									√	√	RAB8A, member RAS oncogene family
5899	RALB	2			√		√									√	√	v-ral simian leukemia viral oncogene homolog B (ras related; GTP binding protein)
10963	STIP1	2			√				√								√	stress-induced-phosphoprotein 1
51366	UBR5	2			√					√						√	√	ubiquitin protein ligase E3 component n-recognin 5
29994	BAZ2B	2			√						√					√	√	bromodomain adjacent to zinc finger domain, 2B
1803	DPP4	2			√							√			√	√	√	dipeptidyl-peptidase 4
4318	MMP9	2			√							√			√	√	√	matrix metallopeptidase 9 (gelatinase B, 92kDa gelatinase, 92kDa type IV collagenase)
5046	PCSK6	2			√							√				√	√	proprotein convertase subtilisin/kexin type 6
5151	PDE8A	2			√							√			√	√	√	phosphodiesterase 8A

Table 2: continued

**Gene ID**	**Symbol**	**Frequency**	siRNA HIV König	siRNA HIV Brass	siRNA HIV Zhou	SNP HIV Fellay	Particle Associated HIV	HARC Nef	HARC Tat	HARC Rev	BIND HIV IN	NCBI Interactions	siRNA Flu Fly	siRNA WNV	**Druggable HopGrm**	**Druggable Hogenome**	**Exp in CD4**	**Gene Description**
2957	GTF2A1	2			√							√					√	general transcription factor IIA, 1, 19/37kDa
3552	IL1A	2			√							√				√		interleukin 1, alpha
9020	MAP3K14	2			√							√				√	√	mitogen-activated protein kinase kinase kinase 14
4790	NFKB1	2			√							√			√	√	√	nuclear factor of kappa light polypeptide gene enhancer in B-cells 1
5437	POLR2H	2			√							√					√	polymerase (RNA) II (DNA directed) polypeptide H
5440	POLR2K	2			√							√					√	polymerase (RNA) II (DNA directed) polypeptide K, 7.0kDa
5441	POLR2L	2			√							√					√	polymerase (RNA) II (DNA directed) polypeptide L, 7.6kDa
5710	PSMD4	2			√							√					√	proteasome (prosome, macropain) 26S subunit, non-ATPase, 4
6427	SFRS2	2			√							√					√	splicing factor, arginine/serine-rich 2
1240	CMKLR1	2			√							√			√	√	√	chemokine-like receptor 1
54205	CYCS	2			√							√				√	√	cytochrome c, somatic
2147	F2	2			√							√			√	√		coagulation factor II (thrombin)

Table 2: continued

Gene ID	Symbol	Frequency	siRNA HIV König	siRNA HIV Brass	siRNA HIV Zhou	SNP HIV Fellay	Particle Associated HIV	HARC Nef	HARC Tat	HARC Rev	BIND HIV IN	NCBI Interactions	siRNA Flu Fly	siRNA WNV	**Druggable HopGrm**	**Druggable Hogenome**	**Exp in CD4**	**Gene Description**
2907	GRINA	2			√							√				√	√	glutamate receptor, ionotropic, N-methyl D-aspartate-associated protein 1 (glutamate binding)
3111	HLA-DOA	2			√							√					√	major histocompatibility complex, class II, DO alpha
5105	PCK1	2			√							√				√		phosphoenolpyruvate carboxykinase 1 (soluble)
6382	SDC1	2			√							√				√	√	syndecan 1
51807	TUBA8	2			√							√			√	√	√	tubulin, alpha 8
5756	TWF1	2			√							√				√	√	twinfilin, actin-binding protein, homolog 1 (Drosophila)
7421	VDR	2			√							√			√	√	√	vitamin D (1,25-dihydroxyvitamin D3) receptor
7514	XPO1	2			√							√				√	√	exportin 1 (CRM1 homolog, yeast)
672	BRCA1	2			√							√				√	√	breast cancer 1, early onset
2965	GTF2H1	2			√							√				√	√	general transcription factor IIH, polypeptide 1, 62kDa
5886	RAD23A	2			√							√				√	√	RAD23 homolog A (S. cerevisiae)
9330	GTF3C3	2			√							√					√	general transcription factor IIIC, polypeptide 3, 102kDa
8600	TNFSF11	2			√							√				√	√	tumor necrosis factor (ligand) superfamily, member 11
57576	KIF17	2			√									√		√		kinesin family member 17

Table 2: continued

**Gene ID**	**Symbol**	**Frequency**	siRNA HIV König	siRNA HIV Brass	siRNA HIV Zhou	SNP HIV Fellay	Particle Associated HIV	HARC Nef	HARC Tat	HARC Rev	BIND HIV IN	NCBI Interactions	siRNA Flu Fly	siRNA WNV	**Druggable HopGrm**	**Druggable Hogenome**	**Exp in CD4**	**Gene Description**
23480	SEC61G	2			√									√			√	Sec61 gamma subunit
81494	CFHR5	2			√									√				complement factor H-related 5
23191	CYFIP1	2				√	√										√	cytoplasmic FMR1 interacting protein 1
92421	CHMP4C	2				√						√						chromatin modifying protein 4C
10537	UBD	2				√						√				√		ubiquitin D
23405	DICER1	2				√						√				√	√	dicer 1, ribonuclease type III
3106	HLA-B	2				√						√					√	major histocompatibility complex, class I, B
5413	SEPT5	2				√								√			√	septin 5
201266	SLC39A11	2				√								√				solute carrier family 39 (metal ion transporter), member 11
6281	S100A10	2					√	√									√	S100 calcium binding protein A10
309	ANXA6	2					√	√									√	annexin A6
476	ATP1A1	2					√		√						√	√	√	ATPase, Na+/K+ transporting, alpha 1 polypeptide
5216	PFN1	2					√			√						√	√	profilin 1
5052	PRDX1	2					√			√							√	peroxiredoxin 1
301	ANXA1	2					√			√						√	√	annexin A1
5315	PKM2	2					√			√						√	√	pyruvate kinase, muscle
81	ACTN4	2					√					√				√	√	actinin, alpha 4
121504	HIST4H4	2					√					√					√	histone cluster 4, H4
567	B2M	2					√					√				√	√	beta-2-microglobulin
6383	SDC2	2					√					√				√	√	syndecan 2
8358	HIST1H3B	2					√					√					√	histone cluster 1, H3b
3956	LGALS1	2					√					√				√	√	lectin, galactoside-binding, soluble, 1

Table 2: continued

Gene ID	Symbol	Frequency	siRNA HIV König	siRNA HIV Brass	siRNA HIV Zhou	SNP HIV Fellay	Particle Associated HIV	HARC Nef	HARC Tat	HARC Rev	BIND HIV IN	NCBI Interactions	siRNA Flu Fly	siRNA WNV	**Druggable HopGrm**	**Druggable Hogenome**	**Exp in CD4**	**Gene Description**
391	RHOG	2					√					√					√	ras homolog gene family, member G (rho G)
998	CDC42	2					√					√				√	√	cell division cycle 42 (GTP binding protein, 25kDa)
975	CD81	2					√					√				√	√	CD81 molecule
928	CD9	2					√					√				√	√	CD9 molecule
6386	SDCBP	2					√					√				√	√	syndecan binding protein (syntenin)
8766	RAB11A	2					√					√				√	√	RAB11A, member RAS oncogene family
5901	RAN	2					√					√				√	√	RAN, member RAS oncogene family
3958	LGALS3	2					√					√					√	lectin, galactoside-binding, soluble, 3
7077	TIMP2	2					√					√				√	√	TIMP metallopeptidase inhibitor 2
7529	YWHAB	2					√					√				√	√	tyrosine 3-monooxygenase/trypt[illegible] 5-monooxygenase activation protein, beta polypeptide
8740	TNFSF14	2					√					√				√	√	tumor necrosis factor (ligand) superfamily, member 14
51160	VPS28	2					√					√					√	vacuolar protein sorting 28 homolog (S. cerevisiae)
387	RHOA	2					√					√				√	√	ras homolog gene family, member A
5879	RAC1	2					√					√				√	√	ras-related C3 botulinum toxin substrate 1 (rho family, small GTP binding protein Rac1)
5880	RAC2	2					√					√				√	√	ras-related C3 botulinum toxin substrate 2 (rho family, small GTP binding protein Rac2)

Table 2: continued

Gene ID	Symbol	Frequency	siRNA HIV König	siRNA HIV Brass	siRNA HIV Zhou	SNP HIV Fellay	Particle Associated HIV	HARC Nef	HARC Tat	HARC Rev	BIND HIV IN	NCBI Interactions	siRNA Flu Fly	siRNA WNV	**Druggable HopGrm**	**Druggable Hogenome**	**Exp in CD4**	**Gene Description**
5720	PSME1	2					√					√					√	proteasome (prosome, macropain) activator subunit 1 (PA28 alpha)
1917	EEF1A2	2					√					√				√		eukaryotic translation elongation factor 1 alpha 2
3122	HLA-DRA	2					√					√					√	major histocompatibility complex, class II, DR alpha
302	ANXA2	2					√					√				√	√	annexin A2
348	APOE	2					√					√				√	√	apolipoprotein E
2203	FBP1	2					√					√			√	√	√	fructose-1,6-bisphosphatase 1
9651	PLCH2	2					√					√			√	√		phospholipase C, eta 2
5725	PTBP1	2					√					√				√	√	polypyrimidine tract binding protein 1
60	ACTB	2					√					√					√	actin, beta
59	ACTA2	2					√					√					√	actin, alpha 2, smooth muscle, aorta
3105	HLA-A	2					√					√					√	major histocompatibility complex, class I, A
3732	CD82	2					√					√				√	√	CD82 molecule
7431	VIM	2					√					√					√	vimentin
2023	ENO1	2					√					√				√	√	enolase 1, (alpha)
929	CD14	2					√					√				√	√	CD14 molecule
682	BSG	2					√					√				√	√	basigin (Ok blood group)
7277	TUBA4A	2					√					√			√	√	√	tubulin, alpha 4a
914	CD2	2					√					√				√	√	CD2 molecule
3936	LCP1	2					√					√					√	lymphocyte cytosolic protein 1 (L-plastin)
4478	MSN	2					√					√				√	√	moesin
3306	HSPA2	2					√					√					√	heat shock 70kDa protein 2

Table 2: continued

**Gene ID**	**Symbol**	**Frequency**	siRNA HIV König	siRNA HIV Brass	siRNA HIV Zhou	SNP HIV Fellay	Particle Associated HIV	HARC Nef	HARC Tat	HARC Rev	BIND HIV IN	NCBI Interactions	siRNA Flu Fly	siRNA WNV	**Druggable HopGrm**	**Druggable Hogenome**	**Exp in CD4**	**Gene Description**
3304	HSPA1B	2					√					√					√	heat shock 70kDa protein 1B
965	CD58	2					√					√				√	√	CD58 molecule
3329	HSPD1	2					√					√				√	√	heat shock 60kDa protein 1 (chaperonin)
847	CAT	2					√					√				√	√	catalase
3916	LAMP1	2					√					√					√	lysosomal-associated membrane protein 1
6520	SLC3A2	2					√					√					√	solute carrier family 3 (activators of dibasic and neutral amino acid transport), member 2
3383	ICAM1	2					√					√				√	√	intercellular adhesion molecule 1
2934	GSN	2					√					√				√	√	gelsolin (amyloidosis, Finnish type)
3320	HSP90AA1	2					√					√			√	√	√	heat shock protein 90kDa alpha (cytosolic), class A member 1
6772	STAT1	2					√					√				√	√	signal transducer and activator of transcription 1, 91kDa
5708	PSMD2	2					√					√				√	√	proteasome (prosome, macropain) 26S subunit, non-ATPase, 2
3689	ITGB2	2					√					√			√	√	√	integrin, beta 2 (complement component 3 receptor 3 and 4 subunit)
6693	SPN	2					√					√				√	√	sialophorin
960	CD44	2					√					√				√	√	CD44 molecule (Indian blood group)

Table 2: continued

**Gene ID**	**Symbol**	**Frequency**	siRNA HIV König	siRNA HIV Brass	siRNA HIV Zhou	SNP HIV Fellay	Particle Associated HIV	HARC Nef	HARC Tat	HARC Rev	BIND HIV IN	NCBI Interactions	siRNA Flu Fly	siRNA WNV	**Druggable HopGrm**	**Druggable Hogenome**	**Exp in CD4**	**Gene Description**
3690	ITGB3	2					√					√			√	√	√	integrin, beta 3 (platelet glycoprotein IIIa, antigen CD61)
3675	ITGA3	2					√					√			√	√		integrin, alpha 3 (antigen CD49C, alpha 3 subunit of VLA-3 receptor)
942	CD86	2					√					√				√	√	CD86 molecule
10015	PDCD6IP	2					√					√				√	√	programmed cell death 6 interacting protein
7414	VCL	2					√					√				√	√	vinculin
3684	ITGAM	2					√					√			√	√	√	integrin, alpha M (complement component 3 receptor 3 subunit)
3687	ITGAX	2					√					√			√	√	√	integrin, alpha X (complement component 3 receptor 4 subunit)
3688	ITGB1	2					√					√			√	√	√	integrin, beta 1 (fibronectin receptor, beta polypeptide, antigen CD29 includes MDF2, MSK12)
3678	ITGA5	2					√					√			√	√	√	integrin, alpha 5 (fibronectin receptor, alpha polypeptide)
3685	ITGAV	2					√					√			√	√	√	integrin, alpha V (vitronectin receptor, alpha polypeptide, antigen CD51)
7057	THBS1	2					√					√				√	√	thrombospondin 1
5788	PTPRC	2					√					√			√	√	√	protein tyrosine phosphatase, receptor type, C
3710	ITPR3	2					√					√			√	√	√	inositol 1,4,5-triphosphate receptor, type 3

Table 2: continued

**Gene ID**	**Symbol**	**Frequency**	siRNA HIV König	siRNA HIV Brass	siRNA HIV Zhou	SNP HIV Fellay	Particle Associated HIV	HARC Nef	HARC Tat	HARC Rev	BIND HIV IN	NCBI Interactions	siRNA Flu Fly	siRNA WNV	**Druggable HopGrm**	**Druggable Hogenome**	**Exp in CD4**	**Gene Description**
2335	FN1	2					√					√				√	√	fibronectin 1
1213	CLTC	2					√					√					√	clathrin, heavy chain (Hc)
7094	TLN1	2					√					√				√	√	talin 1
718	C3	2					√					√				√	√	complement component 3
2316	FLNA	2					√					√					√	filamin A, alpha (actin binding protein 280)
5339	PLEC1	2					√					√				√	√	plectin 1, intermediate filament binding protein 500kDa
6217	RPS16	2					√						√				√	ribosomal protein S16
10890	RAB10	2					√						√			√	√	RAB10, member RAS oncogene family
5868	RAB5A	2					√						√			√	√	RAB5A, member RAS oncogene family
5226	PGD	2					√						√				√	phosphogluconate dehydrogenase
64581	CLEC7A	2					√							√		√	√	C-type lectin domain family 7, member A
10134	BCAP31	2					√							√		√	√	B-cell receptor-associated protein 31
1192	CLIC1	2					√							√		√	√	chloride intracellular channel 1
526	ATP6V1B2	2					√							√		√	√	ATPase, H+ transporting, lysosomal 56/58kDa, V1 subunit B2
1025	CDK9	2							√			√			√	√	√	cyclin-dependent kinase 9
10575	CCT4	2							√			√				√	√	chaperonin containing TCP1, subunit 4 (delta)

Table 2: continued

**Gene ID**	**Symbol**	**Frequency**	siRNA HIV König	siRNA HIV Brass	siRNA HIV Zhou	SNP HIV Fellay	Particle Associated HIV	HARC Nef	HARC Tat	HARC Rev	BIND HIV IN	NCBI Interactions	siRNA Flu Fly	siRNA WNV	**Druggable HopGrm**	**Druggable Hogenome**	**Exp in CD4**	**Gene Description**
5591	PRKDC	2							√			√			√	√	√	protein kinase, DNA-activated, catalytic polypeptide
10527	IPO7	2							√			√				√		importin 7
26986	PABPC1	2							√			√					√	poly(A) binding protein, cytoplasmic 1
815	CAMK2A	2							√			√			√	√		calcium/calmodulin-dependent protein kinase (CaM kinase) II alpha
10197	PSME3	2							√			√					√	proteasome (prosome, macropain) activator subunit 3 (PA28 gamma; Ki)
1374	CPT1A	2							√			√			√	√	√	carnitine palmitoyltransferase 1A (liver)
1660	DHX9	2							√			√					√	DEAH (Asp-Glu-Ala-His) box polypeptide 9
3609	ILF3	2							√			√				√	√	interleukin enhancer binding factor 3, 90kDa
3836	KPNA1	2							√			√					√	karyopherin alpha 1 (importin alpha 5)
23633	KPNA6	2							√			√				√	√	karyopherin alpha 6 (importin alpha 7)
9131	AIFM1	2							√			√			√	√	√	apoptosis-inducing factor, mitochondrion-associated, 1
3843	IPO5	2							√			√				√	√	importin 5
3014	H2AFX	2							√			√				√	√	H2A histone family, member X
3013	HIST1H2AD	2							√			√						histone cluster 1, H2ad
3012	HIST1H2AE	2							√			√					√	histone cluster 1, H2ae
85235	HIST1H2AH	2							√			√					√	histone cluster 1, H2ah

Table 2: continued

**Gene ID**	**Symbol**	**Frequency**	siRNA HIV König	siRNA HIV Brass	siRNA HIV Zhou	SNP HIV Fellay	Particle Associated HIV	HARC Nef	HARC Tat	HARC Rev	BIND HIV IN	NCBI Interactions	siRNA Flu Fly	siRNA WNV	**Druggable HopGrm**	**Druggable Hogenome**	**Exp in CD4**	**Gene Description**
221613	HIST1H2AA	2							√			√						histone cluster 1, H2aa
8331	HIST1H2AJ	2							√			√					√	histone cluster 1, H2aj
8334	HIST1H2AC	2							√			√					√	histone cluster 1, H2ac
92815	HIST3H2A	2							√			√					√	histone cluster 3, H2a
8338	HIST2H2AC	2							√			√					√	histone cluster 2, H2ac
8337	HIST2H2AA3	2							√			√					√	histone cluster 2, H2aa3
816	CAMK2B	2							√			√			√	√	√	calcium/calmodulin-dependent protein kinase (CaM kinase) II beta
250	ALPP	2							√			√					√	alkaline phosphatase, placental (Regan isozyme)
3838	KPNA2	2							√			√				√	√	karyopherin alpha 2 (RAG cohort 1, importin alpha 1)
8237	USP11	2							√					√		√	√	ubiquitin specific peptidase 11
10541	ANP32B	2							√					√			√	acidic (leucine-rich) nuclear phosphoprotein 32 family, member B
81887	LAS1L	2							√					√			√	LAS1-like (S. cerevisiae)
1655	DDX5	2								√	√						√	DEAD (Asp-Glu-Ala-Asp) box polypeptide 5
7169	TPM2	2								√		√					√	tropomyosin 2 (beta)
4288	MKI67	2								√		√				√	√	antigen identified by monoclonal antibody Ki-67
4691	NCL	2								√		√				√	√	nucleolin
85236	HIST1H2BK	2								√		√					√	histone cluster 1, H2bk
7314	UBB	2								√		√				√	√	ubiquitin B

Table 2: continued

**Gene ID**	**Symbol**	**Frequency**	siRNA HIV König	siRNA HIV Brass	siRNA HIV Zhou	SNP HIV Fellay	Particle Associated HIV	HARC Nef	HARC Tat	HARC Rev	BIND HIV IN	NCBI Interactions	siRNA Flu Fly	siRNA WNV	**Druggable HopGrm**	**Druggable Hogenome**	**Exp in CD4**	**Gene Description**
3190	HNRNPK	2								√		√					√	heterogeneous nuclear ribonucleoprotein K
94239	H2AFV	2								√		√				√	√	H2A histone family, member V
10971	YWHAQ	2								√		√				√	√	tyrosine 3-monooxygenase/trypto 5-monooxygenase activation protein, theta polypeptide
10399	GNB2L1	2								√		√				√	√	guanine nucleotide binding protein (G protein), beta polypeptide 2-like 1
1936	EEF1D	2								√		√					√	eukaryotic translation elongation factor 1 delta (guanine nucleotide exchange protein)
6742	SSBP1	2								√		√				√	√	single-stranded DNA binding protein 1
6774	STAT3	2								√		√				√	√	signal transducer and activator of transcription 3 (acute-phase response factor)
6737	TRIM21	2								√		√			√	√	√	tripartite motif-containing 21
7531	YWHAE	2								√		√				√	√	tyrosine 3-monooxygenase/trypto 5-monooxygenase activation protein, epsilon polypeptide
5757	PTMA	2								√		√				√	√	prothymosin, alpha
10189	THOC4	2								√			√				√	THO complex 4
2961	GTF2E2	2									√	√					√	general transcription factor IIE, polypeptide 2, beta 34kDa

Table 2: continued

**Gene ID**	**Symbol**	**Frequency**	siRNA HIV König	siRNA HIV Brass	siRNA HIV Zhou	SNP HIV Fellay	Particle Associated HIV	HARC Nef	HARC Tat	HARC Rev	BIND HIV IN	NCBI Interactions	siRNA Flu Fly	siRNA WNV	**Druggable HopGrm**	**Druggable Hogenome**	**Exp in CD4**	**Gene Description**
2237	FEN1	2									√	√				√	√	flap structure-specific endonuclease 1
2547	XRCC6	2									√	√				√	√	X-ray repair complementing defective repair in Chinese hamster cells 6
983	CDC2	2										√	√		√	√	√	cell division cycle 2, G1 to S and G2 to M
3156	HMGCR	2										√	√		√	√	√	3-hydroxy-3-methylglutaryl-Coenzyme A reductase
5689	PSMB1	2										√	√				√	proteasome (prosome, macropain) subunit, beta type, 1
5691	PSMB3	2										√	√			√	√	proteasome (prosome, macropain) subunit, beta type, 3
5692	PSMB4	2										√	√			√	√	proteasome (prosome, macropain) subunit, beta type, 4
5700	PSMC1	2										√	√				√	proteasome (prosome, macropain) 26S subunit, ATPase, 1
5717	PSMD11	2										√	√				√	proteasome (prosome, macropain) 26S subunit, non-ATPase, 11
6125	RPL5	2										√	√				√	ribosomal protein L5
6632	SNRPD1	2										√	√			√	√	small nuclear ribonucleoprotein D1 polypeptide 16kDa

Table 2: continued

**Gene ID**	**Symbol**	**Frequency**	siRNA HIV König	siRNA HIV Brass	siRNA HIV Zhou	SNP HIV Fellay	Particle Associated HIV	HARC Nef	HARC Tat	HARC Rev	BIND HIV IN	NCBI Interactions	siRNA Flu Fly	siRNA WNV	**Druggable HopGrm**	**Druggable Hogenome**	**Exp in CD4**	**Gene Description**
6883	TAF12	2										√	√				√	TAF12 RNA polymerase II, TATA box binding protein (TBP)-associated factor, 20kDa
8290	HIST3H3	2										√	√					histone cluster 3, H3
1457	CSNK2A1	2										√		√	√	√	√	casein kinase 2, alpha 1 polypeptide
1510	CTSE	2										√		√	√	√	√	cathepsin E
1668	DEFA3	2										√		√		√	√	defensin, alpha 3, neutrophil-specific
2185	PTK2B	2										√		√	√	√	√	PTK2B protein tyrosine kinase 2 beta
2683	B4GALT1	2										√		√		√	√	UDP-Gal:betaGlcNAc beta 1,4- galactosyltransferase, polypeptide 1
3268	AGFG2	2										√		√				ArfGAP with FG repeats 2
3661	IRF3	2										√		√		√	√	interferon regulatory factor 3
4153	MBL2	2										√		√		√		mannose-binding lectin (protein C) 2, soluble (opsonic defect)
4246	SCGB2A1	2										√		√				secretoglobin, family 2A, member 1
5058	PAK1	2										√		√	√	√	√	p21 protein (Cdc42/Rac)-activated kinase 1
5255	PHKA1	2										√		√		√		phosphorylase kinase, alpha 1 (muscle)
5609	MAP2K7	2										√		√	√	√	√	mitogen-activated protein kinase kinase 7
5829	PXN	2										√		√		√	√	paxillin

Table 2: continued

**Gene ID**	**Symbol**	**Frequency**	siRNA HIV König	siRNA HIV Brass	siRNA HIV Zhou	SNP HIV Fellay	Particle Associated HIV	HARC Nef	HARC Tat	HARC Rev	BIND HIV IN	NCBI Interactions	siRNA Flu Fly	siRNA WNV	**Druggable HopGrm**	**Druggable Hogenome**	**Exp in CD4**	**Gene Description**
5888	RAD51	2										√		√		√		RAD51 homolog (RecA homolog, E. coli) (S. cerevisiae)
6059	ABCE1	2										√		√		√	√	ATP-binding cassette, sub-family E (OABP), member 1
6464	SHC1	2										√		√		√	√	SHC (Src homology 2 domain containing) transforming protein 1
7329	UBE2I	2										√		√			√	ubiquitin-conjugating enzyme E2I (UBC9 homolog, yeast)
7535	ZAP70	2										√		√	√	√	√	zeta-chain (TCR) associated protein kinase 70kDa
7846	TUBA1A	2										√		√	√	√	√	tubulin, alpha 1a
8907	AP1M1	2										√		√			√	adaptor-related protein complex 1, mu 1 subunit
9491	PSMF1	2										√		√			√	proteasome (prosome, macropain) inhibitor subunit 1 (PI31)
10381	TUBB3	2										√		√	√	√	√	tubulin, beta 3
10673	TNFSF13B	2										√		√		√		tumor necrosis factor (ligand) superfamily, member 13b
57731	SPTBN4	2										√		√				spectrin, beta, non-erythrocytic 4
64600	PLA2G2F	2										√		√	√	√	√	phospholipase A2, group IIF
283748	PLA2G4D	2										√		√				phospholipase A2, group IVD (cytosolic)
9443	MED7	3	√	√	√											√	√	mediator complex subunit 7

Table 2: continued

**Gene ID**	**Symbol**	**Frequency**	siRNA HIV König	siRNA HIV Brass	siRNA HIV Zhou	SNP HIV Fellay	Particle Associated HIV	HARC Nef	HARC Tat	HARC Rev	BIND HIV IN	NCBI Interactions	siRNA Flu Fly	siRNA WNV	**Druggable HopGrm**	**Druggable Hogenome**	**Exp in CD4**	**Gene Description**
9150	CTDP1	3	√	√								√				√	√	CTD (carboxy-terminal domain, RNA polymerase II, polypeptide A) phosphatase, subunit 1
58526	MID1IP1	3	√	√										√			√	MID1 interacting protein 1 (gastrulation specific G12 homolog (zebrafish))
10001	MED6	3	√	√	√											√	√	mediator complex subunit 6
8534	CHST1	3	√		√							√					√	carbohydrate (keratan sulfate Gal-6) sulfotransferase 1
3837	KPNB1	3	√				√					√				√	√	karyopherin (importin) beta 1
1536	CYBB	3	√				√					√				√	√	cytochrome b-245, beta polypeptide
1665	DHX15	3	√							√				√			√	DEAH (Asp-Glu-Ala-His) box polypeptide 15
10992	SF3B2	3	√								√	√					√	splicing factor 3b, subunit 2, 145kDa
9861	PSMD6	3	√									√	√				√	proteasome (prosome, macropain) 26S subunit, non-ATPase, 6
5718	PSMD12	3	√									√	√				√	proteasome (prosome, macropain) 26S subunit, non-ATPase, 12
5702	PSMC3	3	√									√	√			√	√	proteasome (prosome, macropain) 26S subunit, ATPase, 3
5694	PSMB6	3	√									√	√			√	√	proteasome (prosome, macropain) subunit, beta type, 6
4928	NUP98	3	√									√	√			√	√	nucleoporin 98kDa

Table 2: continued

**Gene ID**	**Symbol**	**Frequency**	siRNA HIV König	siRNA HIV Brass	siRNA HIV Zhou	SNP HIV Fellay	Particle Associated HIV	HARC Nef	HARC Tat	HARC Rev	BIND HIV IN	NCBI Interactions	siRNA Flu Fly	siRNA WNV	**Druggable HopGrm**	**Druggable Hogenome**	**Exp in CD4**	**Gene Description**
4502	MT2A	3	√									√		√			√	metallothionein 2A
527	ATP6V0C	3	√										√	√			√	ATPase, H+ transporting, lysosomal 16kDa, V0 subunit c
920	CD4	3		√	√							√				√	√	CD4 molecule
7852	CXCR4	3		√	√							√			√	√	√	chemokine (C-X-C motif) receptor 4
6924	TCEB3	3		√	√							√					√	transcription elongation factor B (SIII), polypeptide 3 (110kDa, elongin A)
3716	JAK1	3		√	√							√			√	√	√	Janus kinase 1 (a protein tyrosine kinase)
207	AKT1	3		√	√							√			√	√	√	v-akt murine thymoma viral oncogene homolog 1
5813	PURA	3		√			√					√					√	purine-rich element binding protein A
5479	PPIB	3		√			√					√				√	√	peptidylprolyl isomerase B (cyclophilin B)
535	ATP6V0A1	3		√			√							√			√	ATPase, H+ transporting, lysosomal V0 subunit a1
375	ARF1	3		√			√					√					√	ADP-ribosylation factor 1
6711	SPTBN1	3		√					√			√					√	spectrin, beta, non-erythrocytic 1
10576	CCT2	3			√		√		√							√	√	chaperonin containing TCP1, subunit 2 (beta)
2923	PDIA3	3			√		√					√			√	√	√	protein disulfide isomerase family A, member 3
506	ATP5B	3			√		√						√			√	√	ATP synthase, H+ transporting, mitochondrial F1 complex, beta polypeptide

Table 2: continued

**Gene ID**	**Symbol**	**Frequency**	siRNA HIV König	siRNA HIV Brass	siRNA HIV Zhou	SNP HIV Fellay	Particle Associated HIV	HARC Nef	HARC Tat	HARC Rev	BIND HIV IN	NCBI Interactions	siRNA Flu Fly	siRNA WNV	**Druggable HopGrm**	**Druggable Hogenome**	**Exp in CD4**	**Gene Description**
7251	TSG101	3			√		√					√				√	√	tumor susceptibility gene 101
3107	HLA-C	3				√	√					√					√	major histocompatibility complex, class I, C
213	ALB	3					√		√			√					√	albumin
5478	PPIA	3					√			√		√					√	peptidylprolyl isomerase A (cyclophilin A)
1072	CFL1	3					√			√		√				√	√	cofilin 1 (non-muscle)
7534	YWHAZ	3					√			√		√					√	tyrosine 3-monooxygenase/trypto[illegible] 5-monooxygenase activation protein, zeta polypeptide
3313	HSPA9	3					√			√		√					√	heat shock 70kDa protein 9 (mortalin)
7415	VCP	3					√			√			√				√	valosin-containing protein
1938	EEF2	3					√			√		√					√	eukaryotic translation elongation factor 2
5962	RDX	3					√				√	√				√	√	radixin
5140	PDE3B	3					√					√		√	√	√	√	phosphodiesterase 3B, cGMP-inhibited
1267	CNP	3					√					√		√			√	2',3'-cyclic nucleotide 3' phosphodiesterase
3309	HSPA5	3					√					√	√			√	√	heat shock 70kDa protein 5 (glucose-regulated protein, 78kDa)
3312	HSPA8	3					√					√	√				√	heat shock 70kDa protein 8
9114	ATP6V0D1	3					√						√	√			√	ATPase, H+ transporting, lysosomal 38kDa, V0 subunit d1

Table 2: continued

**Gene ID**	**Symbol**	**Frequency**	siRNA HIV König	siRNA HIV Brass	siRNA HIV Zhou	SNP HIV Fellay	Particle Associated HIV	HARC Nef	HARC Tat	HARC Rev	BIND HIV IN	NCBI Interactions	siRNA Flu Fly	siRNA WNV	**Druggable HopGrm**	**Druggable Hogenome**	**Exp in CD4**	**Gene Description**
3178	HNRNPA1	3								√		√	√				√	heterogeneous nuclear ribonucleoprotein A1
9972	NUP153	4	√	√								√	√		√	√	√	nucleoporin 153kDa
5970	RELA	4	√	√	√							√			√	√	√	v-rel reticuloendotheliosis viral oncogene homolog A (avian)
904	CCNT1	4		√	√				√			√				√		cyclin T1
1654	DDX3X	5		√	√				√	√		√					√	DEAD (Asp-Glu-Ala-Asp) box polypeptide 3, X-linked

4 Table of union of gene from siRNA screens, annotated for "Druggability" and expression in CD4-positive cells

siRNA screens: siRNA HIV König , siRNA HIV Brass , siRNA HIV Zhou

Table 3: Union table of 842 genes from all siRNA lists above.

**Symbol**	**Druggable HopGrm**	**Druggable Hogenome**	**Exp in CD4**	**Gene Description**
HNRPDL				heterogeneous nuclear ribonucleoprotein D-like
NUP153	√	√	√	nucleoporin 153kDa
RNF10		√		ring finger protein 10
TRIM66				tripartite motif-containing 66
PSMD6			√	proteasome (prosome, macropain) 26S subunit, non-ATPase, 6
DAZAP2				DAZ associated protein 2
PTDSS1				phosphatidylserine synthase 1
TMEM63A				transmembrane protein 63A

Table 3: continued

Symbol	Druggable HopGrm	Druggable Hogenome	Exp in CD4	Gene Description
AQR			√	aquarius homolog (mouse)
CLOCK		√		clock homolog (mouse)
ZNF254	√			zinc finger protein 254
MED7		√	√	mediator complex subunit 7
NTN1		√		netrin 1
DDX23				DEAD (Asp-Glu-Ala-Asp) box polypeptide 23
MED14		√	√	mediator complex subunit 14
NUMBL			√	numb homolog (Drosophila)-like
NDUFAF2				NADH dehydrogenase (ubiquinone) 1 alpha subcomplex, assembly factor 2
CTDP1		√	√	CTD (carboxy-terminal domain, RNA polymerase II, polypeptide A) phosphatase, subunit 1
UMODL1				uromodulin-like 1
TOP3B				topoisomerase (DNA) III beta
AP1G2			√	adaptor-related protein complex 1, gamma 2 subunit
EIF2B5				eukaryotic translation initiation factor 2B, subunit 5 epsilon, 82kDa
PER3		√		period homolog 3 (Drosophila)
MPDZ				multiple PDZ domain protein
CD164				CD164 molecule, sialomucin
CHST1			√	carbohydrate (keratan sulfate Gal-6) sulfotransferase 1
ENC1		√		ectodermal-neural cortex (with BTB-like domain)
RBM17				RNA binding motif protein 17
PRPF38A				PRP38 pre-mRNA processing factor 38 (yeast) domain containing A
ZNF587				zinc finger protein 587
GPT2				glutamic pyruvate transaminase (alanine aminotransferase) 2
TRIM55				tripartite motif-containing 55
ZNF594				zinc finger protein 594
ZGPAT				zinc finger, CCCH-type with G patch domain
PDCD2L				programmed cell death 2-like
GINS4				GINS complex subunit 4 (Sld5 homolog)
TAGLN2			√	transgelin 2
FAM172A				family with sequence similarity 172, member A
PARP9	√			poly (ADP-ribose) polymerase family, member 9
ARID1A		√		AT rich interactive domain 1A (SWI-like)
RBM10	√	√		RNA binding motif protein 10
SFXN3				sideroflexin 3
TRIM56				tripartite motif-containing 56
TRIM7	√	√		tripartite motif-containing 7
TRIM8				tripartite motif-containing 8

Table 3: continued

Symbol	Druggable HopGrm	Druggable Hogenome	Exp in CD4	Gene Description
FXR1		√		fragile X mental retardation, autosomal homolog 1
DUSP16	√	√		dual specificity phosphatase 16
NDFIP1			√	Nedd4 family interacting protein 1
COASY				Coenzyme A synthase
NUP214			√	nucleoporin 214kDa
MUS81				MUS81 endonuclease homolog (S. cerevisiae)
LRRC8E				leucine rich repeat containing 8 family, member E
RMI1				RMI1, RecQ mediated genome instability 1, homolog (S. cerevisiae)
MYST3		√	√	MYST histone acetyltransferase (monocytic leukemia) 3
IQCA1				IQ motif containing with AAA domain 1
CCDC51				coiled-coil domain containing 51
C19orf50				chromosome 19 open reading frame 50
PRNPIP				prion protein interacting protein
CAD	√	√		carbamoyl-phosphate synthetase 2, aspartate transcarbamylase, and dihydroorotase
BTG2				BTG family, member 2
ZYX				zyxin
ZNF148				zinc finger protein 148
VWF		√		von Willebrand factor
C11orf9				chromosome 11 open reading frame 9
UQCRFS1				ubiquinol-cytochrome c reductase, Rieske iron-sulfur polypeptide 1
UMPS	√	√		uridine monophosphate synthetase
SLC35A2				solute carrier family 35 (UDP-galactose transporter), member A2
UBE2L3			√	ubiquitin-conjugating enzyme E2L 3
UBE2H				ubiquitin-conjugating enzyme E2H (UBC8 homolog, yeast)
UBE2B		√		ubiquitin-conjugating enzyme E2B (RAD6 homolog)
TTC3		√		tetratricopeptide repeat domain 3
TPTE	√	√		transmembrane phosphatase with tensin homology
TCF20		√		transcription factor 20 (AR1)
TCEB1		√	√	transcription elongation factor B (SIII), polypeptide 1 (15kDa, elongin C)
STXBP1				syntaxin binding protein 1
SPG7				spastic paraplegia 7 (pure and complicated autosomal recessive)
SNRPD3		√	√	small nuclear ribonucleoprotein D3 polypeptide 18kDa
SNRPC			√	small nuclear ribonucleoprotein polypeptide C
SNRPA1		√	√	small nuclear ribonucleoprotein polypeptide A'
SNRPA			√	small nuclear ribonucleoprotein polypeptide A
SUMO2		√		SMT3 suppressor of mif two 3 homolog 2 (S. cerevisiae)
PYCRL				pyrroline-5-carboxylate reductase-like

Table 3: continued

Symbol	Druggable HopGrm	Druggable Hogenome	Exp in CD4	Gene Description
RANBP17				RAN binding protein 17
BMP1	√	√		bone morphogenetic protein 1
ST3GAL3				ST3 beta-galactoside alpha-2,3-sialyltransferase 3
FAM59A				family with sequence similarity 59, member A
POLR1E				polymerase (RNA) I polypeptide E, 53kDa
CDH22				cadherin-like 22
SFRS6				splicing factor, arginine/serine-rich 6
BLK	√	√		B lymphoid tyrosine kinase
NECAB3				N-terminal EF-hand calcium binding protein 3
ATXN2				ataxin 2
RREB1				ras responsive element binding protein 1
MRPS12		√		mitochondrial ribosomal protein S12
RPL18				ribosomal protein L18
RPL12				ribosomal protein L12
RNH1				ribonuclease/angiogenin inhibitor 1
RELA	√	√	√	v-rel reticuloendotheliosis viral oncogene homolog A (avian)
RANBP2	√	√	√	RAN binding protein 2
RAD21		√		RAD21 homolog (S. pombe)
MID1IP1			√	MID1 interacting protein 1 (gastrulation specific G12 homolog (zebrafish))
RBM25				RNA binding motif protein 25
SCAF1				SR-related CTD-associated factor 1
PVT1				Pvt1 oncogene (non-protein coding)
PTPRN2	√	√		protein tyrosine phosphatase, receptor type, N polypeptide 2
PTPRJ	√	√		protein tyrosine phosphatase, receptor type, J
CALCOCO1				calcium binding and coiled-coil domain 1
MICAL3	√			microtubule associated monoxygenase, calponin and LIM domain containing 3
PSMD12			√	proteasome (prosome, macropain) 26S subunit, non-ATPase, 12
PSMC5		√	√	proteasome (prosome, macropain) 26S subunit, ATPase, 5
PSMC4		√	√	proteasome (prosome, macropain) 26S subunit, ATPase, 4
PSMC3		√	√	proteasome (prosome, macropain) 26S subunit, ATPase, 3
PRDM10				PR domain containing 10
XAB2		√		XPA binding protein 2
PSMB6		√	√	proteasome (prosome, macropain) subunit, beta type, 6
DPYSL5				dihydropyrimidinase-like 5
PSMA7		√	√	proteasome (prosome, macropain) subunit, alpha type, 7
PSMA5		√	√	proteasome (prosome, macropain) subunit, alpha type, 5
PSMA3		√	√	proteasome (prosome, macropain) subunit, alpha type, 3
PSMA2		√	√	proteasome (prosome, macropain) subunit, alpha type, 2
PSMA1		√	√	proteasome (prosome, macropain) subunit, alpha type, 1

Table 3: continued

Symbol	Druggable HopGrm	Druggable Hogenome	Exp in CD4	Gene Description
PSEN2		√		presenilin 2 (Alzheimer disease 4)
IL1F9		√		interleukin 1 family, member 9
USE1				unconventional SNARE in the ER 1 homolog (S. cerevisiae)
PRKCH	√	√	√	protein kinase C, eta
FOXJ2				forkhead box J2
CCAR1				cell division cycle and apoptosis regulator 1
TMEM127				transmembrane protein 127
DOCK10				dedicator of cytokinesis 10
LIN7C		√		lin-7 homolog C (C. elegans)
PPP2R5E		√	√	protein phosphatase 2, regulatory subunit B', epsilon isoform
NUDT11				nudix (nucleoside diphosphate linked moiety X)-type motif 11
TMEM132A				transmembrane protein 132A
PPP1R14D		√		protein phosphatase 1, regulatory (inhibitor) subunit 14D
GATAD2A				GATA zinc finger domain containing 2A
PPP1R12C		√		protein phosphatase 1, regulatory (inhibitor) subunit 12C
TRIM44				tripartite motif-containing 44
FBLIM1				filamin binding LIM protein 1
RNF216			√	ring finger protein 216
POLR2J			√	polymerase (RNA) II (DNA directed) polypeptide J, 13.3kDa
POLR2I			√	polymerase (RNA) II (DNA directed) polypeptide I, 14.5kDa
POLR2C	√	√	√	polymerase (RNA) II (DNA directed) polypeptide C, 33kDa
EXOSC10				exosome component 10
PMM1				phosphomannomutase 1
PLOD2				procollagen-lysine, 2-oxoglutarate 5-dioxygenase 2
PLK1	√	√		polo-like kinase 1 (Drosophila)
UBASH3A		√		ubiquitin associated and SH3 domain containing, A
BCL11A				B-cell CLL/lymphoma 11A (zinc finger protein)
ATP6V0C			√	ATPase, H+ transporting, lysosomal 16kDa, V0 subunit c
PHF2				PHD finger protein 2
PGM1				phosphoglucomutase 1
PFKM		√		phosphofructokinase, muscle
PFKL		√		phosphofructokinase, liver
ARS2				arsenate resistance protein 2
MED15		√		mediator complex subunit 15
ZCCHC17				zinc finger, CCHC domain containing 17
SDF4				stromal cell derived factor 4
AMDHD2				amidohydrolase domain containing 2
MYEF2				myelin expression factor 2
NEUROG3				neurogenin 3
NUP98		√	√	nucleoporin 98kDa
YBX1			√	Y box binding protein 1

Table 3: continued

Symbol	Druggable HopGrm	Druggable Hogenome	Exp in CD4	Gene Description
RPL10A				ribosomal protein L10a
NEDD4		√	√	neural precursor cell expressed, developmentally down-regulated 4
MYO1F				myosin IF
MT2A			√	metallothionein 2A
MT1X			√	metallothionein 1X
POLR2A				polymerase (RNA) II (DNA directed) polypeptide A, 220kDa
EEF1AL7				eukaryotic translation elongation factor 1 alpha-like 7
FAM183A				family with sequence similarity 183, member A
MRE11A		√	√	MRE11 meiotic recombination 11 homolog A (S. cerevisiae)
MPP2		√		membrane protein, palmitoylated 2 (MAGUK p55 subfamily member 2)
MAT2A			√	methionine adenosyltransferase II, alpha
MAP4			√	microtubule-associated protein 4
MANBA			√	mannosidase, beta A, lysosomal
MAN1A1			√	mannosidase, alpha, class 1A, member 1
AAA1				asthma-associated alternatively spliced gene 1
LOC400723				similar to chromosome 20 open reading frame 69
LIMK2		√		LIM domain kinase 2
hCG_1644323				hCG1644323
LOC390876				similar to 60S ribosomal protein L35
KPNB1		√	√	karyopherin (importin) beta 1
KCNJ11		√		potassium inwardly-rectifying channel, subfamily J, member 11
KARS			√	lysyl-tRNA synthetase
LCE3C				late cornified envelope 3C
NACA2				nascent polypeptide-associated complex alpha subunit 2
IDH1			√	isocitrate dehydrogenase 1 (NADP+), soluble
B4GALNT4				beta-1,4-N-acetyl-galactosaminyl transferase 4
XIAP		√		X-linked inhibitor of apoptosis
HNRNPH1				heterogeneous nuclear ribonucleoprotein H1 (H)
HMGCS1	√	√		3-hydroxy-3-methylglutaryl-Coenzyme A synthase 1 (soluble)
HMGB1		√		high-mobility group box 1
HDAC1		√	√	histone deacetylase 1
ANAPC2		√	√	anaphase promoting complex subunit 2
REPIN1				replication initiator 1
GTF2H2		√	√	general transcription factor IIH, polypeptide 2, 44kDa
SAP30BP				SAP30 binding protein
hCG_2028557		√		hCG2028557
C17orf28				chromosome 17 open reading frame 28
NPHP3				nephronophthisis 3 (adolescent)
CHORDC1				cysteine and histidine-rich domain (CHORD)-containing 1

Table 3: continued

Symbol	Druggable HopGrm	Druggable Hogenome	Exp in CD4	Gene Description
INTS6		√		integrator complex subunit 6
FER1L3			√	fer-1-like 3, myoferlin (C. elegans)
OSBPL3				oxysterol binding protein-like 3
CLIP3				CAP-GLY domain containing linker protein 3
C3orf60				chromosome 3 open reading frame 60
AHCTF1				AT hook containing transcription factor 1
SAMM50				sorting and assembly machinery component 50 homolog (S. cerevisiae)
RNF214				ring finger protein 214
HMCN2				hemicentin 2
LOC255275				similar to myeloid-associated differentiation marker
PRPF6		√		PRP6 pre-mRNA processing factor 6 homolog (S. cerevisiae)
TRIM29				tripartite motif-containing 29
NUP62		√	√	nucleoporin 62kDa
FPGS				folylpolyglutamate synthase
TNPO3		√	√	transportin 3
R3HDM1				R3H domain containing 1
SF3B1				splicing factor 3b, subunit 1, 155kDa
ATMIN				ATM interactor
ZC3H7B				zinc finger CCCH-type containing 7B
GANAB	√	√	√	glucosidase, alpha; neutral AB
CEP68				centrosomal protein 68kDa
STAB1	√	√		stabilin 1
SETD1B		√		SET domain containing 1B
NCOA6		√		nuclear receptor coactivator 6
ZNF292				zinc finger protein 292
SPEN		√		spen homolog, transcriptional regulator (Drosophila)
SNW1		√	√	SNW domain containing 1
NLRP1		√	√	NLR family, pyrin domain containing 1
DLGAP4				discs, large (Drosophila) homolog-associated protein 4
ALKBH3		√		alkB, alkylation repair homolog 3 (E. coli)
OTUD1				OTU domain containing 1
MED19		√	√	mediator complex subunit 19
FABP1				fatty acid binding protein 1, liver
ETS2		√		v-ets erythroblastosis virus E26 oncogene homolog 2 (avian)
ERCC5		√		excision repair cross-complementing rodent repair deficiency, complementation group 5
ERCC1		√		excision repair cross-complementing rodent repair deficiency, complementation group 1 (includes overlapping antisense sequence)
EPAS1		√		endothelial PAS domain protein 1
EP300		√	√	E1A binding protein p300

Table 3: continued

Symbol	Druggable HopGrm	Druggable Hogenome	Exp in CD4	Gene Description
DNM2				dynamin 2
DCP2			√	DCP2 decapping enzyme homolog (S. cerevisiae)
DHX15			√	DEAH (Asp-Glu-Ala-His) box polypeptide 15
AES		√		amino-terminal enhancer of split
DMXL1			√	Dmx-like 1
TTLL8				tubulin tyrosine ligase-like family, member 8
C1orf71				chromosome 1 open reading frame 71
NXNL2				nucleoredoxin-like 2
ADRBK1	√	√	√	adrenergic, beta, receptor kinase 1
CYBB		√	√	cytochrome b-245, beta polypeptide
PPM1K				protein phosphatase 1K (PP2C domain containing)
CHADL				chondroadherin-like
ZNF480				zinc finger protein 480
C14orf72				chromosome 14 open reading frame 72
CRYAB				crystallin, alpha B
COL5A1		√		collagen, type V, alpha 1
COX6B2				cytochrome c oxidase subunit VIb polypeptide 2 (testis)
MRPL10				mitochondrial ribosomal protein L10
CANT1				calcium activated nucleotidase 1
AEBP2				AE binding protein 2
CLTA			√	clathrin, light chain (Lca)
OSBPL6				oxysterol binding protein-like 6
CHRNA7	√	√		cholinergic receptor, nicotinic, alpha 7
DUSP12	√	√		dual specificity phosphatase 12
AKAP13		√		A kinase (PRKA) anchor protein 13
WBP4				WW domain binding protein 4 (formin binding protein 21)
NUDT3				nudix (nucleoside diphosphate linked moiety X)-type motif 3
KIF3A		√	√	kinesin family member 3A
TPPP				tubulin polymerization promoting protein
UBE2C		√		ubiquitin-conjugating enzyme E2C
SF3B2			√	splicing factor 3b, subunit 2, 145kDa
LMAN2				lectin, mannose-binding 2
AFG3L2		√		AFG3 ATPase family gene 3-like 2 (yeast)
SUB1			√	SUB1 homolog (S. cerevisiae)
RNPS1			√	RNA binding protein S1, serine-rich domain
ARID5A				AT rich interactive domain 5A (MRF1-like)
NCKAP1		√	√	NCK-associated protein 1
NUP50				nucleoporin 50kDa
PTGES3		√	√	prostaglandin E synthase 3 (cytosolic)
CGREF1				cell growth regulator with EF-hand domain 1

Table 3: continued

Symbol	Druggable HopGrm	Druggable Hogenome	Exp in CD4	Gene Description
KHDRBS1			√	KH domain containing, RNA binding, signal transduction associated 1
CDC42EP3				CDC42 effector protein (Rho GTPase binding) 3
PRPF8		√		PRP8 pre-mRNA processing factor 8 homolog (S. cerevisiae)
SLU7				SLU7 splicing factor homolog (S. cerevisiae)
NXF1			√	nuclear RNA export factor 1
RTN3				reticulon 3
SF3A1		√		splicing factor 3a, subunit 1, 120kDa
TIMM17B				translocase of inner mitochondrial membrane 17 homolog B (yeast)
RBM5	√	√	√	RNA binding motif protein 5
RBM7				RNA binding motif protein 7
PTPRU	√			protein tyrosine phosphatase, receptor type, U
CHAF1A				chromatin assembly factor 1, subunit A (p150)
MED6		√	√	mediator complex subunit 6
NLRX1				NLR family member X1
TOMM70A				translocase of outer mitochondrial membrane 70 homolog A (S. cerevisiae)
RUSC2				RUN and SH3 domain containing 2
RGP1				RGP1 retrograde golgi transport homolog (S. cerevisiae)
ZNF536				zinc finger protein 536
RICS		√		Rho GTPase-activating protein
VPRBP			√	Vpr (HIV-1) binding protein
DEPDC5				DEP domain containing 5
NUP155				nucleoporin 155kDa
NCOR2		√		nuclear receptor co-repressor 2
GOSR2		√		golgi SNAP receptor complex member 2
TM9SF2				transmembrane 9 superfamily member 2
RAB28		√	√	RAB28, member RAS oncogene family
TMEM132C				transmembrane protein 132C
CD4		√	√	CD4 molecule
ALKBH8		√		alkB, alkylation repair homolog 8 (E. coli)
HGS		√	√	hepatocyte growth factor-regulated tyrosine kinase substrate
SESTD1		√		SEC14 and spectrin domains 1
ATG12				ATG12 autophagy related 12 homolog (S. cerevisiae)
ANKRD30A	√	√		ankyrin repeat domain 30A
USP6		√	√	ubiquitin specific peptidase 6 (Tre-2 oncogene)
CCNT1		√		cyclin T1
THAP3				THAP domain containing, apoptosis associated protein 3
HIP1R				huntingtin interacting protein 1 related
PLOD3				procollagen-lysine, 2-oxoglutarate 5-dioxygenase 3

Table 3: continued

Symbol	Druggable HopGrm	Druggable Hogenome	Exp in CD4	Gene Description
ATG16L2				ATG16 autophagy related 16-like 2 (S. cerevisiae)
ST3GAL5				ST3 beta-galactoside alpha-2,3-sialyltransferase 5
DPM1				dolichyl-phosphate mannosyltransferase polypeptide 1, catalytic subunit
EIF3H			√	eukaryotic translation initiation factor 3, subunit H
LY6D				lymphocyte antigen 6 complex, locus D
CAV2		√	√	caveolin 2
IKBKG		√		inhibitor of kappa light polypeptide gene enhancer in B-cells, kinase gamma
PIGY				phosphatidylinositol glycan anchor biosynthesis, class Y
FBXO18		√		F-box protein, helicase, 18
SIP1		√	√	survival of motor neuron protein interacting protein 1
NR0B2	√	√		nuclear receptor subfamily 0, group B, member 2
RGPD5		√		RANBP2-like and GRIP domain containing 5
RAB6C		√		RAB6C, member RAS oncogene family
KBTBD7				kelch repeat and BTB (POZ) domain containing 7
MND1		√		meiotic nuclear divisions 1 homolog (S. cerevisiae)
STARD3NL				STARD3 N-terminal like
TMTC1				transmembrane and tetratricopeptide repeat containing 1
USP26				ubiquitin specific peptidase 26
COG3				component of oligomeric golgi complex 3
DNAL1				dynein, axonemal, light chain 1
DYSF				dysferlin, limb girdle muscular dystrophy 2B (autosomal recessive)
CAPN6	√	√		calpain 6
RAB1B			√	RAB1B, member RAS oncogene family
RNF170		√		ring finger protein 170
TMEM163				transmembrane protein 163
JHDM1D				jumonji C domain containing histone demethylase 1 homolog D (S. cerevisiae)
ZNF436				zinc finger protein 436
MED28			√	mediator complex subunit 28
EFHC2				EF-hand domain (C-terminal) containing 2
NUP85			√	nucleoporin 85kDa
CCDC134				coiled-coil domain containing 134
TUBAL3	√	√		tubulin, alpha-like 3
CSPP1				centrosome and spindle pole associated protein 1
GRTP1				growth hormone regulated TBC protein 1
RNF26		√		ring finger protein 26
CACNG1		√		calcium channel, voltage-dependent, gamma subunit 1
CXCR4	√	√	√	chemokine (C-X-C motif) receptor 4
LAPTM5				lysosomal multispanning membrane protein 5

Table 3: continued

Symbol	Druggable HopGrm	Druggable Hogenome	Exp in CD4	Gene Description
ZNF182				zinc finger protein 182
ZNF12				zinc finger protein 12
WNT1		√		wingless-type MMTV integration site family, member 1
RGPD8			√	RANBP2-like and GRIP domain containing 8
TFE3	√	√		transcription factor binding to IGHM enhancer 3
TFDP2		√		transcription factor Dp-2 (E2F dimerization partner 2)
TFAP4		√		transcription factor AP-4 (activating enhancer binding protein 4)
ZNF354A				zinc finger protein 354A
TCEB3			√	transcription elongation factor B (SIII), polypeptide 3 (110kDa, elongin A)
STX5		√		syntaxin 5
SSB			√	Sjogren syndrome antigen B (autoantigen La)
SPTBN1			√	spectrin, beta, non-erythrocytic 1
SPTAN1		√	√	spectrin, alpha, non-erythrocytic 1 (alpha-fodrin)
SPAST		√		spastin
PLEKHA3				pleckstrin homology domain containing, family A (phosphoinositide binding specific) member 3
WNK1		√	√	WNK lysine deficient protein kinase 1
YTHDC2				YTH domain containing 2
CRTC3			√	CREB regulated transcription coactivator 3
GOLPH3				golgi phosphoprotein 3 (coat-protein)
PRDM14				PR domain containing 14
SEC14L1				SEC14-like 1 (S. cerevisiae)
RTN2				reticulon 2
BCL9		√		B-cell CLL/lymphoma 9
SPCS3				signal peptidase complex subunit 3 homolog (S. cerevisiae)
AGBL5	√			ATP/GTP binding protein-like 5
FKSG2		√		apoptosis inhibitor
RAP1B		√	√	RAP1B, member of RAS oncogene family
RANBP1			√	RAN binding protein 1
RAB6A		√		RAB6A, member RAS oncogene family
RAB2A		√		RAB2A, member RAS oncogene family
TRAPPC1				trafficking protein particle complex 1
PURA			√	purine-rich element binding protein A
FAM5B		√		family with sequence similarity 5, member B
DDX55				DEAD (Asp-Glu-Ala-Asp) box polypeptide 55
PHF12		√		PHD finger protein 12
KLHL1				kelch-like 1 (Drosophila)
ZBTB2				zinc finger and BTB domain containing 2
TMEM181				transmembrane protein 181
TRMT5				TRM5 tRNA methyltransferase 5 homolog (S. cerevisiae)

Table 3: continued

Symbol	Druggable HopGrm	Druggable Hogenome	Exp in CD4	Gene Description
TAOK1	√	√		TAO kinase 1
ZNF512B				zinc finger protein 512B
PSME2			√	proteasome (prosome, macropain) activator subunit 2 (PA28 beta)
THOC2				THO complex 2
NUP107				nucleoporin 107kDa
DHX33				DEAH (Asp-Glu-Ala-His) box polypeptide 33
EXOSC5				exosome component 5
UBQLN4		√		ubiquilin 4
CLDND1				claudin domain containing 1
PRKX	√	√		protein kinase, X-linked
C1orf103				chromosome 1 open reading frame 103
FGD6				FYVE, RhoGEF and PH domain containing 6
NGLY1				N-glycanase 1
NUP133				nucleoporin 133kDa
JMJD2D				jumonji domain containing 2D
NIPSNAP3B				nipsnap homolog 3B (C. elegans)
VPS53				vacuolar protein sorting 53 homolog (S. cerevisiae)
ASXL2				additional sex combs like 2 (Drosophila)
PPP2R2A		√	√	protein phosphatase 2 (formerly 2A), regulatory subunit B, alpha isoform
LRRC8D				leucine rich repeat containing 8 family, member D
HEATR1				HEAT repeat containing 1
ARGLU1				arginine and glutamate rich 1
PPIB		√	√	peptidylprolyl isomerase B (cyclophilin B)
POU1F1		√		POU class 1 homeobox 1
A4GALT		√		alpha 1,4-galactosyltransferase
ATP6V0A1			√	ATPase, H+ transporting, lysosomal V0 subunit a1
PANK1		√		pantothenate kinase 1
PIGH				phosphatidylinositol glycan anchor biosynthesis, class H
RAB6B		√		RAB6B, member RAS oncogene family
LARS	√	√		leucyl-tRNA synthetase
KCNK9		√		potassium channel, subfamily K, member 9
SUV420H1		√		suppressor of variegation 4-20 homolog 1 (Drosophila)
EXOSC3				exosome component 3
NMT1	√	√	√	N-myristoyltransferase 1
NF2		√	√	neurofibromin 2 (merlin)
NDUFB7				NADH dehydrogenase (ubiquinone) 1 beta subcomplex, 7, 18kDa
MOS		√		v-mos Moloney murine sarcoma viral oncogene homolog
MGAT1		√	√	mannosyl (alpha-1,3-)-glycoprotein beta-1,2-N-acetylglucosaminyltransferase
LPL	√	√		lipoprotein lipase

Table 3: continued

Symbol	Druggable HopGrm	Druggable Hogenome	Exp in CD4	Gene Description
LOC402117				similar to CRiM (Cysteine RIch motor neuron protein) homolog family member (crm-1)
FLJ46066				hypothetical gene supported by AK127955
FLJ90680				FLJ90680 protein
FLJ46026				FLJ46026 protein
LCP2				lymphocyte cytosolic protein 2 (SH2 domain containing leukocyte protein of 76kDa)
LOC390530				Putative V-set and immunoglobulin domain-containing-like protein ENSP00000303034
KIF3C		√		kinesin family member 3C
KEL	√	√		Kell blood group, metallo-endopeptidase
C9orf169				chromosome 9 open reading frame 169
LOC375190				hypothetical LOC375190
ARF1			√	ADP-ribosylation factor 1
DKFZp686O24166				hypothetical protein DKFZp686O24166
JAK1	√	√	√	Janus kinase 1 (a protein tyrosine kinase)
ITPKA		√		inositol 1,4,5-trisphosphate 3-kinase A
STT3A				STT3, subunit of the oligosaccharyltransferase complex, homolog A (S. cerevisiae)
IGHMBP2				immunoglobulin mu binding protein 2
SP110		√	√	SP110 nuclear body protein
STAC2				SH3 and cysteine rich domain 2
DNAJB1			√	DnaJ (Hsp40) homolog, subfamily B, member 1
HNRNPF			√	heterogeneous nuclear ribonucleoprotein F
MR1				major histocompatibility complex, class I-related
ZNRD1				zinc ribbon domain containing 1
KCNIP3		√		Kv channel interacting protein 3, calsenilin
H3F3A			√	H3 histone, family 3A
MED4		√	√	mediator complex subunit 4
RAPGEF1		√		Rap guanine nucleotide exchange factor (GEF) 1
LOC285550				hypothetical protein LOC285550
CRIPAK				cysteine-rich PAK1 inhibitor
C3orf56				chromosome 3 open reading frame 56
LOC284214				hypothetical protein LOC284214
GML		√		glycosylphosphatidylinositol anchored molecule like protein
TOR2A				torsin family 2, member A
HTATSF1			√	HIV-1 Tat specific factor 1
PCDH11X				protocadherin 11 X-linked
DIMT1L		√		DIM1 dimethyladenosine transferase 1-like (S. cerevisiae)
LSM3				LSM3 homolog, U6 small nuclear RNA associated (S. cerevisiae)
C2orf25				chromosome 2 open reading frame 25

Table 3: continued

Symbol	Druggable HopGrm	Druggable Hogenome	Exp in CD4	Gene Description
KAT2A			√	K(lysine) acetyltransferase 2A
GCK		√		glucokinase (hexokinase 4)
GBAS				glioblastoma amplified sequence
HIBCH				3-hydroxyisobutyryl-Coenzyme A hydrolase
TIAM2		√		T-cell lymphoma invasion and metastasis 2
RSL1D1				ribosomal L1 domain containing 1
GAPVD1				GTPase activating protein and VPS9 domains 1
LOC26010				viral DNA polymerase-transactivated protein 6
INTS7				integrator complex subunit 7
TRIM58	√	√		tripartite motif-containing 58
COG4				component of oligomeric golgi complex 4
MKRN2		√		makorin ring finger protein 2
KLHDC2				kelch domain containing 2
ETHE1				ethylmalonic encephalopathy 1
PHF3				PHD finger protein 3
PIP5K1C		√		phosphatidylinositol-4-phosphate 5-kinase, type I, gamma
FNTA	√	√		farnesyltransferase, CAAX box, alpha
ARHGEF12				Rho guanine nucleotide exchange factor (GEF) 12
FBXW11		√	√	F-box and WD repeat domain containing 11
NUP160				nucleoporin 160kDa
SCFD1			√	sec1 family domain containing 1
OTUD3				OTU domain containing 3
MDN1				MDN1, midasin homolog (yeast)
SEPT8				septin 8
FLII				flightless I homolog (Drosophila)
WDTC1				WD and tetratricopeptide repeats 1
FBXO21				F-box protein 21
BAHD1				bromo adjacent homology domain containing 1
ANKRD6				ankyrin repeat domain 6
KIAA1012				KIAA1012
COG2		√		component of oligomeric golgi complex 2
FHL3				four and a half LIM domains 3
LNX2		√		ligand of numb-protein X 2
C6orf1				chromosome 6 open reading frame 1
DOK6		√		docking protein 6
ETF1				eukaryotic translation termination factor 1
ERCC3		√	√	excision repair cross-complementing rodent repair deficiency, complementation group 3 (xeroderma pigmentosum group B complementing)
AKT1	√	√	√	v-akt murine thymoma viral oncogene homolog 1
EPS8		√		epidermal growth factor receptor pathway substrate 8

Table 3: continued

Symbol	Druggable HopGrm	Druggable Hogenome	Exp in CD4	Gene Description
CXorf50				chromosome X open reading frame 50
CRTC2				CREB regulated transcription coactivator 2
EGFR	√	√	√	epidermal growth factor receptor (erythroblastic leukemia viral (v-erb-b) oncogene homolog, avian)
EGF		√	√	epidermal growth factor (beta-urogastrone)
EIF2C3		√		eukaryotic translation initiation factor 2C, 3
EDNRA	√	√		endothelin receptor type A
DDX53				DEAD (Asp-Glu-Ala-Asp) box polypeptide 53
TIMM8A		√		translocase of inner mitochondrial membrane 8 homolog A (yeast)
DDX10				DEAD (Asp-Glu-Ala-Asp) box polypeptide 10
DDX3X			√	DEAD (Asp-Glu-Ala-Asp) box polypeptide 3, X-linked
DDOST				dolichyl-diphosphooligosaccharide-protein glycosyltransferase
ZNF791				zinc finger protein 791
IQUB				IQ motif and ubiquitin domain containing
PM20D1				peptidase M20 domain containing 1
LYPD4				LY6/PLAUR domain containing 4
ZNF785				zinc finger protein 785
FAM76B				family with sequence similarity 76, member B
RIMS4				regulating synaptic membrane exocytosis 4
C9orf131				chromosome 9 open reading frame 131
ANKRD43				ankyrin repeat domain 43
C4orf33				chromosome 4 open reading frame 33
RPL32P3				ribosomal protein L32 pseudogene 3
ZNF831				zinc finger protein 831
ARHGEF19				Rho guanine nucleotide exchange factor (GEF) 19
RPTN				repetin
ZNF720				zinc finger protein 720
ANKRD9				ankyrin repeat domain 9
ERP27				endoplasmic reticulum protein 27 kDa
CLNS1A			√	chloride channel, nucleotide-sensitive, 1A
CLN3		√		ceroid-lipofuscinosis, neuronal 3
AP2M1		√	√	adaptor-related protein complex 2, mu 1 subunit
AGAP2				ArfGAP with GTPase domain, ankyrin repeat and PH domain 2
XKR4				XK, Kell blood group complex subunit-related family, member 4
PKD1L2		√		polycystic kidney disease 1-like 2
CARD16	√			caspase recruitment domain family, member 16
GABARAPL2				GABA(A) receptor-associated protein-like 2
SFT2D1				SFT2 domain containing 1
SLC46A1				solute carrier family 46 (folate transporter), member 1
EXOD1				exonuclease domain containing 1
POLR3A				polymerase (RNA) III (DNA directed) polypeptide A, 155kDa

Table 3: continued

Symbol	Druggable HopGrm	Druggable Hogenome	Exp in CD4	Gene Description
PRDM7				PR domain containing 7
TMED2				transmembrane emp24 domain trafficking protein 2
PNRC1		√		proline-rich nuclear receptor coactivator 1
LEFTY1		√		left-right determination factor 1
POLR3F			√	polymerase (RNA) III (DNA directed) polypeptide F, 39 kDa
ARPC1A				actin related protein 2/3 complex, subunit 1A, 41kDa
ATG7				ATG7 autophagy related 7 homolog (S. cerevisiae)
RRAGB				Ras-related GTP binding B
RABEPK		√		Rab9 effector protein with kelch motifs
MPHOSPH6				M-phase phosphoprotein 6
ADAM10	√	√		ADAM metallopeptidase domain 10
PDIA6	√	√	√	protein disulfide isomerase family A, member 6
HUWE1		√	√	HECT, UBA and WWE domain containing 1
PIGK		√		phosphatidylinositol glycan anchor biosynthesis, class K
CKLF		√		chemokine-like factor
MICB		√	√	MHC class I polypeptide-related sequence B
NUDT4		√		nudix (nucleoside diphosphate linked moiety X)-type motif 4
ADAMTS5	√	√		ADAM metallopeptidase with thrombospondin type 1 motif, 5
DPP4	√	√	√	dipeptidyl-peptidase 4
KLK12	√	√		kallikrein-related peptidase 12
MMP13	√	√		matrix metallopeptidase 13 (collagenase 3)
MMP9	√	√	√	matrix metallopeptidase 9 (gelatinase B, 92kDa gelatinase, 92kDa type IV collagenase)
PCSK6		√	√	proprotein convertase subtilisin/kexin type 6
PDE8A	√	√	√	phosphodiesterase 8A
ABTB1				ankyrin repeat and BTB (POZ) domain containing 1
ACACB	√	√		acetyl-Coenzyme A carboxylase beta
ATOX1				ATX1 antioxidant protein 1 homolog (yeast)
BCR		√		breakpoint cluster region
C20orf135				chromosome 20 open reading frame 135
C3orf46				chromosome 3 open reading frame 46
CAMK1D	√	√		calcium/calmodulin-dependent protein kinase ID
CCT2		√	√	chaperonin containing TCP1, subunit 2 (beta)
CHST14				carbohydrate (N-acetylgalactosamine 4-0) sulfotransferase 14
CIB2		√		calcium and integrin binding family member 2
CRIM1	√	√		cysteine rich transmembrane BMP regulator 1 (chordin-like)
DDX60L				DEAD (Asp-Glu-Ala-Asp) box polypeptide 60-like
DTX4		√		deltex 4 homolog (Drosophila)
DVL1		√		dishevelled, dsh homolog 1 (Drosophila)
EVI1		√		ecotropic viral integration site 1
FAM100B				family with sequence similarity 100, member B

Table 3: continued

Symbol	Druggable HopGrm	Druggable Hogenome	Exp in CD4	Gene Description
FEZF2				FEZ family zinc finger 2
FLJ32682				hypothetical protein FLJ32682
GABPB1				GA binding protein transcription factor, beta subunit 1
GCA				grancalcin, EF-hand calcium binding protein
GOLGA9P				golgi autoantigen, golgin subfamily a, 9 pseudogene
GTF2A1			√	general transcription factor IIA, 1, 19/37kDa
HES1		√		hairy and enhancer of split 1, (Drosophila)
IL1A		√		interleukin 1, alpha
ISG20L2				interferon stimulated exonuclease gene 20kDa-like 2
KIAA0922				KIAA0922
KLF2				Kruppel-like factor 2 (lung)
LRRC67				leucine rich repeat containing 67
C5orf45				chromosome 5 open reading frame 45
LSM14B				LSM14B, SCD6 homolog B (S. cerevisiae)
MAP3K14		√	√	mitogen-activated protein kinase kinase kinase 14
MAP3K7	√	√		mitogen-activated protein kinase kinase kinase 7
MED11				mediator complex subunit 11
MED17		√		mediator complex subunit 17
MED26		√		mediator complex subunit 26
MED27				mediator complex subunit 27
MED31				mediator complex subunit 31
MED8		√		mediator complex subunit 8
GABPB2				GA binding protein transcription factor, beta subunit 2
MS4A15		√		membrane-spanning 4-domains, subfamily A, member 15
NBEAL2				neurobeachin-like 2
NEK9	√	√		NIMA (never in mitosis gene a)- related kinase 9
NFKB1	√	√	√	nuclear factor of kappa light polypeptide gene enhancer in B-cells 1
OPN4	√	√		opsin 4
OSBPL7				oxysterol binding protein-like 7
PARVA				parvin, alpha
PCTP				phosphatidylcholine transfer protein
PKN2	√	√		protein kinase N2
PMS2L1	√			postmeiotic segregation increased 2-like 1 pseudogene
POLR2H			√	polymerase (RNA) II (DNA directed) polypeptide H
POLR2K			√	polymerase (RNA) II (DNA directed) polypeptide K, 7.0kDa
POLR2L			√	polymerase (RNA) II (DNA directed) polypeptide L, 7.6kDa
POU6F1				POU class 6 homeobox 1
PSMD4			√	proteasome (prosome, macropain) 26S subunit, non-ATPase, 4
RNF5				ring finger protein 5
S100A1				S100 calcium binding protein A1

Table 3: continued

Symbol	Druggable HopGrm	Druggable Hogenome	Exp in CD4	Gene Description
SBF2				SET binding factor 2
SFRS2			√	splicing factor, arginine/serine-rich 2
SMTN				smoothelin
SRPK3		√		SFRS protein kinase 3
TLK2	√	√		tousled-like kinase 2
TMED10P				transmembrane emp24-like trafficking protein 10 (yeast) pseudogene
TNFRSF4				tumor necrosis factor receptor superfamily, member 4
UAP1				UDP-N-acteylglucosamine pyrophosphorylase 1
UBR5		√	√	ubiquitin protein ligase E3 component n-recognin 5
UPF3B				UPF3 regulator of nonsense transcripts homolog B (yeast)
ZBED5				zinc finger, BED-type containing 5
PARVB				parvin, beta
PDIA3	√	√	√	protein disulfide isomerase family A, member 3
CTSZ	√	√		cathepsin Z
SENP5		√		SUMO1/sentrin specific peptidase 5
USP39				ubiquitin specific peptidase 39
ZMPSTE24	√	√		zinc metallopeptidase (STE24 homolog, S. cerevisiae)
A26B1				ANKRD26-like family B, member 1
AASS		√		aminoadipate-semialdehyde synthase
ACADSB				acyl-Coenzyme A dehydrogenase, short/branched chain
GPR182	√	√		G protein-coupled receptor 182
ALG14				asparagine-linked glycosylation 14 homolog (S. cerevisiae)
ATP5B		√	√	ATP synthase, H+ transporting, mitochondrial F1 complex, beta polypeptide
BAT3		√		HLA-B associated transcript 3
BCLAF1				BCL2-associated transcription factor 1
BICD2				bicaudal D homolog 2 (Drosophila)
BMP2K		√		BMP2 inducible kinase
CA2	√	√		carbonic anhydrase II
CAMKK2	√	√		calcium/calmodulin-dependent protein kinase kinase 2, beta
CD97	√	√		CD97 molecule
CDC40				cell division cycle 40 homolog (S. cerevisiae)
CMKLR1	√	√	√	chemokine-like receptor 1
CYCS		√	√	cytochrome c, somatic
CYP3A4	√	√		cytochrome P450, family 3, subfamily A, polypeptide 4
CYP46A1	√	√		cytochrome P450, family 46, subfamily A, polypeptide 1
DAPK2	√	√		death-associated protein kinase 2
DCBLD1				discoidin, CUB and LCCL domain containing 1
DDO				D-aspartate oxidase
DDX50				DEAD (Asp-Glu-Ala-Asp) box polypeptide 50

Table 3: continued

Symbol	Druggable HopGrm	Druggable Hogenome	Exp in CD4	Gene Description
DLST				dihydrolipoamide S-succinyltransferase (E2 component of 2-oxo-glutarate complex)
DNAJA2				DnaJ (Hsp40) homolog, subfamily A, member 2
LPAR2	√	√		lysophosphatidic acid receptor 2
ERN2		√		endoplasmic reticulum to nucleus signaling 2
ETV3				ets variant 3
F2	√	√		coagulation factor II (thrombin)
FLJ40125				hypothetical protein FLJ40125
GABARAP		√		GABA(A) receptor-associated protein
GH2		√		growth hormone 2
GM2A		√		GM2 ganglioside activator
GPSN2				glycoprotein, synaptic 2
GRINA		√	√	glutamate receptor, ionotropic, N-methyl D-aspartate-associated protein 1 (glutamate binding)
HLA-DOA			√	major histocompatibility complex, class II, DO alpha
HSPC072				HSPC072 protein
KIF17		√		kinesin family member 17
KIF2B		√		kinesin family member 2B
KLRC2				killer cell lectin-like receptor subfamily C, member 2
KRBA2				KRAB-A domain containing 2
LRRC14				leucine rich repeat containing 14
MAP3K9	√	√		mitogen-activated protein kinase kinase kinase 9
MARK1	√	√		MAP/microtubule affinity-regulating kinase 1
MED20				mediator complex subunit 20
MRPL23		√		mitochondrial ribosomal protein L23
MRPL44				mitochondrial ribosomal protein L44
MUC5B				mucin 5B, oligomeric mucus/gel-forming
MYLK2	√	√		myosin light chain kinase 2
NDUFA10	√			NADH dehydrogenase (ubiquinone) 1 alpha subcomplex, 10, 42kDa
NDUFA6				NADH dehydrogenase (ubiquinone) 1 alpha subcomplex, 6, 14kDa
NDUFS6				NADH dehydrogenase (ubiquinone) Fe-S protein 6, 13kDa (NADH-coenzyme Q reductase)
NDUFS7				NADH dehydrogenase (ubiquinone) Fe-S protein 7, 20kDa (NADH-coenzyme Q reductase)
NEIL3	√	√		nei endonuclease VIII-like 3 (E. coli)
PCGF1				polycomb group ring finger 1
PCK1		√		phosphoenolpyruvate carboxykinase 1 (soluble)
PDSS1				prenyl (decaprenyl) diphosphate synthase, subunit 1
PELI1				pellino homolog 1 (Drosophila)
PGK2		√		phosphoglycerate kinase 2

Table 3: continued

Symbol	Druggable HopGrm	Druggable Hogenome	Exp in CD4	Gene Description
PGRMC2		√		progesterone receptor membrane component 2
PKLR		√		pyruvate kinase, liver and RBC
PRKAA1	√	√		protein kinase, AMP-activated, alpha 1 catalytic subunit
PTHR1	√	√		parathyroid hormone receptor 1
RAB1C		√		RAB1C, member RAS oncogene family pseudogene
RAB8A		√	√	RAB8A, member RAS oncogene family
RALB		√	√	v-ral simian leukemia viral oncogene homolog B (ras related; GTP binding protein)
RB1CC1		√		RB1-inducible coiled-coil 1
RBM19				RNA binding motif protein 19
RNASEL		√		ribonuclease L (2',5'-oligoisoadenylate synthetase-dependent)
RND1				Rho family GTPase 1
RP6-166C19.11				cancer/testis CT47 family, member 11
RSPH10B		√		radial spoke head 10 homolog B (Chlamydomonas)
SDC1		√	√	syndecan 1
SEC61G			√	Sec61 gamma subunit
SEMA5B				sema domain, seven thrombospondin repeats (type 1 and type 1-like), transmembrane domain (TM) and short cytoplasmic domain, (semaphorin) 5B
SERPINB6	√	√		serpin peptidase inhibitor, clade B (ovalbumin), member 6
SHCBP1				SHC SH2-domain binding protein 1
SLC27A6				solute carrier family 27 (fatty acid transporter), member 6
SLC2A13				solute carrier family 2 (facilitated glucose transporter), member 13
SLC38A6				solute carrier family 38, member 6
SLC4A7		√		solute carrier family 4, sodium bicarbonate cotransporter, member 7
SLC9A6	√	√		solute carrier family 9 (sodium/hydrogen exchanger), member 6
SLCO2A1				solute carrier organic anion transporter family, member 2A1
SNF1LK	√	√		SNF1-like kinase
SRMS	√	√		src-related kinase lacking C-terminal regulatory tyrosine and N-terminal myristylation sites
SSR1		√		signal sequence receptor, alpha
SSR3		√		signal sequence receptor, gamma (translocon-associated protein gamma)
SSU72	√	√		SSU72 RNA polymerase II CTD phosphatase homolog (S. cerevisiae)
STIP1			√	stress-induced-phosphoprotein 1
SUCLG2				succinate-CoA ligase, GDP-forming, beta subunit
TATDN1				TatD DNase domain containing 1
TBC1D10A				TBC1 domain family, member 10A

Table 3: continued

Symbol	Druggable HopGrm	Druggable Hogenome	Exp in CD4	Gene Description
TCFL5		√		transcription factor-like 5 (basic helix-loop-helix)
TERF2				telomeric repeat binding factor 2
TGDS				TDP-glucose 4,6-dehydratase
THAP11				THAP domain containing 11
TMED1		√		transmembrane emp24 protein transport domain containing 1
TNK1	√	√		tyrosine kinase, non-receptor, 1
TOM1		√		target of myb1 (chicken)
TPT1				tumor protein, translationally-controlled 1
TRMT6				tRNA methyltransferase 6 homolog (S. cerevisiae)
TRPT1				tRNA phosphotransferase 1
TUBA8	√	√	√	tubulin, alpha 8
TWF1		√	√	twinfilin, actin-binding protein, homolog 1 (Drosophila)
UBE2E1				ubiquitin-conjugating enzyme E2E 1 (UBC4/5 homolog, yeast)
VDR	√	√	√	vitamin D (1,25- dihydroxyvitamin D3) receptor
VPS4A				vacuolar protein sorting 4 homolog A (S. cerevisiae)
WISP2		√		WNT1 inducible signaling pathway protein 2
XPO1		√	√	exportin 1 (CRM1 homolog, yeast)
ZDHHC20				zinc finger, DHHC-type containing 20
ZNF552				zinc finger protein 552
ZNF688				zinc finger protein 688
ZSCAN16				zinc finger and SCAN domain containing 16
BAZ2B		√	√	bromodomain adjacent to zinc finger domain, 2B
BRCA1		√	√	breast cancer 1, early onset
GTF2H1		√	√	general transcription factor IIH, polypeptide 1, 62kDa
HNRNPU	√	√		heterogeneous nuclear ribonucleoprotein U (scaffold attachment factor A)
NRBP1		√		nuclear receptor binding protein 1
PANK3		√		pantothenate kinase 3
PI4KA	√	√		phosphatidylinositol 4-kinase, catalytic, alpha
RAD23A		√	√	RAD23 homolog A (S. cerevisiae)
UBE2Z				ubiquitin-conjugating enzyme E2Z
UQCRC1	√	√		ubiquinol-cytochrome c reductase core protein I
AAK1		√		AP2 associated kinase 1
AFM		√		afamin
ALX1				ALX homeobox 1
ANKRD1				ankyrin repeat domain 1 (cardiac muscle)
APOB	√	√		apolipoprotein B (including Ag(x) antigen)
ARL9				ADP-ribosylation factor-like 9
ASB12				ankyrin repeat and SOCS box-containing 12
ASXL3				additional sex combs like 3 (Drosophila)
BCAS1				breast carcinoma amplified sequence 1

Table 3: continued

Symbol	Druggable HopGrm	Druggable Hogenome	Exp in CD4	Gene Description
C10orf82				chromosome 10 open reading frame 82
C15orf2				chromosome 15 open reading frame 2
C3orf25				chromosome 3 open reading frame 25
CD163	√	√		CD163 molecule
CDO1				cysteine dioxygenase, type I
CFHR5				complement factor H-related 5
CHST13				carbohydrate (chondroitin 4) sulfotransferase 13
CLDN6				claudin 6
CLEC2A				C-type lectin domain family 2, member A
CPN2				carboxypeptidase N, polypeptide 2
CST9L				cystatin 9-like
CYP2C18	√	√		cytochrome P450, family 2, subfamily C, polypeptide 18
CYP4F11	√	√		cytochrome P450, family 4, subfamily F, polypeptide 11
DEFB131				defensin, beta 131
FATE1				fetal and adult testis expressed 1
KCNK10		√		potassium channel, subfamily K, member 10
LOC100129029				hypothetical LOC100129029
LOC100129840				hypothetical protein LOC100129840
LOC100131089				hypothetical protein LOC100131089
GALNTL4				UDP-N-acetyl-alpha-D-galactosamine:polypeptide N-acetylgalactosaminyltransferase-like 4
GRIA2	√	√		glutamate receptor, ionotropic, AMPA 2
GTF3C3			√	general transcription factor IIIC, polypeptide 3, 102kDa
MIRN137				microRNA 137
IGKC				immunoglobulin kappa constant
IL22RA1		√		interleukin 22 receptor, alpha 1
ILDR1		√		immunoglobulin-like domain containing receptor 1
KIAA1920				KIAA1920 protein
KRTCAP3				keratinocyte associated protein 3
TTC23L				tetratricopeptide repeat domain 23-like
LOC554207				hypothetical LOC554207
LRRIQ1				leucine-rich repeats and IQ motif containing 1
MAGEA3				melanoma antigen family A, 3
MIRN181B2				microRNA 181b-2
MIRN210				microRNA 210
MIRN372				microRNA 372
MIRN93				microRNA 93
MIRN98				microRNA 98
MIXL1				Mix1 homeobox-like 1 (Xenopus laevis)
MLANA				melan-A
MOGAT1				monoacylglycerol O-acyltransferase 1

Table 3: continued

Symbol	Druggable HopGrm	Druggable Hogenome	Exp in CD4	Gene Description
NOL3				nucleolar protein 3 (apoptosis repressor with CARD domain)
NR4A2	√	√		nuclear receptor subfamily 4, group A, member 2
OLFML2B		√		olfactomedin-like 2B
ONECUT1				one cut homeobox 1
OR52A1	√	√		olfactory receptor, family 52, subfamily A, member 1
OTOP1				otopetrin 1
PCDHA2				protocadherin alpha 2
PLA1A	√	√		phospholipase A1 member A
RGAG4				retrotransposon gag domain containing 4
RHOD				ras homolog gene family, member D
SLC10A1	√	√		solute carrier family 10 (sodium/bile acid cotransporter family), member 1
SLC1A7				solute carrier family 1 (glutamate transporter), member 7
SLC25A27	√	√		solute carrier family 25, member 27
SPATA4				spermatogenesis associated 4
SRD5A2		√		steroid-5-alpha-reductase, alpha polypeptide 2 (3-oxo-5 alpha-steroid delta 4-dehydrogenase alpha 2)
TBPL2				TATA box binding protein like 2
TGFB3		√		transforming growth factor, beta 3
THOC6				THO complex 6 homolog (Drosophila)
TMC1				transmembrane channel-like 1
TNFSF11		√	√	tumor necrosis factor (ligand) superfamily, member 11
TRIM42				tripartite motif-containing 42
TRPC4	√	√		transient receptor potential cation channel, subfamily C, member 4
TRPV6	√	√		transient receptor potential cation channel, subfamily V, member 6
WFDC9				WAP four-disulfide core domain 9
TSG101		√	√	tumor susceptibility gene 101

5 Table of genes that appear in two or more siRNA screens

Table 4: Table of 34 Genes that appear in two or more siRNA screens.

Symbol	Frequency	siRNA HIV König	siRNA HIV Brass	siRNA HIV Zhou	SNP HIV Fellay	Particle Associated HIV	HARC Nef	HARC Tat	HARC Rev	BIND HIV IN	NCBI Interactions	siRNA Flu Fly	siRNA WNV	Druggable HopGrm	Druggable Hogenome	Exp in CD4	Gene Description
ADRBK1	2	√		√										√	√	√	adrenergic, beta, receptor kinase 1
AKT1	2		√	√							√			√	√	√	v-akt murine thymoma viral oncogene homolog 1
CAV2	2		√	√											√	√	caveolin 2
CCNT1	2		√	√				√			√				√		cyclin T1
CD4	2		√	√							√				√	√	CD4 molecule
DDX3X	2		√	√				√	√		√					√	DEAD (Asp-Glu-Ala-Asp) box polypeptide 3, X-linked
DMXL1	2	√	√													√	Dmx-like 1
IDH1	2	√	√													√	isocitrate dehydrogenase 1 (NADP+), soluble
JAK1	2		√	√							√			√	√	√	Janus kinase 1 (a protein tyrosine kinase)
MAP4	2	√	√													√	microtubule-associated protein 4
MRE11A	2	√		√											√	√	MRE11 meiotic recombination 11 homolog A (S. cerevisiae)
RANBP2	2	√	√											√	√	√	RAN binding protein 2
TCEB3	2		√	√							√					√	transcription elongation factor B (SIII), polypeptide 3 (110kDa, elongin A)
CXCR4	2		√	√							√			√	√	√	chemokine (C-X-C motif) receptor 4
CHST1	2	√		√							√					√	carbohydrate (keratan sulfate Gal-6) sulfotransferase 1
CTDP1	2	√	√								√				√	√	CTD (carboxy-terminal domain, RNA polymerase II, polypeptide A) phosphatase, subunit 1
MED14	2	√	√												√	√	mediator complex subunit 14
RAB28	2		√	√											√	√	RAB28, member RAS oncogene family
NUP153	2	√	√								√	√		√	√	√	nucleoporin 153kDa
TNPO3	2	√	√												√	√	transportin 3

Table 4: continued

Symbol	**Frequency**	siRNA HIV König	siRNA HIV Brass	siRNA HIV Zhou	SNP HIV Fellay	Particle Associated HIV	HARC Nef	HARC Tat	HARC Rev	BIND HIV IN	NCBI Interactions	siRNA Flu Fly	siRNA WNV	**Druggable HopGrm**	**Druggable Hogenome**	**Exp in CD4**	**Gene Description**
MED4	2		√	√											√	√	mediator complex subunit 4
ANAPC2	2	√		√											√	√	anaphase promoting complex subunit 2
MID1IP1	2	√	√										√			√	MID1 interacting protein 1 (gastrulation specific G12 homolog (zebrafish))
WNK1	2		√	√											√	√	WNK lysine deficient protein kinase 1
RNF26	2		√	√											√		ring finger protein 26
MED28	2		√	√												√	mediator complex subunit 28
TRIM55	2	√	√														tripartite motif-containing 55
ANKRD30A	2		√	√										√	√		ankyrin repeat domain 30A
MED19	2	√		√											√	√	mediator complex subunit 19
HMCN2	2	√		√													hemicentin 2
RGPD8	2		√	√												√	RANBP2-like and GRIP domain containing 8
RELA	3	√	√	√							√			√	√	√	v-rel reticuloendotheliosis viral oncogene homolog A (avian)
MED7	3	√	√	√											√	√	mediator complex subunit 7
MED6	3	√	√	√											√	√	mediator complex subunit 6

6 Analysis of all Genome-wide screens versus the NCBI interactions list

- **Genome Wide screens:** siRNA HIV König, siRNA HIV Brass, siRNA HIV Zhou, SNP HIV Fellay, Particle Associated HIV, HARC Nef, HARC Tat, HARC Rev, BIND HIV IN

- **Total Genes :** 1344

- **Total Unique Genes (Union) :** 1254
- **Total Protein Coding Genes :** 25157
- **Total Genes in NCBI Interactions :** 1434
- **Overlap of Union VS NCBI Interactions :** 258
- **Union of Genome Wide Screens and NCBI Interactions :** 2430
- **Hypergeometric p-value:** <0.001
- **Simulation p-value:**
  Number of counts that had equal to or greater overlap than (258) in 1000 permutations: 0 => p-value: <0.001

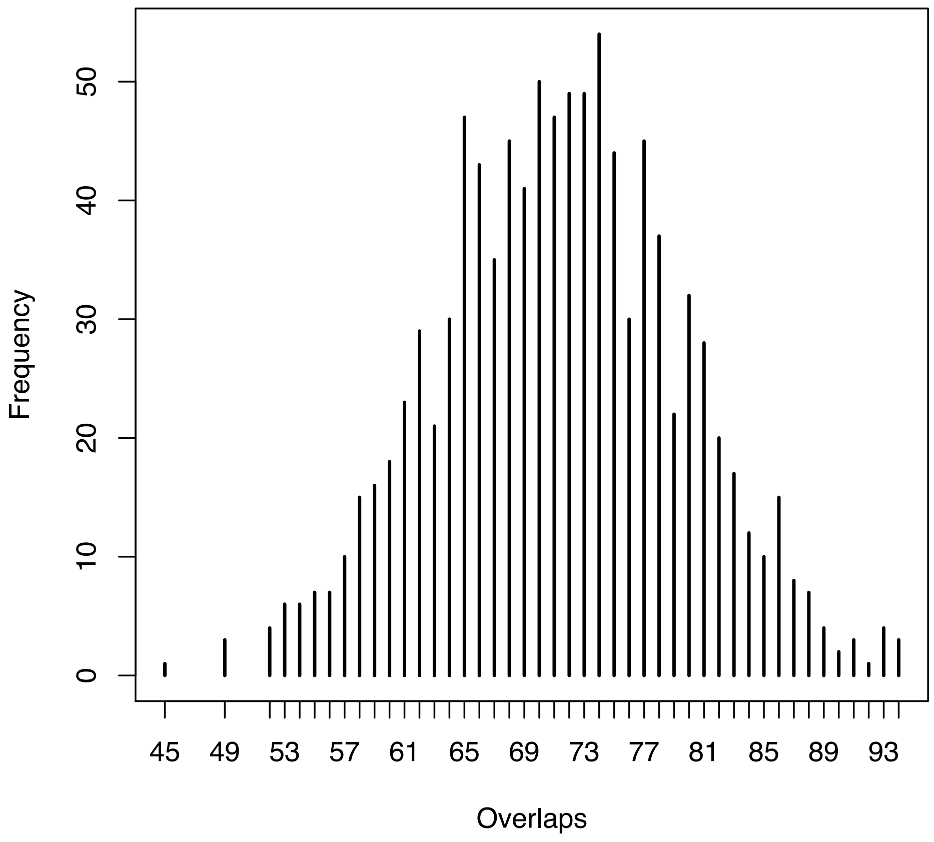

7 Table summarizing all pairwise overlaps between screens

Table 5: Table of List comparisons and their respective simulation p-values (overlaps).

**Table Name(Size)**	siRNA HIV König (293)	siRNA HIV Brass (283)	siRNA HIV Zhou (303)	SNP HIV Fellay (63)	Particle Associated HIV (248)	HARC Nef (6)	HARC Tat (69)	HARC Rev (56)	BIND HIV IN (23)	NCBI Interactions (1434)	siRNA Flu Fly (98)	siRNA WNV (305)
siRNA HIV König (293)												
siRNA HIV Brass (283)	<0.001 (13)											
siRNA HIV Zhou (303)	0.024 (9)	<0.001 (18)										
SNP HIV Fellay (63)	1 (0)	0.511 (1)	0.541 (1)									
Particle Associated HIV (248)	0.154 (5)	0.035 (6)	0.07 (6)	0.108 (2)								
HARC Nef (6)	1 (0)	1 (0)	1 (0)	1 (0)	<0.001 (2)							
HARC Tat (69)	1 (0)	0.004 (4)	0.052 (3)	1 (0)	0.027 (3)	1 (0)						
HARC Rev (56)	0.125 (2)	0.44 (1)	0.469 (1)	1 (0)	<0.001 (10)	1 (0)	1 (0)					
BIND HIV IN (23)	<0.001 (3)	1 (0)	0.232 (1)	1 (0)	0.191 (1)	1 (0)	1 (0)	0.07 (1)				
NCBI Interactions (1434)	<0.001 (53)	<0.001 (39)	<0.001 (40)	0.234 (5)	<0.001 (94)	1 (0)	<0.001 (30)	<0.001 (21)	0.009 (5)			
siRNA Flu Fly (98)	<0.001 (13)	0.125 (3)	0.738 (1)	1 (0)	<0.001 (9)	1 (0)	1 (0)	0.002 (3)	1 (0)	<0.001 (20)		
siRNA WNV (305)	0.02 (8)	0.004 (9)	0.693 (3)	0.14 (2)	0.013 (8)	1 (0)	0.061 (3)	0.481 (1)	1 (0)	0.006 (29)	0.337 (2)	

Table 6: Table of List comparisons and their respective hypergeometric p-values (overlaps).

**Table Name(Size)**	siRNA HIV König (293)	siRNA HIV Brass (283)	siRNA HIV Zhou (303)	SNP HIV Fellay (63)	Particle Associated HIV (248)	HARC Nef (6)	HARC Tat (69)	HARC Rev (56)	BIND HIV IN (23)	NCBI Interactions (1434)	siRNA Flu Fly (98)	siRNA WNV (305)
siRNA HIV König (293)												
siRNA HIV Brass (283)	<0.001 (13)											
siRNA HIV Zhou (303)	0.014 (9)	<0.001 (18)										
SNP HIV Fellay (63)	1 (0)	0.167 (1)	0.201 (1)									
Particle Associated HIV (248)	0.064 (5)	0.015 (6)	0.028 (6)	0.02 (2)								
HARC Nef (6)	1 (0)	1 (0)	1 (0)	1 (0)	<0.001 (2)							
HARC Tat (69)	1 (0)	0.001 (4)	0.008 (3)	1 (0)	0.005 (3)	1 (0)						
HARC Rev (56)	0.024 (2)	0.111 (1)	0.136 (1)	1 (0)	<0.001 (10)	1 (0)	1 (0)					
BIND HIV IN (23)	<0.001 (3)	1 (0)	0.029 (1)	1 (0)	0.021 (1)	1 (0)	1 (0)	0.001 (1)				
NCBI Interactions (1434)	<0.001 (53)	<0.001 (39)	<0.001 (40)	0.116 (5)	<0.001 (94)	1 (0)	<0.001 (30)	<0.001 (21)	0.001 (5)			
siRNA Flu Fly (98)	<0.001 (13)	0.034 (3)	0.412 (1)	1 (0)	<0.001 (9)	1 (0)	1 (0)	<0.001 (3)	1 (0)	<0.001 (20)		
siRNA WNV (305)	0.008 (8)	0.001 (9)	0.481 (3)	0.034 (2)	0.003 (8)	1 (0)	0.009 (3)	0.144 (1)	1 (0)	0.003 (29)	0.115 (2)	

8 References

1. Konig, R., Zhou, Y., Elleder, D. et al. (2008) Cell 135, 49-60
2. Butler, S., Hansen, M., and Bushman, F. D. (2001) 7, 631-634
3. O'Doherty, U., Swiggard, W. J., Jeyakumar, D., McGain, D., and Malim, M. H. (2002) 76, 10942-10950
4. Brass, A. L., Dykxhoorn, D. M., Benita, Y., Yan, N., Engelman, A., Xavier, R. J., Lieberman, J., and Elledge, S. J. (2008) Science 319, 921-926
5. Zhou, H., Xu, M., Huang, Q. et al. (2008) Cell. Host Microbe
6. Fellay, J., Shianna, K. V., Ge, D. et al. (2007) Science 317, 944-947
7. Chertova, E., Chertov, O., Coren, L. V. et al. (2006) J. Virol. 80, 9039-9052
8. Garrus, J. E., von Schwedler, U. K., Pornillos, O. W. et al. (2001) 107, 55-65.
9. Strack, B., Calistri, A., Craig, S., Popova, E., and Gottlinger, H. G. (2003) Cell 114, 689-699
10. Bounou, S., Leclerc, J. E., and Tremblay, M. J. (2002) J. Virol. 76, 1004-1014
11. Butini, L., De Fougerolles, A. R., Vaccarezza, M., Graziosi, C., Cohen, D. I., Montroni, M., Springer, T. A., Pantaleo, G., and Fauci, A. S. (1994) Eur. J. Immunol. 24, 2191-2195
12. Leung, K., Kim, J. O., Ganesh, L., Kabat, J., Schwartz, O., and Nabel, G. J. (2008) Cell. Host Microbe 3, 285-292
13. Nguyen, D. H., and Hildreth, J. E. (2000) J. Virol. 74, 3264-3272
14. Manes, S., del Real, G., Lacalle, R. A., Lucas, P., Gomez-Mouton, C., Sanchez-Palomino, S., Delgado, R., Alcami, J., Mira, E., and Martinez-A, C. (2000) EMBO Rep. 1, 190-196
15. Studamire, B., and Goff, S. P. (2008) Retrovirology 5, 48
16. Hao, L., Sakurai, A., Watanabe, T., Sorensen, E., Nidom, C. A., Newton, M. A., Ahlquist, P., and Kawaoka, Y. (2008) Nature 454, 890-893
17. Krishnan, M. N., Ng, A., Sukumaran, B. et al. (2008) Nature 455, 242-245
